# Supplementary material for: Translation stress and collided ribosomes are co-activators of cGAS
Source: Mol Cell. 2021 Jul 1;81(13):2808–2822.e10. doi: 10.1016/j.molcel.2021.05.018 (PMC8260207; doi:10.1016/j.molcel.2021.05.018)
Supplement: Document S1. Figures S1–S8 [file mmc1.pdf]

Molecular Cell, Volume *81*

Supplemental information

Translation stress and collided ribosomes

are co-activators of cGAS

Li Wan, Szymon Juszkiewicz, Daniel Blears, Prashanth Kumar Bajpe, Zhong Han, Peter Faull, Richard Mitter, Aengus Stewart, Ambrosius P. Snijders, Ramanujan S. Hegde, and Jesper Q. Svejstrup

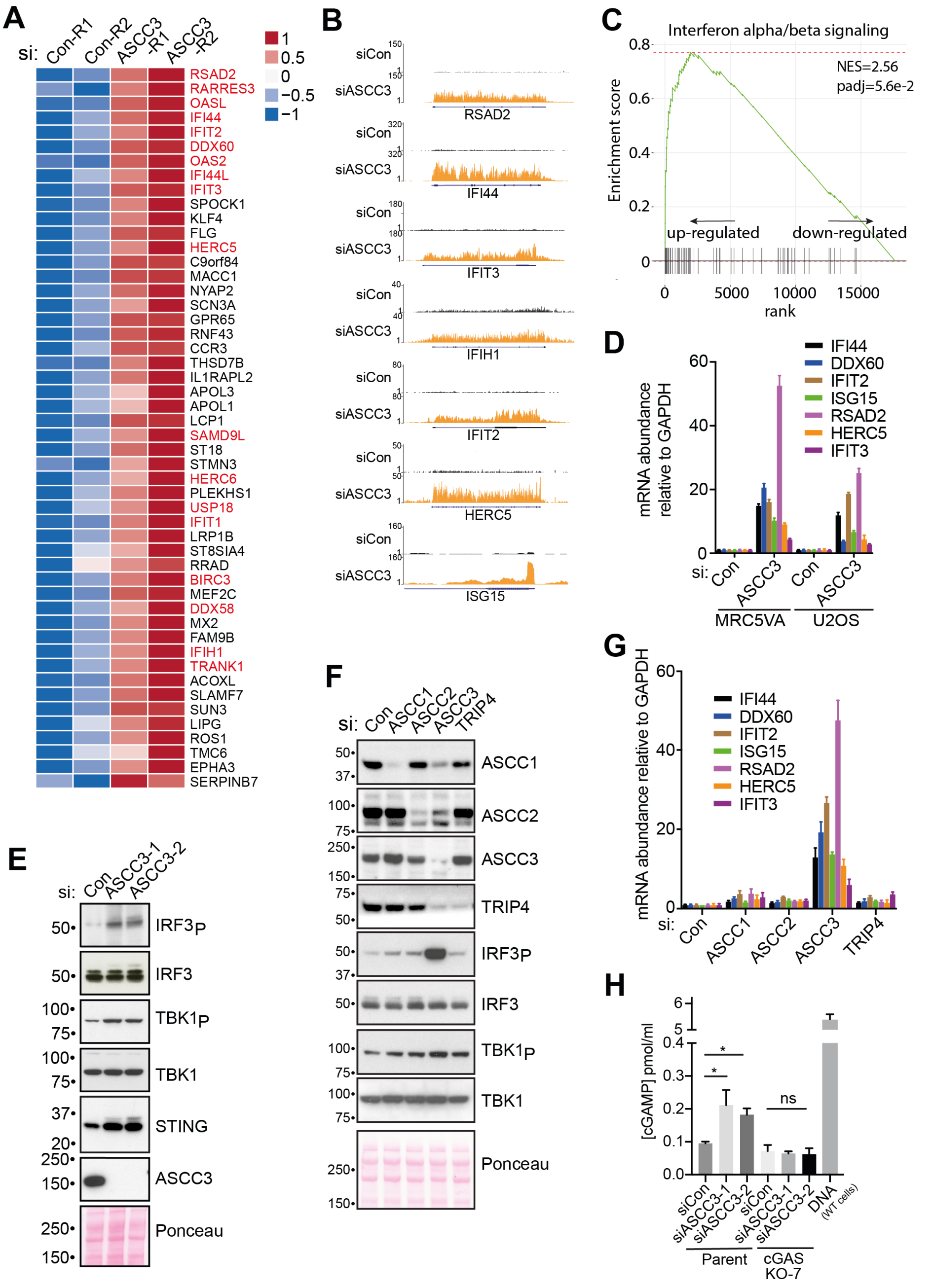

Figure S1. Knockdown of ASCC3 induces the expression of ISGs. Related to Figure 1. A. Heat map of the 50 most up-regulated genes at the level of nascent transcription upon ASCC3 siRNA knockdown (ASCC3kd) in MRC5VA cells. R1, Replicate 1; R2, Replicate 2. The heatmap represents a z-score of normalised expression. ISGs are marked in red. B. UCSC genome browser views of nascent transcription at individual ISGs. Read depth coverage was combined across two replicates, normalised to yeast spike-in. C. GSEA category enrichment plot of the Reactome category 'Interferon alpha/beta signalling'. NES, normalised enrichment score; padj, adjusted p-value. D. qRT-PCR analysis of ISG mRNA expression in MRC5VA or U2OS cells after ASCC3kd. Error bars represent standard deviation (SD) of three technical replicates and are representative of three biological repeats. E. Western blot analysis of IRF3p (ser396), IRF3, TBK1p (ser172), STING ASCC3 protein level in MRC5VA cells transfected with ASCC3 siRNAs. F. Western blot analysis of MRC5VA cells after siRNA knockdown of ASCC subunits. G qRT-PCR analysis of relative ISG expression from the same cells as in F. Error bars represent SD of three technical replicates and are representative of three biological repeats. H. ELISA analysis of cGAMP concentration in MRC5VA parental or cGAS KO cells transfected with the indicated siRNAs. Two-tailed t-test. *p < 0.05; ns: not significant. Error bars represent SEM (three replicates). MRC5VA parental cells transfected with HT-DNA served as a positive control.

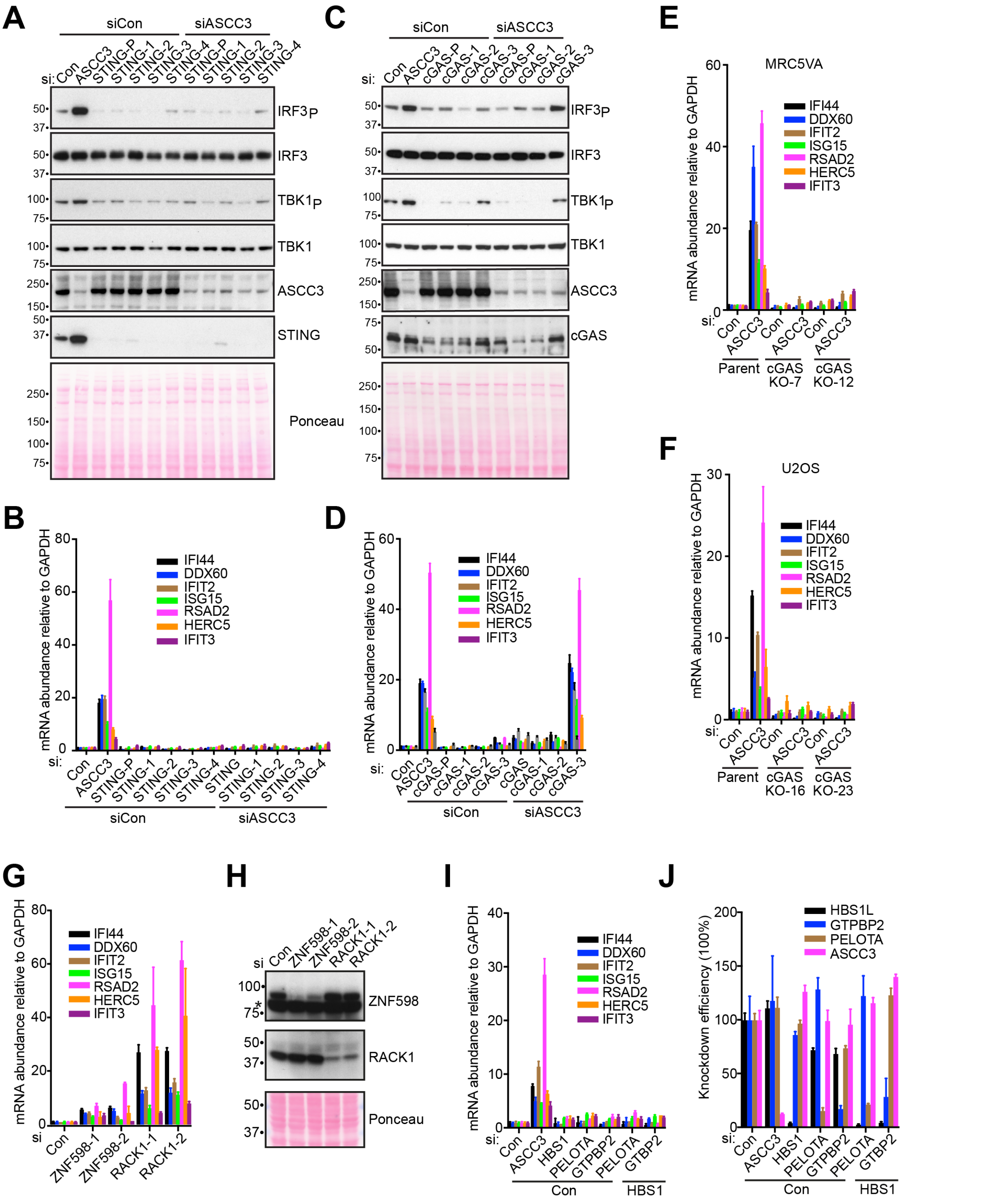

Figure S2. cGAS and STING are required for activation of ISGs. Related to Figure 1. A. Western blot analysis of MRC5VA cells after siRNA knockdown. STING-P, STING siRNA pool. **B.** qRT-PCR analysis of ISG expression in the same cells as in A. Error bars represent SD of three technical replicates and are representative of three biological repeats. **C.** As in A, but transfection with different siRNAs. cGAS-P, cGAS siRNA pool. **D.** As in B, but transfection with different siRNAs. **E.** qRT-PCR analysis of ISG expression in parental and *CGAS* knockouts (KO-7 and -12) MRC5VA cells, after ASCC3 knockdown. Error bars as in B. **F.** As in E, but in U2OS cells (parental versus *CGAS* KO-16 and -23). **G.** qRT-PCR analysis of ISG expression in U2OS cells transfected with two different ZNF598 or RACK1 siRNAs. Error bars as in B. **H.** Western blot analysis of ZNF598 and RACK1 protein levels in the same cells as in G. Asterisk denotes a non-specific band. **I.** qRT-PCR analysis of ISG expression in U2OS cells transfected with siRNAs. Error bars as in B. **J.** qRT-PCR analysis of knockdown efficiency for the indicated genes in the same cells as in I. Normalized to GAPDH mRNA as internal control.

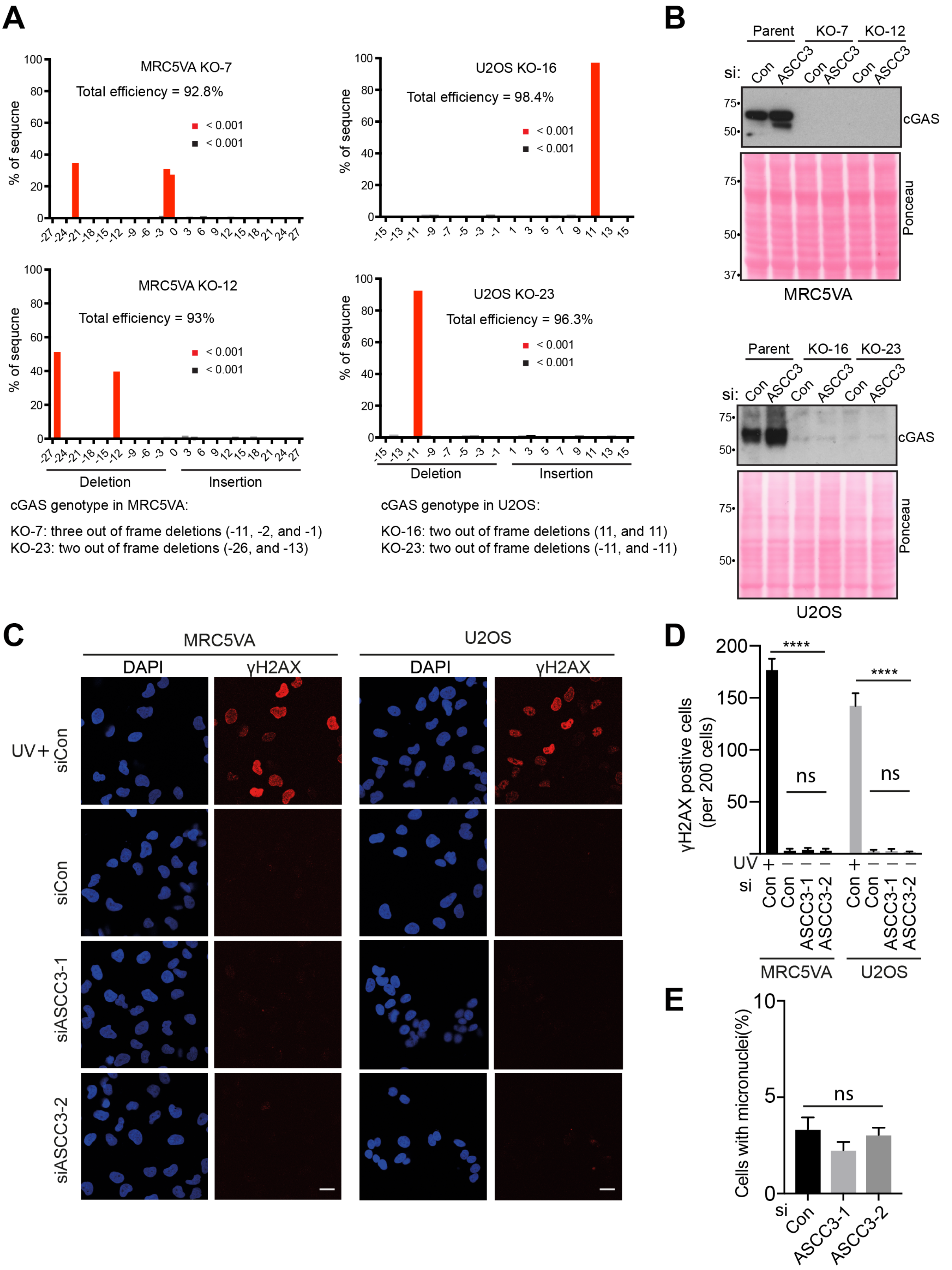

Figure S3. cGAS KO and lack of effect of ASCC3 on DNA damage markers. Related to Figure 1. **A.** Tracking of Indels by TIDE analysis of cGAS knockouts in MRC5VA or U2OS cells. **B.** Western blot analysis of the cGAS knockouts. **C.** MRC5VA or U2OS cells were transfected with ASCC3 siRNAs. Cells were fixed, and stained with anti-γH2AX antibody and with DAPI, and imaged by confocal fluorescence microscopy. Scale bar: 10 μm. **D.** Quantitative analysis of gH2AX-positive cells from C. Mean with SEM, 200 cells analysed per condition per experiment, n=3 independent experiments. Two-tailed t test, ns, no significant; ****, P < 0.0001. **E.** The Percentage of U2OS cells containing micronuclei. Mean with SEM, 400 cells analysed per condition per experiment, n=6 independent experiments. Two-tailed t test. ns, not significant.

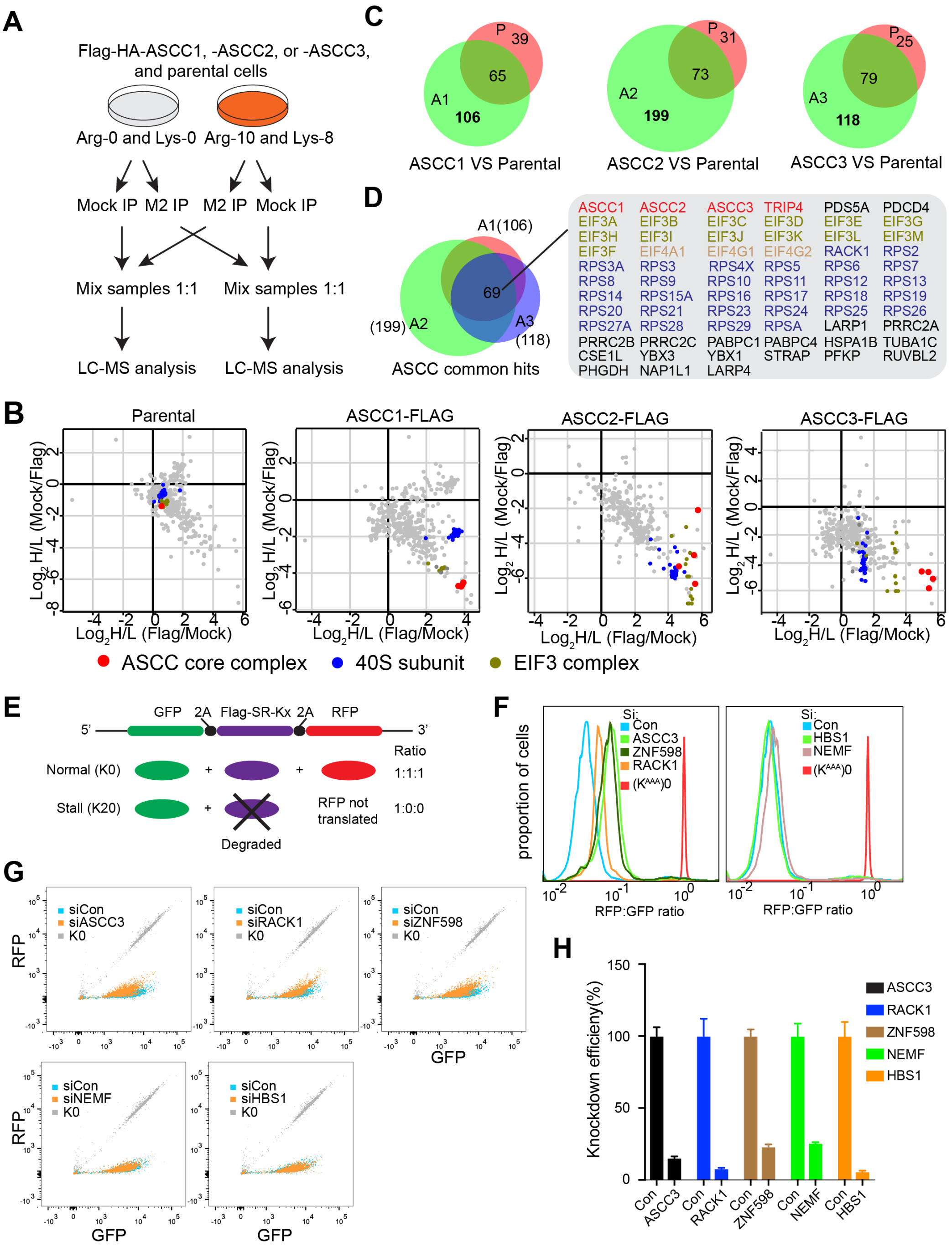

Figure S4. ASCC interactome, and effect of RQC factors on translation. Related to Figure 1. A. Strategy of SILAC-based quantitative mass spectrometry. **B.** Scatter plots showing interactomes in control (parental cells), Flag-HA-ASCC1, -ASCC2, and -ASCC3 HEK293 cells. Perseus was used for all proteomic data analysis. **C.** Overlap of interactors between control and experimental cells; hits from the ASCC1, ASCC2, or ASCC3 interactomes that were not found in the control were used for further analysis in D. See STAR Methods for details and lists of hits in Table S1. **D. Left,** Venn diagram and **Right,** the 69 overlapping hits from the ASCC1, ASCC2, and ASCC3 interactomes. ASCC subunits, small ribosomal proteins, and EIF3 complex subunits are indicated. **E.** Reporter construct and expected protein products with or without stalling signal. Flag-SR-Kx is a stalling sequence in which x represents either twenty or zero lysines. **F.** Histograms of the RFP:GFP ratios quantified by flow cytometry in the $(K^{AAA})_{20}$ HEK293 cells transfected with indicated siRNA. $(K^{AAA})_{0}$ cells were used as a reference. For simplicity, cells transfected with siRNAs for control, ASCC3, ZNF598, or RACK1 are shown in the left panel, while those for HBS1, or NEMF are shown on the right. **G.** Scatterplot showing individual $(K^{AAA})_{20}$ HEK293 cells transfected with indicated siRNAs. $(K^{AAA})_{0}$ cells were used as a reference. **H.** RT-qPCR analysis of knockdown efficiency for experiments in F and G. Error bars represent SD of three technical replicates and are representative of two biological replicates.

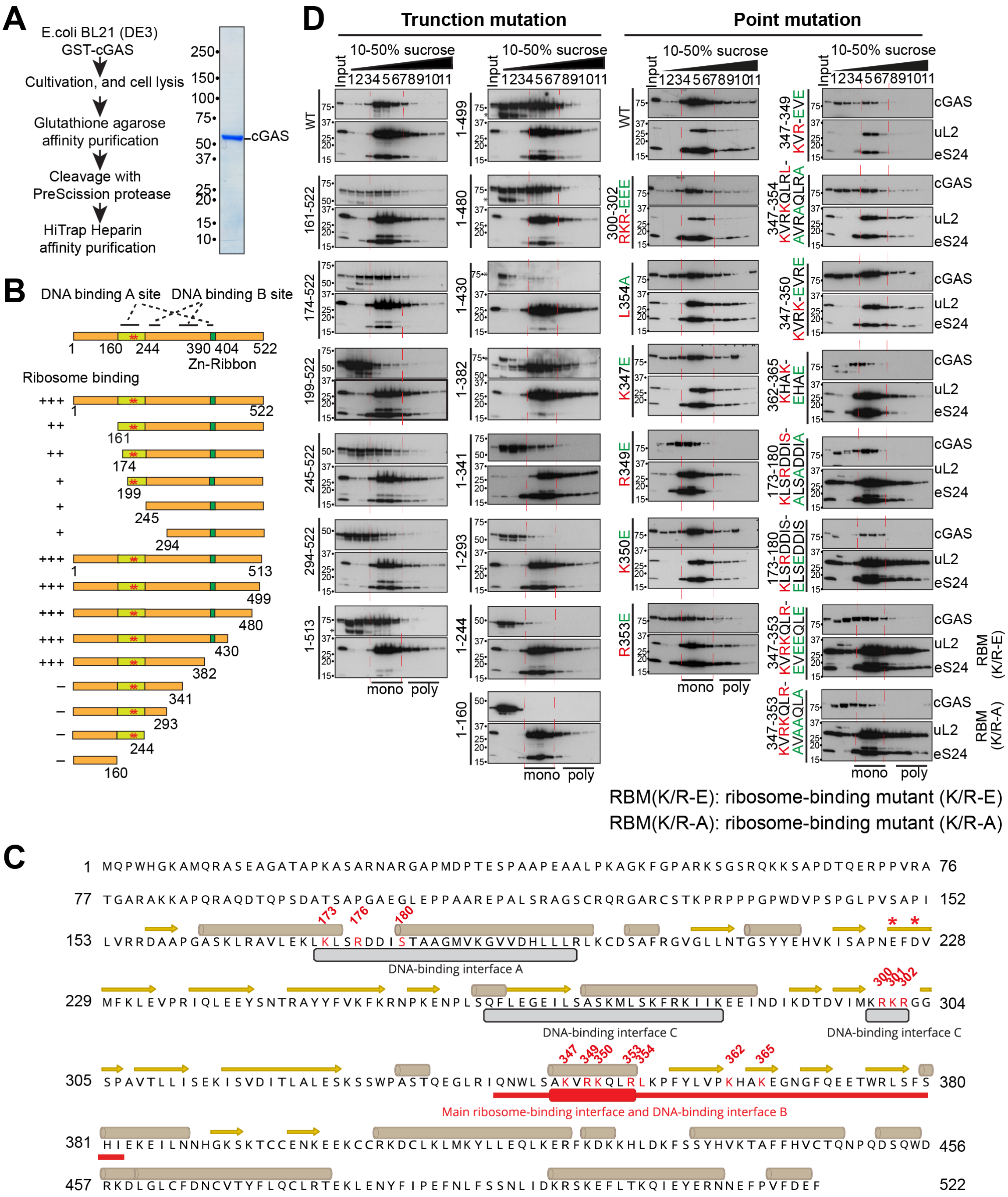

Figure S5. Purification of cGAS and characterization of its ribosome binding domains. Related to Figure 3. A. Diagram for purification; and Coomassie staining of purified cGAS. **B.** Schematic of cGAS deletion mutants for testing the interaction with ribosomes. Red asterisks indicate the active site. +++ , strong binding; ++, medium binding; +, weak binding; -, little or no binding. **C.** Sequence of cGAS and point mutants for testing its interaction with ribosomes. Charged amino acids marked in red were mutated to either alanine (A) or glutamic acid (E). The region most important for cGAS-ribosome interaction is indicated by a red line, and a key area containing several important K and R residues is shown as a red box. Red asterisks (E_{225} and D_{227}) indicate the active site, while the light brown tubes indicate alpha-helices and the yellow arrows beta-sheets. **D.** Western blot analysis of sucrose gradient sedimentation of cGAS-ribosome interactions. Fractions were immunoblotted for GFP-cGAS and for representative ribosome subunits (ul2 and eS24). Asterisks denote non-specific bands. Note that while K/R point mutations in regions 173-180 and 300-302 have little or no effect, mutation of residues in 362-365 and especially 347-353 results in reduced binding. Mutation of positively charged residues in 347-353 to alanine in the context of full length cGAS results in marked decrease in binding so that the binding profile is similar to cGAS 1-341. These mutations also abolish ISG activation upon ASCC3 knockdown.

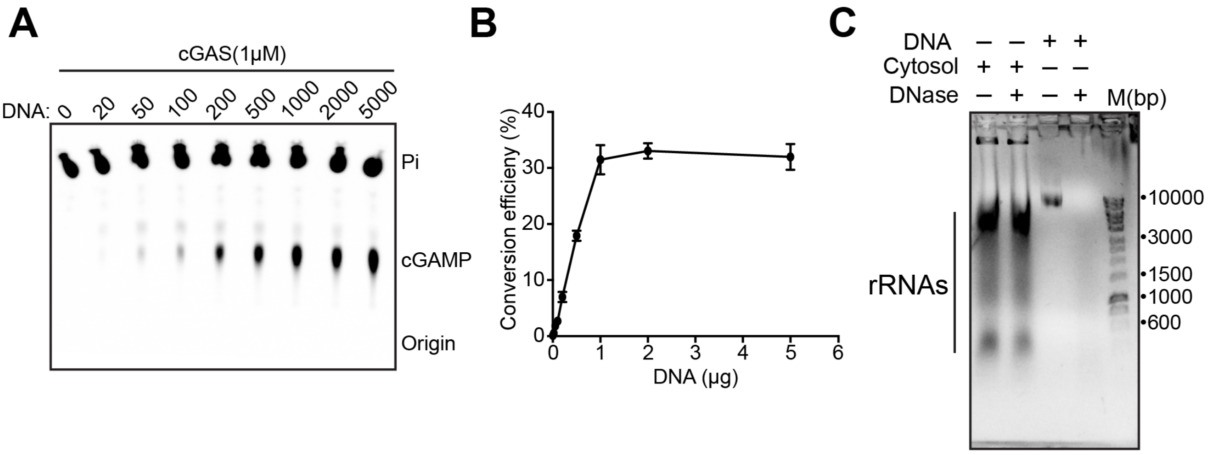

Figure S6. cGAS assays and controls. Related to Figure 4. A. Autoradiograph of cGAS-mediated cGAMP synthesis in the presence of different concentrations of herring testis DNA. **B.** Quantification of data in A by Fiji. Error bars indicate standard deviation (SD) of duplicate replicates. cGAMP signal was normalized to pi signal. **C.** Gel electrophoresis of HEK293 cytosol or 200 ng of HT-DNA digested with 0.05 U TURBO DNase under the conditions used for purifying DNase-treated ribosomes. Note that there is, not surprisingly, no DNA is detectable by this approach in the cytosol, so that no difference between treated or untreated cytosol can be observed.

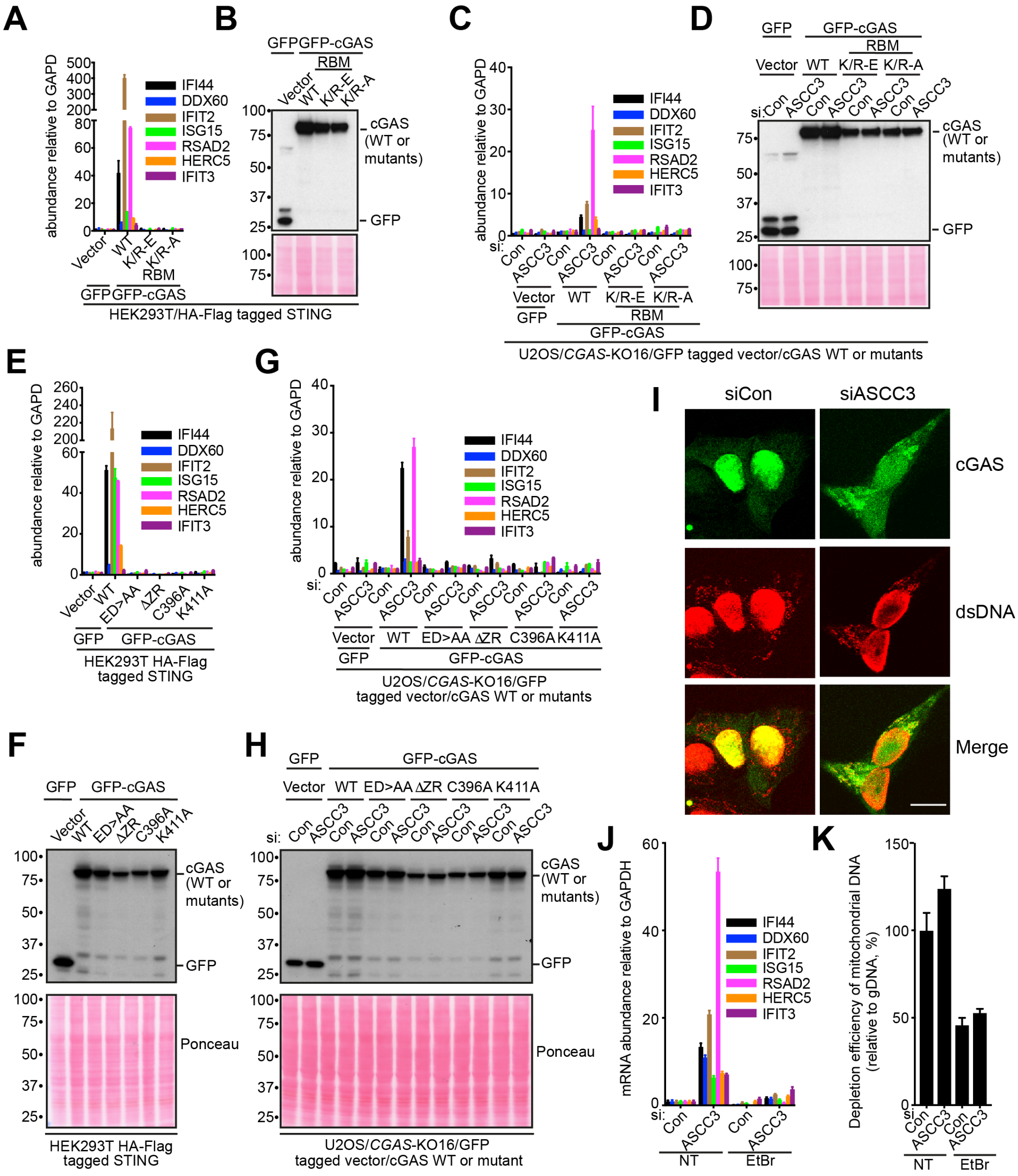

Figure S7. The ribosome-binding interface, zinc-ribbon domain and DNA binding domain of cGAS remain critical for cGAS activation in ASCC3-deficienct cells. Related to Figure 4. A. ISG expression analysis by qRT-PCR of HEK293T cells stably expressing HA-Flag-tagged STING, after transfection of plasmids encoding GFP-vector control, GFP-tagged wild type cGAS (WT), or cGAS ribosome-binding mutants: RBM (K-E) or RBM (K-A) referring to Figure S5D. See STAR Methods for details. **B.** Western blot analysis of whole cell lysate from A to check protein expression levels using anti-GFP antibody. **C.** qRT-PCR analysis of ISG expression in U2OS *CGAS* KO-16 cells and the effect of expression of different cGAS versions, with or without ASCC3 knockdown as indicated. **D.** Western blot analysis of whole cell lysate from C to check protein expression levels using anti-GFP antibody. **E.** As in A, but transfection of plasmids encoding GFP-vector control, GFP-tagged wild type cGAS (WT), or the indicated cGAS: ED>AA (E225A and D227A) is an active site mutant; ΔZR (Δ390-404) deletes the zinc ribbon domain, and C396A is a zinc-ribbon domain point mutant, K411A is a DNA binding site mutant. **F.** Western blot analysis of whole cell lysate from E to check protein expression levels using anti-GFP antibody. **G.** As in C, but stable expression of different cGAS mutants, with or without ASCC3 knockdown as indicated. **H.** Western blot analysis of cell lysates from G, as in F. **I.** U2OS cells stably expressing GFP-cGAS were transfected with ASCC3 siRNA. Cells were fixed, stained with antibody detecting double-stranded DNA, and imaged by confocal fluorescence microscopy. Scale bar: 10 µm. Note the staining in the cytoplasm. **J.** qRT-PCR analysis of ISG expression in MRC5VA cells treated with ethidium bromide (EtBr, 100 ng/ml for 96 hours), after ASCC3 knockdown. **K.** qRT-PCR analysis of depletion efficiency of mitochondrial DNA in the same cells as in J. gDNA, genomic DNA.

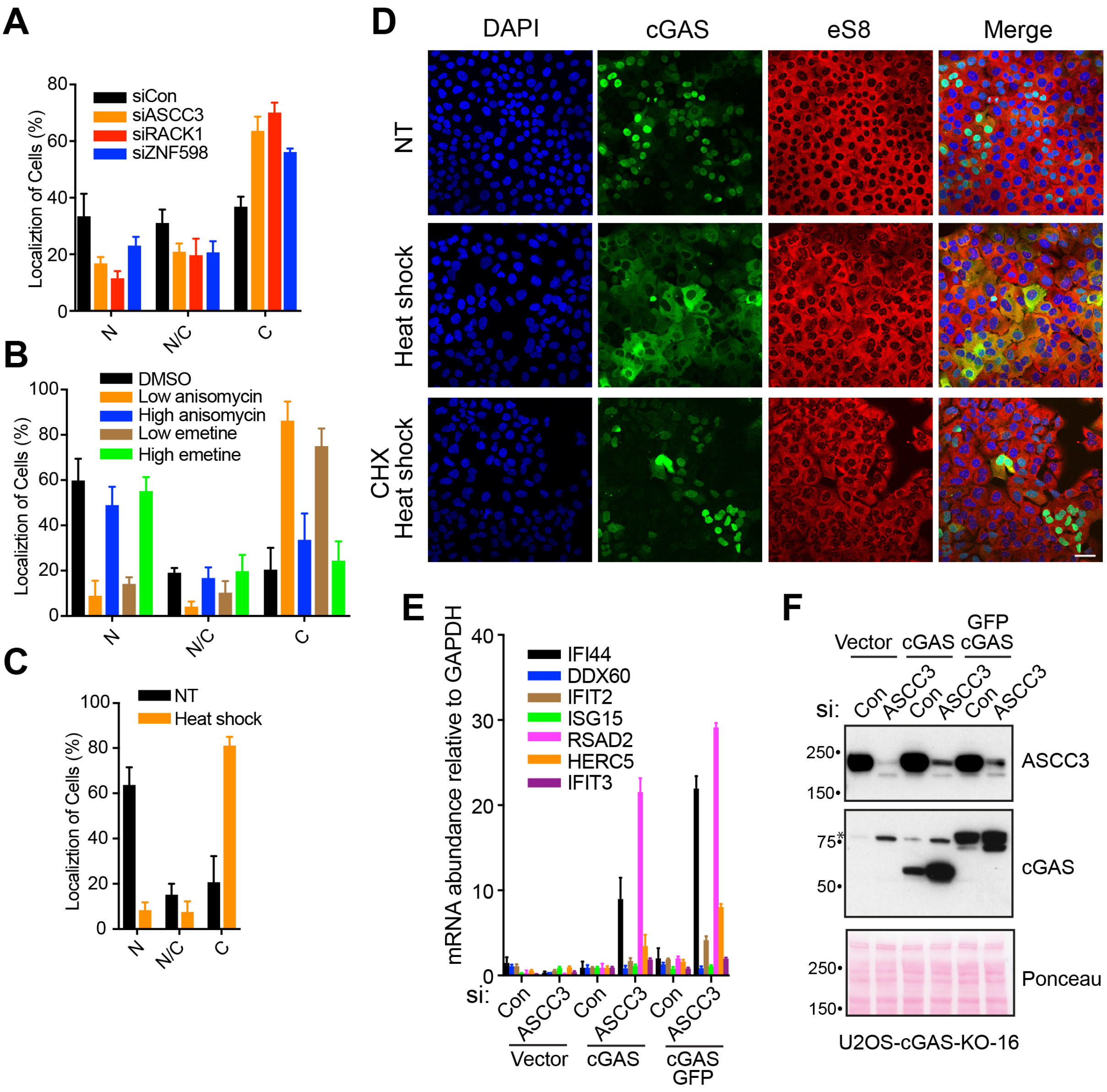

Figure S8. cGAS preferentially binds to collided ribosomes and becomes cytosolic upon ribosome collision. Related to Figure 6. A. Quantitative analysis of the subcellular localization of GFP-cGAS from Figure 6A. 200 cells were analyzed for each sample, error bars represent SD of three biological replicates. N, nucleus; N/C, nucleus and cytosol; C, cytosol. **B.** As in A., but analyzing the data in Figure 6B. **C.** As in A., but analyzing the data in Figure 6C. **D.** U2OS *cGAS* KO cells stably expressing GFP-tagged cGAS were treated with heat shock in the absence or presence of cycloheximide (CHX). Cells were fixed, and stained with eS8 antibody and with DAPI, and imaged by confocal fluorescence microscopy. Scale bar: 50 µm. **E.** qRT-PCR analysis of relative ISG expression in U2OS *CGAS* KO stably expressing untagged or GFP-tagged cGAS cells transfected with ASCC3 siRNA. Error bars represent SD of three technical replicates and are representative of three biological replicates. **F.** Western analysis of ASCC3 and cGAS in the same cells as in E. Asterisk denotes a non-specific band.
