# Supplementary material for: Translation stress and collided ribosomes are co-activators of cGAS
Source: Mol Cell. 2021 Jul 1;81(13):2808–2822.e10. doi: 10.1016/j.molcel.2021.05.018 (PMC8260207; doi:10.1016/j.molcel.2021.05.018)
Supplement: Document S2. Article plus supplemental information [file mmc5.pdf]

Molecular Cell

Article

Translation stress and collided ribosomes are co-activators of cGAS

Graphical abstract

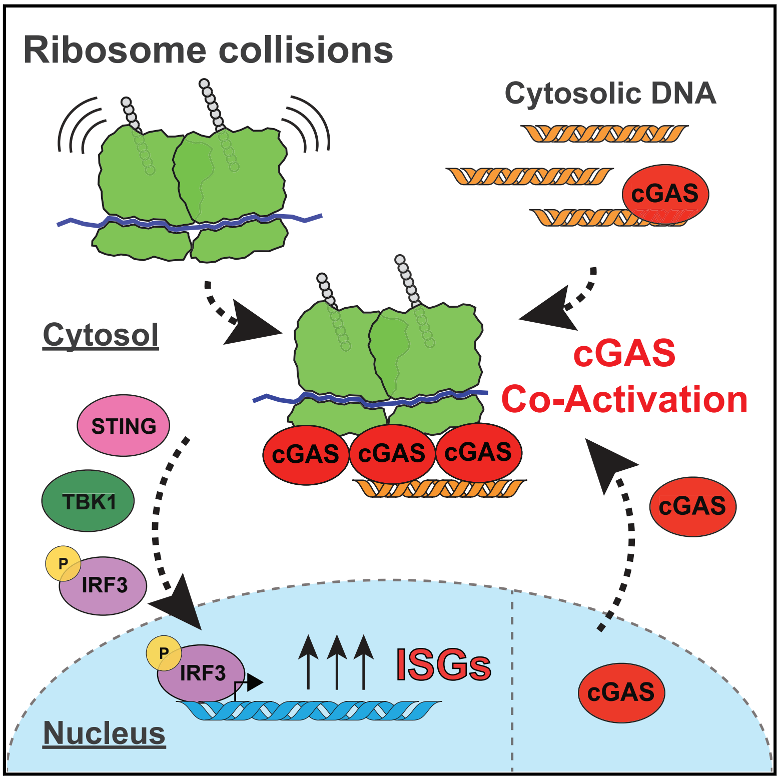

Authors

Li Wan, Szymon Juszkiewicz, Daniel Blears, ..., Ambrosius P. Snijders, Ramanujan S. Hegde, Jesper Q. Svejstrup

Correspondence

jsvejstrup@sund.ku.dk

In brief

Wan et al. show that cGAS, a well-known DNA sensor, can also sense translation stress by direct interaction with ribosomes, which in turn induces accumulation of cGAS in the cytosol, stimulation of its DNA-dependent catalytic activity, and activation of innate immunity signaling via ISG activation.

Highlights

- RQC factors involved in disassembling collided ribosomes suppress the cGAS pathway
- Ribosomes interact with cGAS, stimulating its DNA-dependent activity
- cGAS preferentially interacts with collided ribosomes
- Ribosome collision leads to re-localization of cGAS to the cytosol and ISG activation

Wan et al., 2021, Molecular Cell *81*, 2808–2822
July 1, 2021 © 2021 The Author(s). Published by Elsevier Inc.
https://doi.org/10.1016/j.molcel.2021.05.018

CellPress
OPEN ACCESS

Molecular Cell

Article

Translation stress and collided ribosomes are co-activators of cGAS

Li Wan,[1] Szymon Juszkiewicz,[2] Daniel Blears,[1,3] Prashanth Kumar Bajpe,[1] Zhong Han,[1,3] Peter Faull,[4] Richard Mitter,[5] Aengus Stewart,[5] Ambrosius P. Snijders,[4] Ramanujan S. Hegde,[2] and Jesper Q. Svejstrup[1,3,6,*]
[1]Mechanisms of Transcription Laboratory, The Francis Crick Institute, 1 Midland Road, London NW1 1AT, UK
[2]MRC Laboratory of Molecular Biology, Francis Crick Avenue, Cambridge CB2 0QH, UK
[3]Department of Cellular and Molecular Medicine, Panum Institute, Blegdamsvej 3B, University of Copenhagen, 2200 Copenhagen, Denmark
[4]Protein Analysis and Proteomics Laboratory, The Francis Crick Institute, 1 Midland Road, London NW1 1AT, UK
[5]Bioinformatics and Biostatistics, The Francis Crick Institute, 1 Midland Road, London NW1 1AT, UK
[6]Lead contact
*Correspondence: jsvejstrup@sund.ku.dk
https://doi.org/10.1016/j.molcel.2021.05.018

SUMMARY

The cyclic GMP-AMP synthase-stimulator of interferon genes (cGAS-STING) pathway senses cytosolic DNA and induces interferon-stimulated genes (ISGs) to activate the innate immune system. Here, we report the unexpected discovery that cGAS also senses dysfunctional protein production. Purified ribosomes interact directly with cGAS and stimulate its DNA-dependent activity *in vitro*. Disruption of the ribosome-associated protein quality control (RQC) pathway, which detects and resolves ribosome collision during translation, results in cGAS-dependent ISG expression and causes re-localization of cGAS from the nucleus to the cytosol. Indeed, cGAS preferentially binds collided ribosomes *in vitro*, and orthogonal perturbations that result in elevated levels of collided ribosomes and RQC activation cause sub-cellular re-localization of cGAS and ribosome binding *in vivo* as well. Thus, translation stress potently increases DNA-dependent cGAS activation. These findings have implications for the inflammatory response to viral infection and tumorigenesis, both of which substantially reprogram cellular protein synthesis.

INTRODUCTION

In the innate immune system, pattern recognition receptors recognize both self and nonself features to activate signaling pathways that lead to the production of interferons (IFNs) and proinflammatory cytokines (Takeuchi and Akira, 2010; Brubaker et al., 2015; Tan et al., 2018). An important enzyme in this system, cyclic GMP-AMP synthase (cGAS), is activated by both cytosolic self-DNA and pathogen-derived DNA. Upon its activation, cGAS synthesizes 2′-3-cyclic GMP-AMP (cGAMP) (Ablasser et al., 2013a; Gao et al., 2013; Sun et al., 2013; Zhang et al., 2013), which functions as a second messenger that is bound by stimulator of interferon genes (STING). Binding of cGAMP leads to a conformational change and formation of STING oligomers (Ergun and Li, 2020). Subsequently, activated STING recruits and activates tank binding kinase 1 (TBK1), which in turn phosphorylates interferon regulatory factor 3 (IRF3). Phosphorylated IRF3 dimerizes and translocates into the nucleus to activate type I interferon (IFN) and interferon-stimulated genes (ISGs) (Chen et al., 2016; Kato et al., 2017; Ablasser and Chen, 2019; Hopfner and Hornung, 2020).

The cGAS-STING pathway plays a vital role in triggering the innate immune response to defend against DNA-containing pathogens. However, unlike the parallel RNA-sensing pathway where retinoic acid-inducible gene I (RIG-I) can distinguish between pathogen RNA and self-RNA by recognizing the 5′-triphosphate RNA ends generated by viral polymerases (Hornung et al., 2006; Pichlmair et al., 2006), cGAS lacks the ability to discriminate between pathogen-derived DNA and self-DNA (Kato et al., 2017; Hopfner and Hornung, 2020). Indeed, excessive activation of cGAS by self-DNA released from mitochondria or the nucleus may trigger autoimmunity and is associated with inflammatory diseases such as Aicardi-Goutières syndrome (AGS) (Ablasser et al., 2013b; Crow and Manel, 2015; Chen et al., 2016). Whether cGAS might rely on other intracellular cues that typify infection to increase the specificity of its activation is not known. In principle, coincident detection of two or more incompletely specific parameters could help restrict the cGAS response to pathogenic conditions and minimize inappropriate signaling.

One of the most common features of viral infection is the hijacking of the host protein synthesis machinery for large-scale virus production (Walsh and Mohr, 2011; Jaafar and Kieft, 2019). Not only do viruses have numerous mechanisms to bypass host attempts at inhibiting translation, but they also often employ host ribosomes in multiple unconventional ways to manipulate translation initiation, elongation, and termination (Firth and Brierley, 2012; Atkins et al., 2016; Jaafar and Kieft, 2019), potentially triggering translation stress.

Recent work has begun to elucidate the cellular pathways for detecting unusually slow or stalled ribosomes as a proxy for

 © 2021 The Author(s). Published by Elsevier Inc.
This is an open access article under the CC BY license (http://creativecommons.org/licenses/by/4.0/).

CellPress
OPEN ACCESS

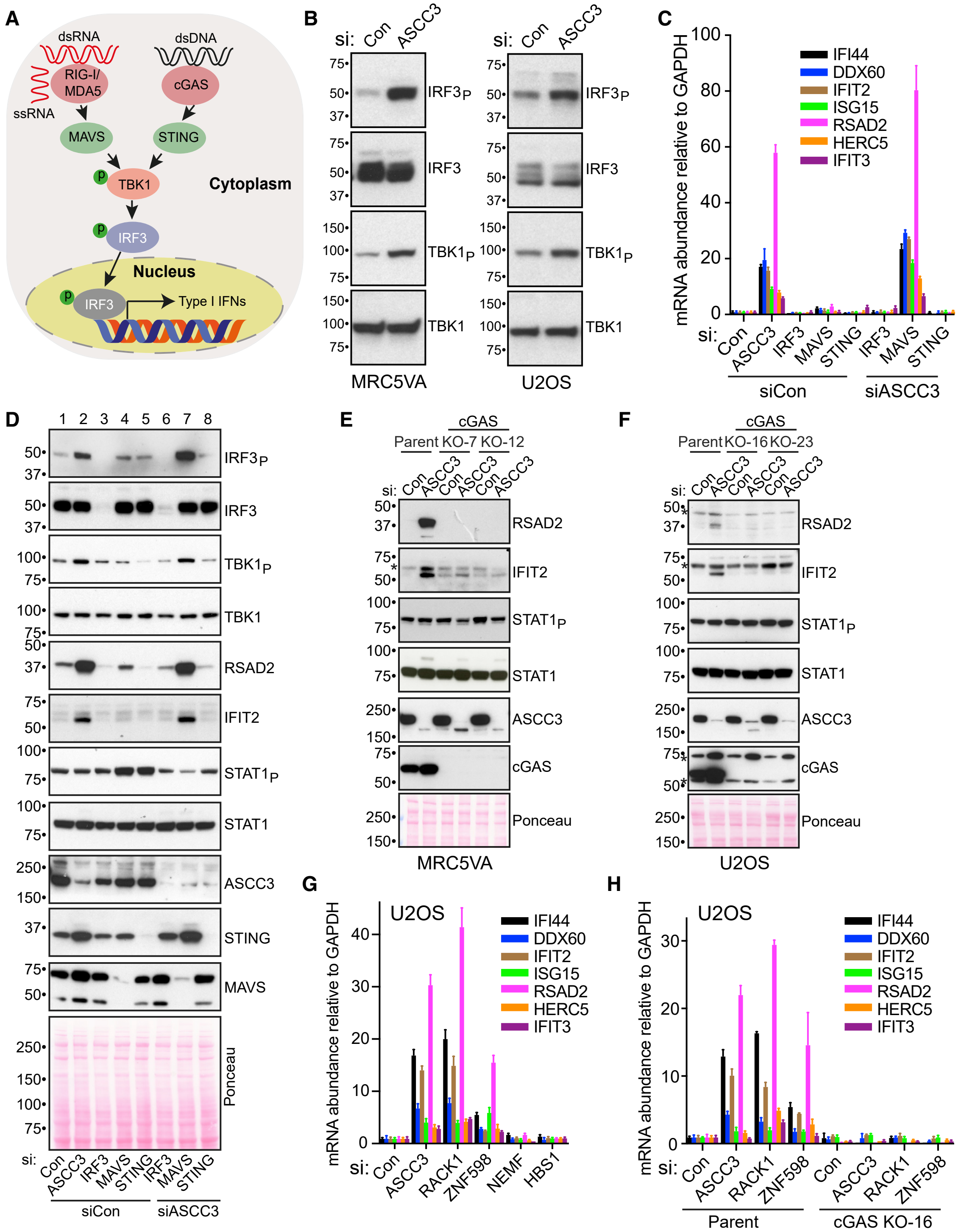

Figure 1. The cGAS-STING pathway is required for increased ISG expression in ASCC3-deficient cells

(A) Schematic of relevant innate immunity signaling pathways.

(B) Western blot analysis of IRF3 and TBK1, and their phosphorylated forms (IRF3ser396 or TBK1ser172), in cells depleted of ASCC3.

(C) qRT-PCR analysis of relative ISG expression in MRC5VA cells treated with siRNAs. Error bars represent standard deviation (SD) of three technical replicates and are representative of three biological replicates.

(D) Western blot analysis of IRF3p (ser396), IRF3, TBK1p (ser172), TBK1, RSAD2, IFIT2, STAT1p (tyr701), STAT1, ASCC3, STING, and MAVS in the same cells as in (C).

(legend continued on next page)

translation stress (Shoemaker and Green, 2012; Joazeiro, 2017; Collart and Weiss, 2020). When a ribosome stalls or slows substantially, the trailing closely spaced ribosomes may collide with it. Ribosome collisions are used by the cell as an indication of aberrant translation to initiate conserved pathways of mRNA decay and ribosome-associated protein quality control (RQC) (Brandman and Hegde, 2016; Joazeiro, 2019; Inada, 2020). In RQC, the ubiquitin ligase ZNF598 (Hel2 in yeast) specifically recognizes collided ribosomes (Ikeuchi et al., 2019; Juszkiewicz et al., 2018) and ubiquitylates the 40S subunit(s) to mark these translation complexes for downstream disassembly. The 40S protein RACK1 (Asc1 in yeast) is also required at this step, perhaps to stabilize the collided ribosome complex recognized by ZNF598 (Brandman et al., 2012; Letzring et al., 2013; Matsuo et al., 2017; Sundaramoorthy et al., 2017).

Our recent results show that the leading ribosome in a collided ribosome complex is disassembled by the conserved ASC-1 complex (ASCC) in an ATP-dependent reaction involving the helicase subunit ASCC3 (Juszkiewicz et al., 2020) Disassembly requires 40S ubiquitination by ZNF598, but not GTP-dependent factors such as the Pelo-Hbs1L ribosome rescue complex. Once the roadblock has been removed, the trailing ribosomes can elongate and become targets only if they themselves subsequently stall and incur collisions (Juszkiewicz et al., 2020). The homologs of ZNF598 and the ASCC appear to play an analogous role in yeast (Matsuo et al., 2020).

Here we provide evidence that translation stress triggers the innate immune response via the cGAS-STING pathway. The mechanism involves the ribosome acting as a co-activator of cGAS. Perturbations of early steps in the RQC pathway that lead to persistent ribosome collisions in cells result in cGAS accumulation in the cytosol and ISG activation. These results identify a previously unappreciated mechanism of cGAS activation that cells might exploit to broadcast translation stress via stimulation of ISG expression.

RESULTS

Ribosome quality control and cGAS-dependent activation of ISGs

Our previous findings, as well as those of others, indicated that ASCC3 deficiency leads to activation of ISGs (Li et al., 2013; Williamson et al., 2017), a characteristic of the innate immune response. At the time of those findings, ASCC3 was primarily known for its roles in transcriptional regulation (Jung et al., 2002) and DNA alkylation repair (Dango et al., 2011). Recently, however, ASCC3 has been shown to have a separate role in the cytosol where it participates in the RQC pathway, specifically the disassembly of collided ribosomes (Hashimoto et al., 2020; Juszkiewicz et al., 2020). The poorly understood connection between ASCC3, the RQC pathway, and gene expression motivated us to further investigate the mechanism of ISG activation.

We first used transient transcriptome sequencing (TT_{chem}-seq) (Gregersen et al., 2020) and quantitative reverse-transcription PCR (qRT-PCR) to show that ISG expression during ASCC3 deficiency was indeed due to increased transcription (Figures S1A–S1D). ASCC3 modulates ISG expression in an IRF3-dependent manner (Li et al., 2013), but whether ASCC3 acts as a transcription co-regulator in the nucleus or in the initial activation of IRF3 by phosphorylation in the cytosol was unknown (Figure 1A). We found that knockdown of ASCC3 affects IRF3 phosphorylation in two different ASCC3-depleted cell lines (Figures 1B and S1E). IRF3 is phosphorylated by TBK1, which is itself phosphorylated during innate immune signaling (Kato et al., 2017); we observed elevated phosphorylation of TBK1 as well (Figures 1B and S1E). This shows that activation of ISGs in ASCC3-deficient cells is not due to a role as, for example, a transcription repressor, but that it originates in the activation of the TBK1-IRF3 pathway in the cytosol.

ASCC3 is a component of the ASCC, along with ASCC1, ASCC2, and TRIP4 (Jung et al., 2002), but small interfering RNA (siRNA) knockdown of the other ASCC subunits resulted in only modest increases in IRF3 and TBK1 phosphorylation and a slight upregulation of ISG expression (Figures S1F and S1G), suggesting that ASCC3 is the key functional subunit of the ASCC. This parallels the effect of individual subunit knockdowns on ribosome stalling (Hashimoto et al., 2020; Juszkiewicz et al., 2020), suggesting that the two might be linked. ASCC3 was required for the stability of the other ASCC subunits, while their depletion had limited or no effect (Figure S1F). Therefore, we focused on the ASCC3 subunit in our further studies.

We next investigated which innate immune signaling pathway (Figure 1A) is responsible for the increase in ISG expression observed during ASCC3 deficiency. Double-knockdown experiments indicated that knockdown of STING, but not mitochondrial antiviral-signaling protein (MAVS), completely abrogated phosphorylation of both IRF3 and TBK1, as well as the increased ISG expression in ASCC3-depleted cells (Figures 1C and 1D, compare lane 2 with lanes 7 and 8; see also Figures S2A and S2B). *CGAS* knockouts (KOs) were also generated in two different cell types (Figures S3A and S3B). Experiments in these cells showed that the induction of ISG expression observed upon ASCC3 depletion was cGAS dependent (Figures 1E, 1F, S2E, S2F, S8E, and S8F). Similar results were observed by using cGAS siRNAs (Figure S2D). Phosphorylation of IRF3 and TBK1 was also cGAS dependent (Figure S2C), and the level of cGAMP was increased in cells in which ASCC3 was knocked down (Figure S1H). We also investigated the phosphorylation of STAT1, which functions alongside IRF3 and the IFN signaling pathway (McNab et al., 2015). Interestingly, ASCC3 depletion had little

(E) Western blot analysis of RSAD2, IFIT2, STAT1p (tyr701), STAT1, ASCC3, and cGAS in parental MRC5VA and two different *CGAS* knockout cell lines (KO-7 and -12) after ASCC3 knockdown. Asterisks denote non-specific bands.
(F) As in (E), but in U2OS cells.
(G) qRT-PCR analysis of relative ISG expression in U2OS cells transfected with the indicated siRNAs. Error bars represent SD of three technical replicates and are representative of three biological replicates.
(H) As in (G), but also using U2OS *CGAS* KO-16 cells.
See also Figures S1–S4.

effect on STAT1 phosphorylation (Figures 1D–1F). Moreover, we failed to detect significant nascent RNA reads for the IFN-α/β genes in our TT_{chem}-seq data in both control cells and those depleted of ASCC3.

Taken together, these results show that the cGAS-STING pathway is responsible for the induction of ISG expression in ASCC3-deficient cells.

cGAS-STING-dependent activation of ISGs in cells deficient in ribosome quality control

Previous studies suggested that ASCC3 is involved in multiple distinct cellular processes, such as transcriptional activation, DNA alkylation repair, transcription repression after UV irradiation, and RQC (Jung et al., 2002; Dango et al., 2011; Brickner et al., 2017; Matsuo et al., 2017; Williamson et al., 2017). Because the cGAS pathway is connected with the DNA damage response and ASCC3 is involved in DNA alkylation repair (Dango et al., 2011; Li and Chen, 2018), it seemed plausible that a DNA damage-associated process might play a role in cGAS-dependent ISG expression upon ASCC3 depletion. However, several findings argue against this possibility. First, ASCC2 is crucial for the role of ASCC in DNA repair (Brickner et al., 2017), but it has little or no effect on ISG activation (see above), suggesting that cGAS activation is unrelated to DNA repair. Second, ASCC regulates DNA alkylation repair only in certain cell types, but not in U2OS cells (Dango et al., 2011), which were used in many of our experiments. Third, neither U2OS nor MRC5VA cells depleted of ASCC3 showed γH2AX foci, a hallmark of DNA damage (Figures S3C and S3D). Finally, micronuclei, a potential source of DNA to stimulate cGAS activation (Bartsch et al., 2017; Harding et al., 2017; Mackenzie et al., 2017), did not increase markedly upon ASCC3 depletion either (Figure S3E). Together, these data indicate that ASCC3-associated DNA damage repair is unlikely to play a role in regulating the cGAS-STING pathway. Therefore, we set out to address which other ASCC3-associated biological process contributes to innate immune suppression.

In the hope that an answer might lie in the interactors of the ASCC, we used cells expressing individual, tagged ASCC subunits for stable isotope labeling by amino acids in cell culture (SILAC)-based quantitative mass spectrometry (Figures S4A–S4C; Table S1). As expected, the four subunits of the ASCC were repeatedly identified with high confidence from these experiments. Gratifyingly, specific proteins of the protein translation machinery, such as the eIF3 complex and the small ribosomal subunit proteins, were identified as well (Figures S4B and S4D). This matches our recent finding that the ASCC dissociates the leading ribosome into subunits upon ribosome collision (Juszkiewicz et al., 2020) and might suggest that it can remain associated with the 40S after separation from the 60S. This experiment also provides a link between ASCC and the ribosome that might help explain why ASCC deficiency leads to cGAS-dependent ISG activation.

One consequence of ASCC3 deficiency is an inability to promptly resolve collided ribosomes via the RQC pathway. The collisions persist in ASCC3-deficient cells, but eventually read-through stall-inducing sequences such as poly(A) as measured using a dual-fluorescence translation reporter (Juszkiewicz and Hegde, 2017; Juszkiewicz et al., 2020) (Figures S4E–S4H). Other proteins in the early RQC pathway, such as the E3 ligase ZNF598 and 40S protein RACK1, similarly show collision persistence, correlating with increased reporter read-through. Tellingly, knockdown of each of these RQC factors induced ISG expression in a cGAS-dependent manner as well (Figures 1G and 1H). Because neither ZNF598 nor RACK1 is involved in alkylation repair, these results provide additional evidence that cGAS activation is caused by RQC deficiency rather than byproducts of deficient DNA repair.

We note that neither increased ISG expression nor increased read-through at translation stall sites was observed in cells depleted of HBS1 (Hbs1L) or NEMF (Figure 1G; Figures S4F and S4G), which function as a different branch of RQC or act later after ribosome subunit dissociation (Brandman and Hegde, 2016; Joazeiro, 2019; Juszkiewicz et al., 2020). Simultaneous knockdown of HBS1 and its homolog GTPBP2 (Ishimura et al., 2014), or its cofactor PELOTA (Brandman and Hegde, 2016), did not increase ISG expression either (Figures S2I and S2J), further supporting the idea that only the RQC factors that dissociate collided ribosomes regulate the cGAS-STING pathway.

These data indicate that perturbation of the early RQC pathway, which leads to the persistence of collided ribosomes, activates ISGs through the cGAS-STING pathway.

cGAS interacts directly with ribosomes

The data so far suggest an unexpected connection between cGAS activation and the RQC. In the hope of uncovering the protein interactions responsible, we now used affinity purification (AP) and SILAC-based quantitative mass spectrometry to characterize the cGAS interactome (Figure 2A). Ribosomal proteins and histones were among the most convincing interacting factors (Figures 2B and 2C; see also Table S2). cGAS is detected in the nucleus (Gekara and Jiang, 2019) and has been shown to have higher affinity for nucleosomes than for naked DNA (Zierhut et al., 2019), providing a likely explanation for why histones were among the interactors. Although we cannot rule out that the interaction between cGAS and ribosomes might at least partially occur post-lysis because of the breakdown of the nuclear membrane, the relevance of the interacting ribosomal proteins was supported by the results on ISG activation described above. We note that ribosomal proteins have previously been identified in a cGAS interactome but were considered non-specific interactors (Lum et al., 2018). Although ribosomes are indeed frequent contaminants in AP-mass spectrometry experiments due to their high abundance, the degree of enrichment with cGAS was exceptional and comparable with that of histones and even cGAS itself (Figure 2C). We therefore examined the relationship in co-immunoprecipitation experiments and confirmed that cGAS interacts robustly with ribosomal proteins and also histone H3 (Figure 2D).

The recovery of nearly all 40S and 60S ribosomal proteins at comparable levels suggested that cGAS interacts with intact ribosomes rather than individual constituent proteins. Consistent with this idea, we observed that, strikingly, almost all cGAS detected in the cytosol of U2OS cells co-fractionated with ribosomes upon sucrose gradient sedimentation (Figure 3A).

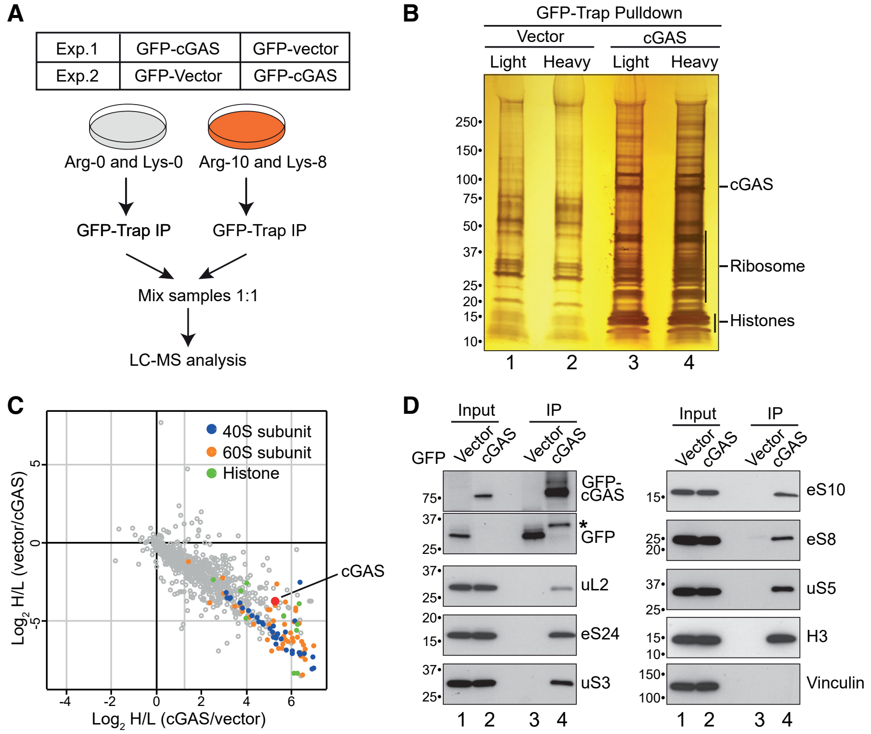

Figure 2. The cGAS interactome

(A) Strategy for SILAC-based quantitative mass spectrometry.

(B) Silver staining showing GFP-associated factors (Vector [control]) and GFP-cGAS-associated factors (cGAS), respectively.

(C) Scatterplots of Log2 SILAC ratios for the cGAS interactome. Small ribosomal proteins are marked in blue, large ribosomal proteins in orange, and histone proteins in green.

(D) Validation by immunoprecipitation (IP)-western blotting with the indicated antibodies. The two upper panels on the left are from same the anti-GFP blot. Asterisk denotes a likely GFP-cGAS degradation product.

Furthermore, highly purified recombinant His-tagged cGAS immobilized on Ni-NTA efficiently pulled down purified human ribosomes, whereas Ni-NTA agarose alone, or His-tagged hPrimpol 1 (a control protein that also binds DNA) (Wan et al., 2013), did not (Figures 3B and S5A). To rule out the possibility that cGAS interacts with the ribosomes via (contaminating) DNA, we also treated the ribosomes extensively with DNase during purification (Figure S6C). We then pre-incubated purified, recombinant cGAS with the purified, DNase-treated ribosomes and fractionated the reaction by sucrose gradient sedimentation. When fractionated alone, cGAS remained in the slowly sedimenting, low-molecular-weight fractions, as expected (Figure 3C). However, when pre-mixed, cGAS and DNase-treated ribosomes co-sedimented in high-molecular-weight fractions (Figure 3C), strongly indicating a direct interaction.

To identify which region of cGAS interacts with ribosomes, cGAS deletion mutants (Figure S5B) were generated and expressed in cells for analysis in ribosome co-fractionation experiments (Figure S5D, left panel: truncation mutation). Interestingly, although an N-terminal fragment containing the first 382 of the 522 amino acids (aa) comprising cGAS ($cGAS_{1–382}$) interacted efficiently with ribosomes, $cGAS_{1–341}$ did not. Moreover, co-migration was somewhat compromised in cGAS harboring N-terminal deletions ($cGAS_{161–522}$ to $cGAS_{294–522}$) (Figure S5D, left panel: truncation mutation), together suggesting that a domain located between aa 341 and 382 of cGAS is primarily responsible for the interaction, but with the N terminus contributing as well.

To further delineate which aa between 341 and 382 might be responsible for the interaction, we generated a series of cGAS point mutants in which lysine (K) and/or arginine (R) residues primarily in this region of cGAS were replaced with alanine (A) or glutamic acid (E) (Figure S5C). These experiments indicated that K347, R349, K350, and R353 of cGAS are important for the interaction. However, replacement of all K and R residues between aa 347 and 353 with A still showed residual ribosome interaction (Figure S5D, right panel). This may be explained by the observation that the disordered N-terminal domain of cGAS (Tao et al., 2017; Du and Chen, 2018; Barnett et al., 2019) also plays a role (summarized in Figure S5B, left). Indeed, the binding characteristics of RBM(K/R-A) were similar to that of the $cGAS_{1–341}$ fragment.

Together, the variety of experimental approaches used above indicates that cGAS binds directly and strongly to the ribosome.

Ribosomes stimulate the catalytic activity of cGAS

The ability of cGAS to bind ribosomes led us to examine whether such an association affects the DNA-stimulated catalytic activity of cGAS. For this purpose, an *in vitro* assay was established, in which the ability of highly purified, recombinant human cGAS to produce cGAMP was measured by thin-layer chromatography (Civril et al., 2013; Kranzusch et al., 2013; Zhou et al., 2018). As expected, cGAS synthesizes cGAMP only in the presence of DNA (Figures S6A and S6B). To examine whether ribosomes affect cGAS activity, we pre-incubated varying concentrations of cGAS with purified ribosomes and then added saturating DNA to activate cGAMP synthesis (Figure 4A). At low to moderate cGAS concentrations, ribosomes robustly stimulated cGAS activation (Figure 4A, lanes 3–10; see also see inset), giving rise to a ~3- to ~8-fold stimulation at 150–300 nM. At the saturating cGAS concentrations often used in such assays, ribosomes had no obvious effect (Figure 4B) (Civril et al., 2013; Kranzusch et al., 2013; Zhou et al., 2018). Given that we used saturating DNA in the reactions (Figure 4A), it seemed unlikely that any DNA potentially contaminating the ribosome fraction was stimulating cGAS activation. Nevertheless, we also performed experiments with DNase-treated ribosomes and observed that these also dramatically stimulate cGAS activity. Notably, neither DNase-treated nor

CellPress
OPEN ACCESS

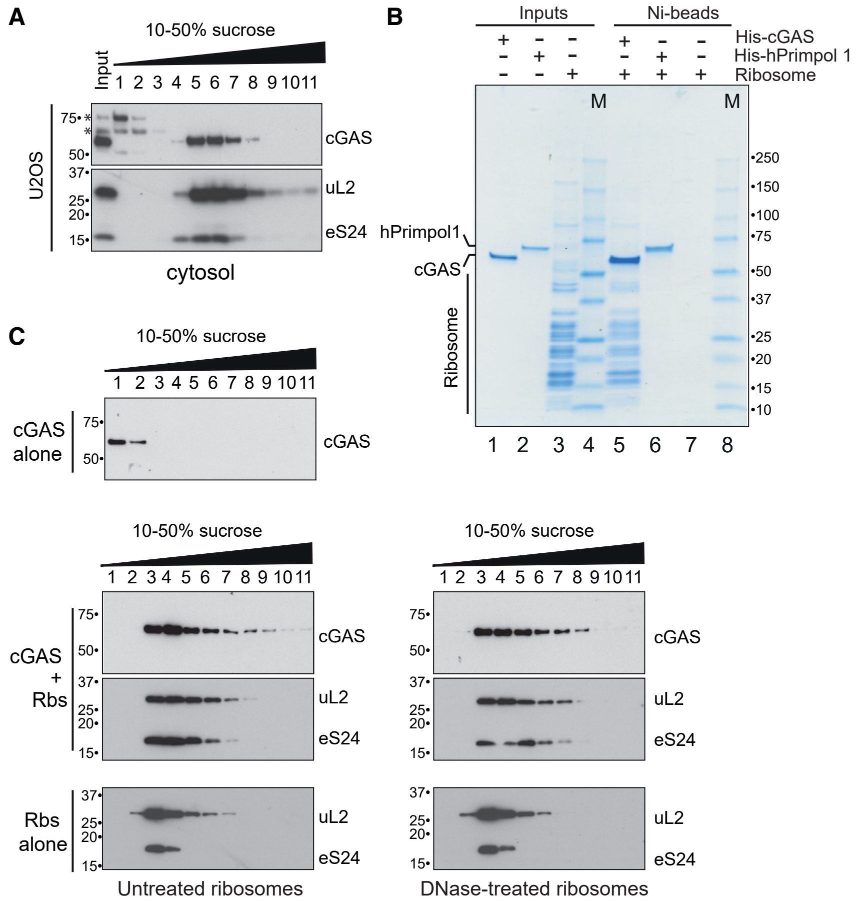

Figure 3. Evidence for a direct cGAS-ribosome interaction

(A) Cytosol from U2OS cells was separated by sucrose gradient sedimentation, and fractions were immunoblotted for cGAS and representative ribosome subunits (ul2 and eS24). Asterisks denote non-specific bands.

(B) Purified ribosomes were incubated with Ni-NTA agarose, Ni-NTA agarose with immobilized human recombinant cGAS-8his, or hPrimpol1-8his (control). After washing, the eluate was analyzed by SDS-PAGE and Coomassie staining.

(C) Western blot analysis of cGAS, ribosomes (both untreated and DNase-treated ribosomes), or cGAS-ribosome complex, separated by sucrose gradient sedimentation.

See also Figure S5.

untreated ribosomes were able to activate cGAS in the absence of DNA (Figures 4C and 4D), indicating that ribosomes act as co-activators of cGAS. The ribosome sample was also incubated at 95°C for 5 min and then allowed to slowly cool to room temperature. This should allow nucleic acid structures, but not the ribosomes, to re-form. These heat-treated ribosomes were unable to stimulate cGAS activity (Figures 4E and 4F), further supporting the conclusion that contaminating nucleic acids were not involved and indicating that intact ribosomes are required for co-activation of cGAS.

Together, these experiments indicate that ribosomes not only associate with cGAS but also potently stimulate its DNA-dependent catalytic activity. We note that the concentration of cGAS (150–300 nM) where robust activation was observed is in its physiological concentration range (i.e., 10–500 nM (Andreeva et al., 2017; Du and Chen, 2018), rather than supraphysiological, as often previously used *in vitro* (1–2 μM cGAS is typically used to measure DNA-dependent activity) (Civril et al., 2013; Kranzusch et al., 2013; Zhou et al., 2018). This indicates that ribosomes act as co-activators of cGAS.

ASCC3-mediated RQC regulates cGAS in a DNA-dependent manner

Previous biochemical and structural studies revealed several cGAS features that are important for its DNA-dependent activation (Civril et al., 2013; Gao et al., 2013; Kranzusch et al., 2013; Zhang et al., 2014; Zhou et al., 2018). Our experiments *in vitro* showed that ribosomes markedly stimulate cGAS DNA-dependent activity, but also that ribosomes are unable to activate cGAS in the absence of DNA (Figures 4C and 4D). We also described above that K347, R349, K350, and R353 of cGAS are important for its interaction with ribosomes; these residues are in cGAS DNA binding site B (Figure S5C). Not unexpectedly, therefore, cGAS ribosome-binding mutants in which these residues were mutated lost the ability to respond to exogenous DNA *in vivo* (Figures S7A–S7D). Mutation of either the cGAS active site (E225A and D227A), DNA binding region A (K411A), or the Zinc-Ribbon (C396A and ΔZinc-ribbon) also abrogated DNA-stimulated ISG expression (Figures S7E and S7F). More importantly, in cells treated with ASCC3 siRNA, activation of ISG expression was not observed in any of these mutants either (Figures S7G and S7H), indicating an overlap in the cGAS features required for the two mechanisms of ISG activation.

During the potent activation of innate immunity signaling observed upon infection, DNA from the invading microorganism is the likely source of cGAS activation, but where might the DNA required to co-stimulate cGAS activity upon the experimental perturbation of the RQC pathway come from? Through immunofluorescence experiments using anti-dsDNA antibody, we detected cytosolic DNA in both control cells and those transfected with ASCC3 siRNA, and cGAS colocalized with cytosolic DNA, particularly in cells depleted of ASCC3 (Figure S7I). Recent evidence suggests that mitochondrial DNA may represent an important source of immuno-stimulatory DNA for cGAS activation during various stresses (West et al., 2015). We therefore set out to test whether DNA released from mitochondria might affect ASCC3-mediated cGAS activation. The activation of ISG gene expression in cells depleted of ASCC3 was indeed markedly reduced when mitochondrial DNA was depleted using a low concentration of ethidium

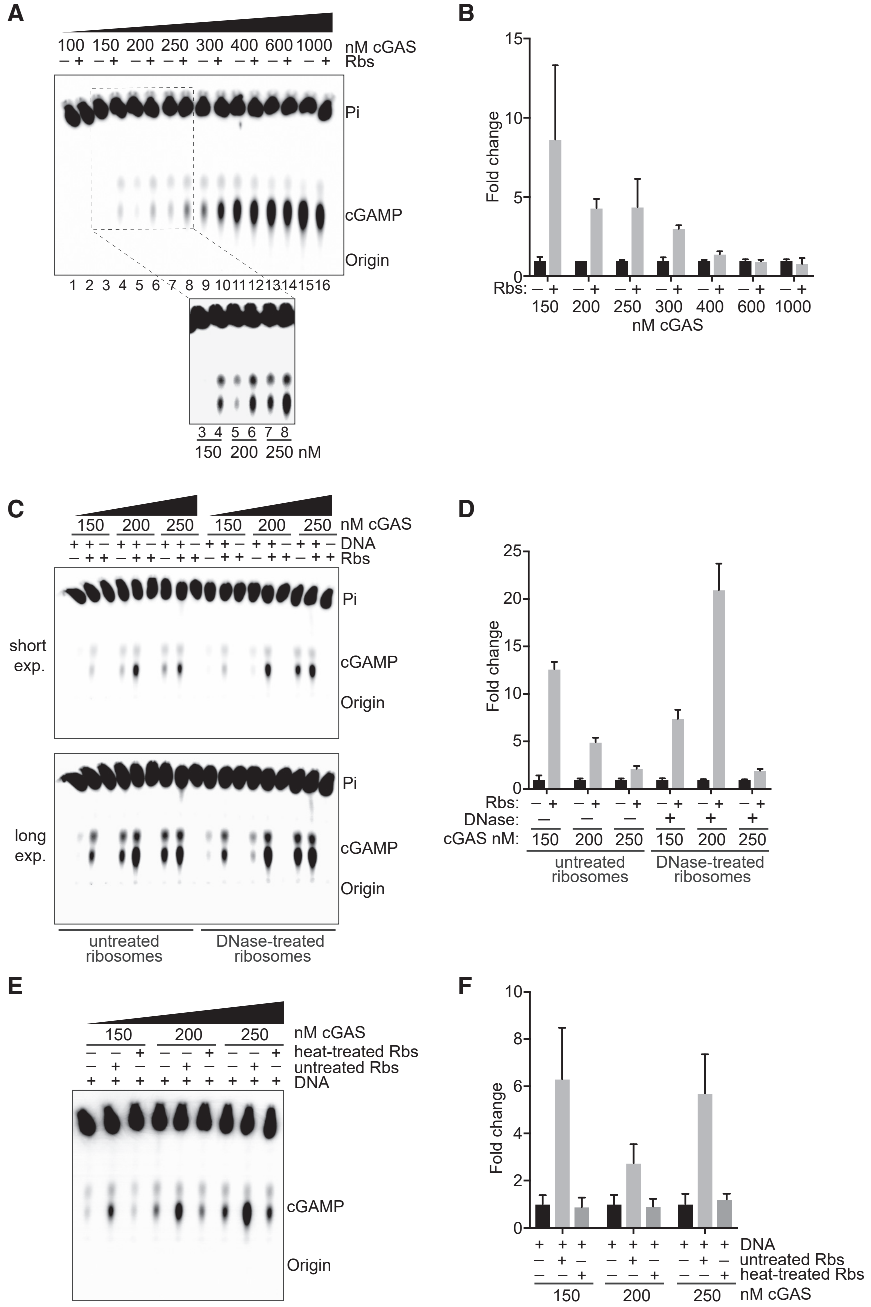

Figure 4. Ribosomes stimulate DNA-dependent cGAS activity *in vitro*

(A) Autoradiograph of cGAS-mediated cGAMP synthesis in the presence of different concentrations of cGAS with or without ribosomes; all are in the presence of 1 μg herring testis DNA, which is saturating (see Figures S6A and S6B).

(B) Quantification of data in (A) by Fiji. Error bars indicate SD of duplicate replicates.

(C) As in (A), but with untreated ribosomes or DNase-treated ribosomes.

(D) Quantification of the data from (C), as in (B).

(E) As in (A), but with untreated or heat-treated ribosomes.

(F) Quantification of the data from (E), as in (B).

See also Figures S6 and S7.

bromide to inhibit its synthesis (Nass, 1972) (Figures S7J and S7K). Of note, such DNA depletion even reduced the low-level ISG expression observed in control cells (Figure S7J, "Si controls"), suggesting that "background" levels of mitochondrial DNA in the cytosol might sustain basal ISG expression via the cGAS-STING pathway.

cGAS preferentially recognizes collided ribosomes

Given that RQC deficiency results in ISG activation (cf. Figure 1), and that cGAS interacts with ribosomes (Figures 2 and 3), we now hypothesized that the signal causing activation of cGAS in ASCC3-, ZNF598-, or RACK1-depleted cells is unresolved, collided ribosomes (Juszkiewicz et al., 2018, 2020; Hashimoto et al., 2020). To test this, we first generated and purified collided ribosomes (and non-translating ribosome controls) from rabbit reticulocyte lysates as previously described (Juszkiewicz et al., 2018) (Figure 5A). To compare binding, we incubated cGAS with varying ratios of collided and control ribosomes. Upon sucrose gradient fractionation of the binding reaction, cGAS preferentially migrated with collided ribosomes, even when the non-translating 80S ribosome particles were present at a 3-fold excess (Figure 5B). Thus, cGAS has higher affinity for collided ribosomes. Given that polysomes and collided ribosomes cannot be distinguished in this experiment, it was formally possible that cGAS simply prefers polysomes (and not collided ribosomes) over mono-ribosomes or the non-translating 80S particle, but this was not the case. Rather, in the absence of collided ribosomes, cGAS actually appears to prefer monosomes over translating ribosomes (polysomes) (Figure 5C).

Together, these data indicate that cGAS binds preferentially to collided ribosomes, whereas it has less affinity for monosomes and binds very poorly to translating polysomes.

cGAS accumulates in the cytosol upon translation stress

The data above suggest that cGAS recognizes collided ribosomes in RQC-deficient cells and then activates the TBK1-IRF3 pathway to upregulate inflammatory genes. Previous data had suggested that cGAS is detected in the nucleus (Orzalli et al., 2015; Yang et al., 2017; Volkman et al., 2019), prompting us to check the subcellular localization of cGAS before and after exposure to translation stress. The sub-cellular localization of

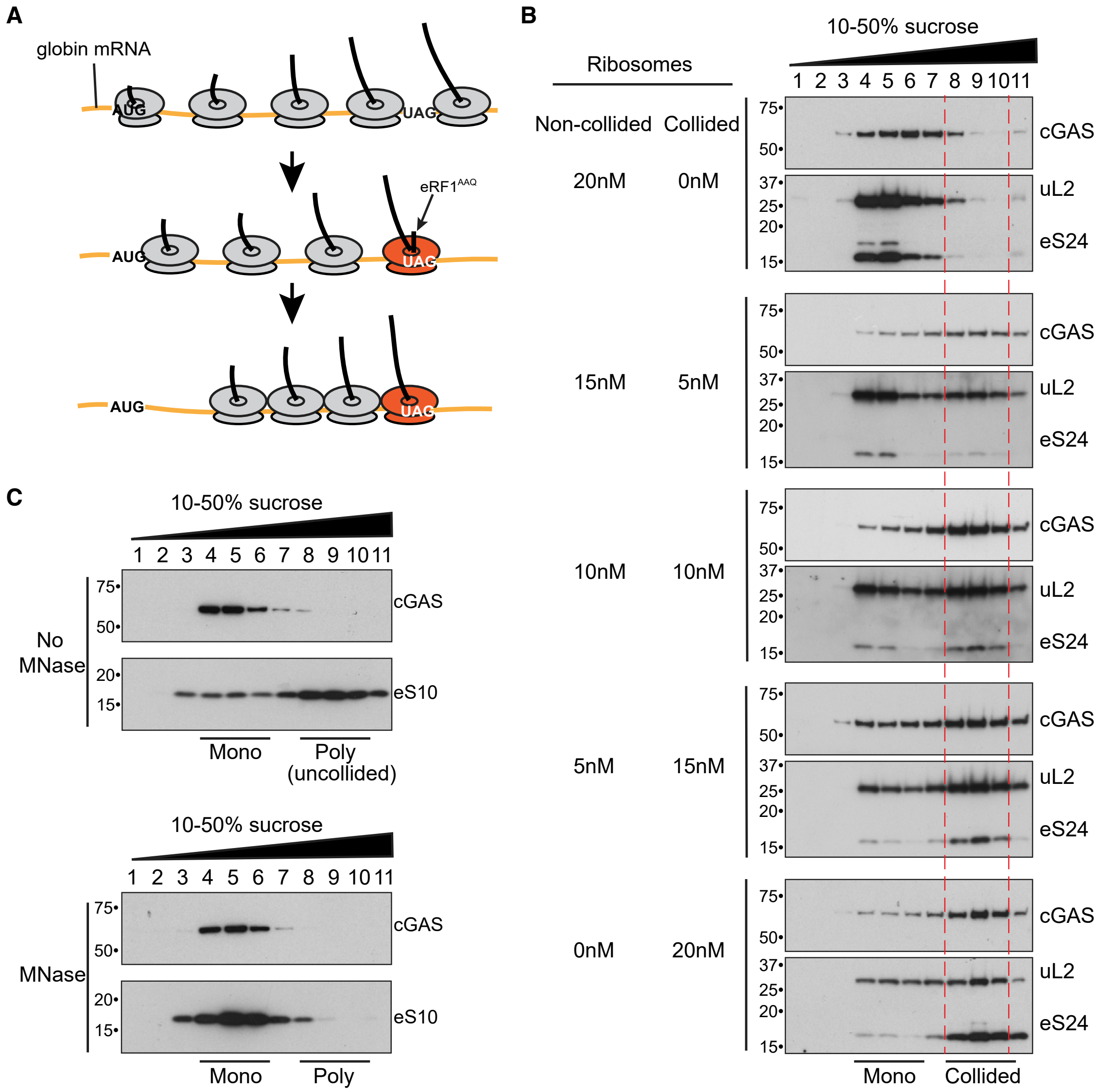

Figure 5. cGAS preferentially interacts with collided ribosomes

(A) Strategy to generate collided ribosomes using an *in vitro* translation reaction in rabbit reticulocyte lysate (Juszkiewicz et al., 2018).

(B) cGAS was incubated with a mixture of collided and non-collided ribosomes at different ratios, separated by 10%–50% sucrose gradient fractionation, and then analyzed by western blotting. Collided ribosomes (fractions 8, 9, and 10) are indicated by dashed lines.

(C) Western blot analysis of MRC5VA cytosol fractionated by sucrose gradient sedimentation, with or without prior incubation with micrococcal nuclease (MNase) to digest polysomes (poly) to monosomes (mono).

cGAS has been a matter of some debate (Gekara and Jiang, 2019), but in apparent agreement with data reported by others (Gentili et al., 2019; Volkman et al., 2019), we observed that GFP-cGAS is predominantly a nuclear protein in U2OS cells under normal conditions, while the ribosome (here visualized by the ribosomal protein eS8) was predominantly cytosolic, as expected (Figure 6A, siCon). Remarkably, however, in cells depleted of ASCC3, ZNF598, or RACK1 where cGAS-dependent stimulation of ISG expression is observed, cGAS was not only detected almost exclusively in the cytosol but also co-localized with ribosomes (Figure 6A; quantification in Figure S8A). This supports the idea that cGAS accumulates with ribosomes in the cytosol upon translation stress, and that cytosolic cGAS localization can be used as a proxy for its activation.

To investigate whether cGAS localization is likewise altered in response to other kinds of translation stress that lead to an increase in collided ribosomes, we used two different inhibitors of translation elongation, anisomycin and emetine, in wild-type (WT) cells. At a low concentration of these inhibitors, some ribosomes stall so that uninhibited ribosomes catch up and cause collision, whereas at a high fully inhibitory concentration, all ribosomes stall and thus do not collide (Simms et al., 2017; Juszkiewicz et al., 2018). Strikingly, low concentrations of elongation inhibitors did indeed lead to accumulation of cGAS in the

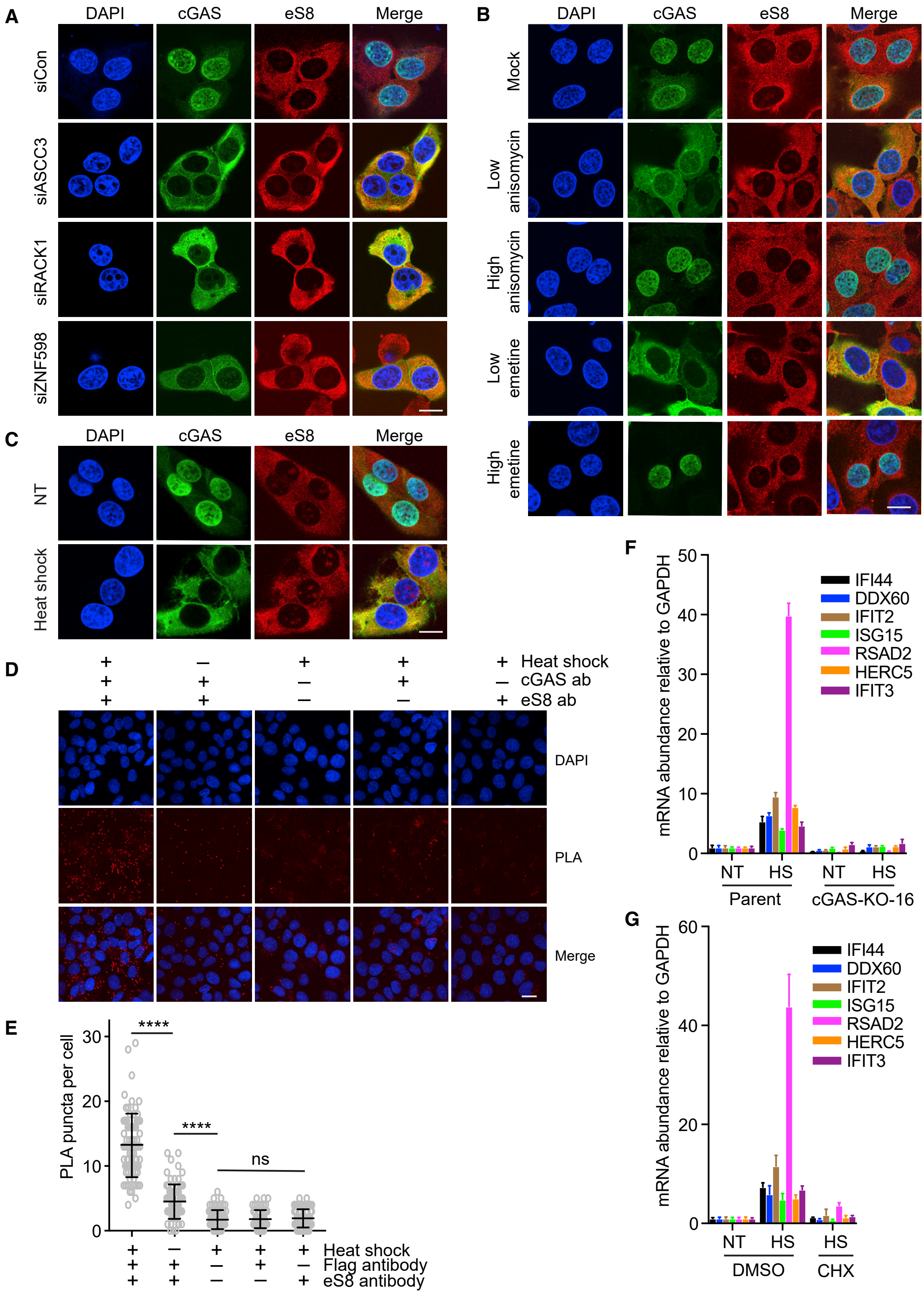

(legend on next page)

CellPress
OPEN ACCESS

cytosol, while treatment with a high, fully translation-inhibitory concentration did not (Figures 6B and S8B).

Previous studies have shown that heat shock can also induce ribosome stalling during translation elongation (Liu et al., 2013; Shalgi et al., 2013; Merret et al., 2015). We surmised that such stalling might therefore also induce a change in the subcellular localization of cGAS. Indeed, cGAS was predominantly located to the cytosol ~6–7 h after acute heat shock treatment (43°C for 45 min) (Figures 6C and S8C). This localization change was completely inhibited by pretreatment with cycloheximide, indicating that the change in subcellular localization of cGAS during heat shock requires active translation (Figure S8D). Indeed, consistent with the observation that cGAS colocalizes with ribosomes after translation stress (Figures 6A–6C), *in situ* proximity ligation assay (PLA) showed that the signal for cGAS-ribosome proximity was markedly increased after heat shock treatment in a translation-dependent manner (Figures 6D and 6E). Moreover, WT, but not cGAS KO, cells showed heat shock-induced ISG transcription (Figure 6F), and this was translation dependent (Figure 6G).

Together, these results indicate that cGAS interacts with ribosomes *in vivo* and, importantly, that it accumulates in the cytosol upon different types of translation stress.

DISCUSSION

Together, the experiments described here support the surprising conclusion that problems during protein synthesis contribute to cGAS activation. cGAS is a central player in the innate immune response, which has so far been thought to work exclusively by sensing cytosolic DNA. We show that cGAS binds strongly and directly to ribosomes, with a clear preference for collided ribosomes, which often accumulate during translation stress. Ribosome binding leads to cGAS activation, both *in vitro* and inside cells, with induction of cellular translation stress resulting in the cytosolic accumulation of cGAS and activation of downstream signaling pathways to activate immune response genes. Intriguingly, such activation remains DNA-dependent.

Several questions arise from the observation that ribosomes co-activate cGAS, such as: why would a mechanism of coincidence activation have evolved? How does the activation of cGAS by translation stress relate to the activation by DNA in the cytoplasm? What is the relevance of these findings to the normal function of cGAS in the detection of invading microorganisms? Although the answers to most of these questions await further investigation, tentative answers to some may already be available from previous work.

The cGAS-STING pathway senses viral and bacterial infection, although it can also be triggered by non-infectious cellular stresses that elicit the release of DNA into the cytosol (Ablasser and Chen, 2019; Hopfner and Hornung, 2020). This wide role is facilitated by cGAS's ability to interact with double-stranded DNA in a sequence- and species-independent manner. A non-specific sensing mechanism of this kind requires additional control mechanisms to ensure that erroneous activation of the innate immune response does not occur (Ablasser and Chen, 2019). The cGAS-STING pathway may normally be suppressed by cGAS sequestration at the cell plasma membrane or more commonly in the nucleus (Gekara and Jiang, 2019). The nuclear localization of cGAS in many cell types may be because of its tight binding to nucleosomes (Boyer et al., 2020; Kujirai et al., 2020; Michalski et al., 2020; Pathare et al., 2020; Zhao et al., 2020). Similarly, collided ribosomes, to which we show cGAS binds very strongly, may shift the balance toward the cytosol during translation stress. Storing a DNA-sensing protein in the nucleus with all the DNA seems counter-intuitive. Even with nucleosomal packaging repressing its activity (Xie and Patel, 2020), extensive regions of nucleosome-free DNA, either in the chromosomes themselves or as excised fragments during DNA repair, are constantly generated, which would be expected to activate nuclear cGAS. An intriguing possibility arising from our work is that cGAS is kept inactive in the nucleus not only by binding to nucleosomes but also because protein synthesis does not take place there. In this working model, cGAS requires both excessive free DNA and ribosome binding in the cytoplasm for full activation. We found that when purified cGAS binds ribosomes, it is much more potently activated by DNA than without ribosomes. Indeed, at physiological cGAS concentrations, cGAS activity in the presence of DNA was barely detectable but could be induced up to 15-fold by the addition of ribosomes. In a coincidence sensing/co-activation model, potent activation of cGAS under physiological conditions would occur only when both increased cytosolic DNA *and* translation stress are sensed, such as during virus infection. Future experiments will be focused on addressing this intriguing possibility, but cGAS activation and the ISG expression levels observed in response to translation stress alone are much less forceful than after virus invasion or DNA transfection (the latter of doubtful physiological relevance). Our results suggest that DNA released by mitochondria might potentially provide a background reservoir of cytosolic

Figure 6. Conditions that result in collided ribosomes induce cytosolic localization of cGAS

(A) U2OS cells stably expressing GFP-cGAS were transfected with the indicated siRNA. Cells were fixed, stained with eS8 antibody or with DAPI, and imaged by confocal fluorescence microscopy. Scale bar: 10 μm.

(B) As in (A), but after treatment with the indicated drug regimens.

(C) As in (A) and (B), but after acute heat shock treatment. Quantification of Figures S8A–S8C.

(D) Analysis of the interaction between cGAS and ribosomes using the *in situ* proximity ligation assay (PLA) before and after heat shock in U2OS cGAS KO cells stably expressing FLAG-hemagglutinin (HA)-tagged GAS. Cells were fixed, incubated with the indicated antibodies, and visualized according to instruction of Dulink *In Situ* Kit. The PLA signal was detected by confocal fluorescence microscopy. Scale bar: 10 μm.

(E) Quantitative analysis of (D). Two-tailed t test, ****$p < 0.0001$. Error bars represent SD of puncta per cell from 80 cells per condition.

(F) qRT-PCR analysis of relative ISG expression in U2OS cells treated with heat shock. Error bars represent SD of three technical replicates and are representative of three biological replicates.

(G) As in (F), but cells are also treated with translation inhibitor cycloheximide (CHX).

ns, no significant. See also Figure S8 for quantification.

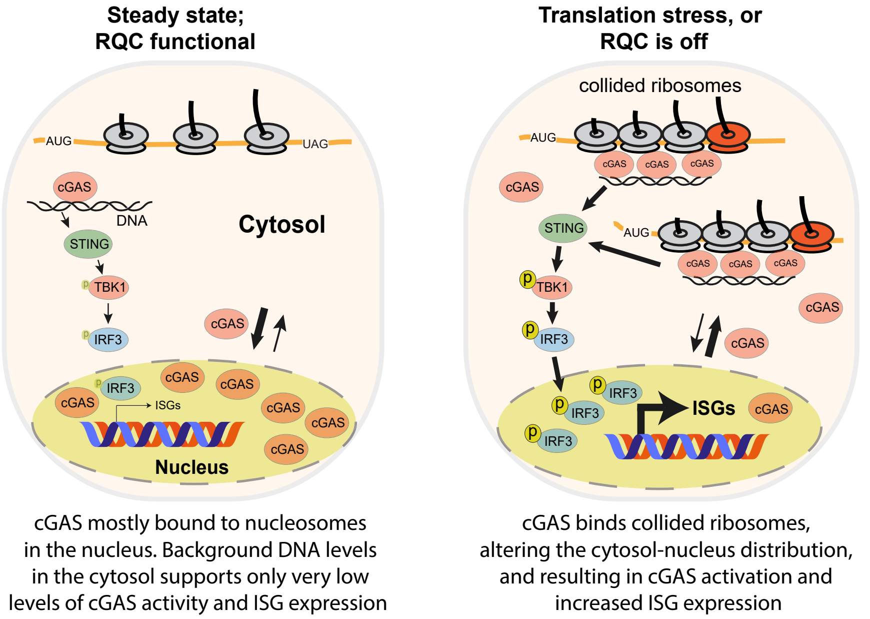

Figure 7. Working model for the cGAS response to translation stress

(A) In steady state or when the RQC is functional, cGAS predominantly binds to nucleosomes in the nucleus. Background DNA levels in the cytosol support very low levels of cGAS activity and background ISG expression.

(B) During translation stress or when the RQC is off, cGAS binds to collided ribosomes, which alters the cytosol-nucleus distribution and results in cGAS activation and increased ISG expression.

DNA to co-activate with translation stress, but other sources of DNA for co-activation cannot be excluded.

It is worth emphasizing that our model (Figure 7) for how translation stress contributes to cGAS activation is not mutually exclusive with other modes of cGAS regulation, such as phase separation, cGAS post-translational modification, and regulation of the cGAS-STING response by DNA length and nucleases, for example (Ablasser and Chen, 2019). We also note that the precise mechanism by which ribosomes co-stimulate cGAS catalytic activity remains unclear. However, it is potentially telling that the region of cGAS used to bind the ribosome is also used to regulate its activity, both negatively and positively, by nucleosomes and free DNA (Zhang et al., 2020), respectively. The structure of cGAS bound to a ribosome, or collided ribosomes, would provide important insight into these molecular details; such work is in progress.

Ribosomal stalling during protein synthesis can result in the generation of potentially deleterious polypeptides, which are marked for degradation while still associated with the translating ribosome (Brandman and Hegde, 2016; Joazeiro, 2017, 2019). Importantly, however, it seems probable that ribosome stalling is not, in itself, sensed as pathological by cells, but that it is instead ribosome collision that has this effect (Ikeuchi et al., 2019; Juszkiewicz et al., 2018; Liu et al., 2013). The RQC pathway is crucial for dealing with collided ribosomes, but its wider physiological role has remained unclear. Our study now indicates that RQC initiator proteins suppress the innate immune response: the key proteins ASCC3, ZNF598, and RACK1 allow the disassembly of collided ribosomes, which are otherwise sensed as deleterious by cGAS (Juszkiewicz et al., 2018, 2020). Several results presented here are consistent with this idea. First, cGAS specifically and preferentially binds collided ribosomes *in vitro*. Second, cells depleted for ASCC3, ZNF598, and RACK1 display persistent activation of the cGAS-STING pathway, while depletion of proteins acting later in RQC, such as NEMF and Hbs1, do not. Third, cGAS sub-cellular localization changes dramatically when the RQC pathway is inhibited. Moreover, cGAS senses collided ribosomes induced by translation stress also in normal cells, including those elicited by chemically induced ribosome collision and heat shock. Crucially, it has previously been shown that ASCC3 or ZNF598 deficiency induces not only ISG expression but also a broad antiviral state (Li et al., 2013; Williamson et al., 2017; DiGiuseppe et al., 2018): in the absence of these RQC factors, cells activate the inflammatory response to an extent that is sufficient to inhibit virus infection (Li et al., 2013; DiGiuseppe et al., 2018). Interestingly, ISG genes activated by ASCC3 deficiency do not include the IFNs themselves (Li et al., 2013; and this work), but the cGAS-dependent gene expression program elicited is nevertheless sufficient to provide viral immunity (Li et al., 2013). This indicates that the innate immune response elicited by the gene expression program studied here is of significant biological consequence.

Interestingly, recent studies indicate that ZNF598 and RACK1 may also suppress the innate immune response to viral infection via the RIG-I-MAVS signaling pathway, responsible for sensing cytosolic RNAs (Wang et al., 2019; Xie et al., 2019). This could suggest that these RQC initiators also have other roles and/or are capable of suppressing the type I IFN expression via other mechanisms as well. We note that ASCC3 depletion leads to increased STING levels (Figures 1D, S1E, and S2A), which might in turn contribute to elevated ISG expression as well. Together, these data suggest that the RQC regulates ISG expression via multiple signaling pathways.

Somewhat surprisingly for a cytosolic DNA sensor, cGAS is broadly important for the anti-viral defense, including against numerous RNA viruses, which do not use a DNA intermediate (Schoggins et al., 2011, 2014; Li et al., 2013). It has been proposed that this might be because of increased release of mitochondrial DNA during virus infection (Ablasser and Chen, 2019). However, our data now raise the exciting possibility that cGAS directly senses the dramatic subversion of host-cell protein production occurring during virus infection. Indeed, it seems certain that high-level viral protein production will result in increased ribosome collision, potentially overloading the RQC

pathway and triggering cGAS-dependent signaling. Moreover, many viruses use folded RNA elements within their coding region to regulate translation and allow the decoding of alternative, or extended, coding frames. Indeed, translation mechanisms frequently employed by viruses, such as programmed ribosome frameshifting, stop-codon readthrough, and termination-dependent re-initiation (Walsh and Mohr, 2011; Firth and Brierley, 2012; Jaafar and Kieft, 2019) would by their very nature be expected to entail substantial levels of ribosome collision on the highly translated viral RNA. We propose that such translation stress could result in cGAS binding and co-activation (Figure 7).

It is important to stress that the potential role of cGAS in sensing translation stress outlined here in no way challenges prior work on cGAS as a crucial DNA sensor in innate immunity; rather, it expands the role of this intriguing protein so that it becomes an optimal cellular tool in the defense against microbial invasion. Indeed, it seems obvious to suggest that a bifurcate mechanism, entailing detection of both free DNA and translation stress by the same protein, would be ideally suited to provide the specificity and sensitivity required for an effective innate immune system.

Limitations of the study

This study shows that orthogonal perturbations of mRNA translation that result in collided ribosomes also induce re-localization of cGAS to the cytosol. This can in turn result in activation of the cGAS-STING-TBK1 pathway and of IRF3, which induces the expression of ISGs. Intriguingly, however, we were unable to detect increased expression of IFN itself, in line with work on ASCC3 deficiency from the Diamond laboratory (Li et al., 2013), which nevertheless showed that the response generated was sufficient to elicit protection against virus infection. The lack of IFN induction is interesting and surprising because activation by exogenous DNA and viruses generally results in potent IFN induction. However, these cellular stressors activate other receptors and pathways besides cGAS-STING (Thaiss et al., 2016), which together might be required for potent IFN induction. Interestingly, multiple studies have shown that a subset of ISG expression can be induced by IRF3 independently of type I IFNs (Guo et al., 2000; Grandvaux et al., 2002; Ashley et al., 2019).

A separation-of-function mutation, which disrupts the interaction between cGAS and ribosomes but retains its DNA-stimulated activity, would be an ideal tool to address the extent to which ribosomes generally regulate cGAS function. However, our interaction site mapping shows that it is cGAS DNA binding site B that is (also) important for binding ribosomes, which has so far made it impossible to separate the regulation by ribosomes from that by DNA or nucleosomes. Solving the structure of the cGAS-ribosome complex will hopefully provide the knowledge required to generate such a mutant.

STAR★METHODS

Detailed methods are provided in the online version of this paper and include the following:

- KEY RESOURCES TABLE
- RESOURCE AVAILABILITY
  - Lead contact
  - Materials availability
  - Data and code availability
- EXPERIMENTAL MODEL AND SUBJECT DETAILS
  - Cell lines and culture conditions
- METHOD DETAILS
  - Plasmid construction
  - Generation of stable cell lines
  - Co-immunoprecipitation
  - Western blotting
  - SILAC-based method for quantitative proteomic analysis
  - Sample preparation prior to mass spectrometry analysis
  - Mass spectrometry data acquisition
  - Mass spectrometry data analysis
  - Cytosol extraction for examination of cGAS-ribosome interaction
  - Ribosome purification from HEK293
  - Recombinant cGAS purification
  - Sucrose gradient fractionation
  - *In vitro* cGAS and ribosome binding assay
  - Cyclic dinucleotide synthesis assays
  - RNA interference
  - TT_{chem}-seq
  - Reverse transcription quantitative PCR (qRT-PCR)
  - Generation of collided ribosome and purification from rabbit reticulocyte lysate (RRL)
  - Digestion of polysomes with nuclease
  - Flow cytometry analysis
  - Immunofluorescence staining
  - Proximity ligation assay (*in situ* PLA)
  - cGAMP measurement *in vivo*
  - Micronucleus measurement
- QUANTIFICATION AND STATISTICAL ANALYSIS

SUPPLEMENTAL INFORMATION

Supplemental information can be found online at https://doi.org/10.1016/j.molcel.2021.05.018.

ACKNOWLEDGMENTS

This work was supported by the Francis Crick Institute (FCI receives funding from Cancer Research UK [FC001166], the UK Medical Research Council [FC001166], and the Wellcome Trust [FC001166]), grants to J.Q.S. from the European Research Council (Agreement 693327), a Laureate grant from the Novo Nordisk Foundation (NNF19OC0055875), and a Chair grant from the Danish National Research Foundation (DNRF153). Work in the Hegde lab was supported by the UK Medical Research Council (MC_UP_A022_1007 to R.S.H.). L.W. and Z.H. were supported by the EMBO-LTF program (EMBO ALTF 1071-2015 and EMBO ALTF 5-2019, respectively). We thank Anna Herlihy and Jane Walker for assistance with PLA experiments, and members of FCI's Advanced Sequencing Facility, Cell Services, Flow cytometry facility, and Advanced Light Microscopy for expert technical assistance. The Flp-In compatible destination vectors, Flp-In T-REx U2OS cell line, and STING cDNA were kindly provided by Markus Landthaler, Erich Nigg, and Pinglong Xu, respectively. We thank Qian Zhang, Annemarthe Van der Veen, Joan Manils, Subramanian Venkatesan, Marie-Luise Winz, Yisui Xia, Zhewang Lin, Shuyang Chen, and Viswanathan Chandrasekaran for their input and all Svejstrup lab members for discussions. We

thank Barbara Dirac-Svejstrup, Peter Verrijzer, and David Lopez Martinez for comments on the manuscript.

AUTHOR CONTRIBUTIONS

L.W. and J.Q.S. conceived the project. L.W. performed the majority of experiments, but S.J., P.K.B., Z.H., and P.F. helped L.W. generate collided ribosomes, purify recombinant cGAS, establish the cGAM *in vitro* assay, and perform mass spectrometry on interactomes, respectively. D.B. performed and analyzed flow cytometry experiments and also performed *in vivo* cGAMP measurements. R.M. analyzed TT_{chem}-seq data. A.S., A.P.S., R.S.H., and J.Q.S. supervised the work. L.W. and J.Q.S. wrote the manuscript, with input from all authors.

DECLARATION OF INTERESTS

The authors declare no competing interests.

Received: April 9, 2021
Revised: April 23, 2021
Accepted: May 13, 2021
Published: June 9, 2021

REFERENCES

Ablasser, A., and Chen, Z.J. (2019). cGAS in action: Expanding roles in immunity and inflammation. Science *363*, eaat8657.

Ablasser, A., Goldeck, M., Cavlar, T., Deimling, T., Witte, G., Röhl, I., Hopfner, K.P., Ludwig, J., and Hornung, V. (2013a). cGAS produces a 2′-5′-linked cyclic dinucleotide second messenger that activates STING. Nature *498*, 380–384.

Ablasser, A., Hertrich, C., Waßermann, R., and Hornung, V. (2013b). Nucleic acid driven sterile inflammation. Clin. Immunol. *147*, 207–215.

Andreeva, L., Hiller, B., Kostrewa, D., Lässig, C., de Oliveira Mann, C.C., Jan Drexler, D., Maiser, A., Gaidt, M., Leonhardt, H., Hornung, V., and Hopfner, K.P. (2017). cGAS senses long and HMGB/TFAM-bound U-turn DNA by forming protein-DNA ladders. Nature *549*, 394–398.

Arquint, C., and Nigg, E.A. (2014). STIL microcephaly mutations interfere with APC/C-mediated degradation and cause centriole amplification. Curr. Biol. *24*, 351–360.

Ashley, C.L., Abendroth, A., McSharry, B.P., and Slobedman, B. (2019). Interferon-Independent Upregulation of Interferon-Stimulated Genes during Human Cytomegalovirus Infection is Dependent on IRF3 Expression. Viruses *11*, 246.

Atkins, J.F., Loughran, G., Bhatt, P.R., Firth, A.E., and Baranov, P.V. (2016). Ribosomal frameshifting and transcriptional slippage: From genetic steganography and cryptography to adventitious use. Nucleic Acids Res. *44*, 7007–7078.

Barnett, K.C., Coronas-Serna, J.M., Zhou, W., Ernandes, M.J., Cao, A., Kranzusch, P.J., and Kagan, J.C. (2019). Phosphoinositide Interactions Position cGAS at the Plasma Membrane to Ensure Efficient Distinction between Self- and Viral DNA. Cell *176*, 1432–1446.e11.

Bartsch, K., Knittler, K., Borowski, C., Rudnik, S., Damme, M., Aden, K., Spehlmann, M.E., Frey, N., Saftig, P., Chalaris, A., and Rabe, B. (2017). Absence of RNase H2 triggers generation of immunogenic micronuclei removed by autophagy. Hum. Mol. Genet. *26*, 3960–3972.

Boyer, J.A., Spangler, C.J., Strauss, J.D., Cesmat, A.P., Liu, P., McGinty, R.K., and Zhang, Q. (2020). Structural basis of nucleosome-dependent cGAS inhibition. Science *370*, 450–454.

Brandman, O., and Hegde, R.S. (2016). Ribosome-associated protein quality control. Nat. Struct. Mol. Biol. *23*, 7–15.

Brandman, O., Stewart-Ornstein, J., Wong, D., Larson, A., Williams, C.C., Li, G.W., Zhou, S., King, D., Shen, P.S., Weibezahn, J., et al. (2012). A ribosome-bound quality control complex triggers degradation of nascent peptides and signals translation stress. Cell *151*, 1042–1054.

Brickner, J.R., Soll, J.M., Lombardi, P.M., Vågbø, C.B., Mudge, M.C., Oyeniran, C., Rabe, R., Jackson, J., Sullender, M.E., Blazosky, E., et al. (2017). A ubiquitin-dependent signalling axis specific for ALKBH-mediated DNA dealkylation repair. Nature *551*, 389–393.

Brubaker, S.W., Bonham, K.S., Zanoni, I., and Kagan, J.C. (2015). Innate immune pattern recognition: a cell biological perspective. Annu. Rev. Immunol. *33*, 257–290.

Chen, Q., Sun, L., and Chen, Z.J. (2016). Regulation and function of the cGAS-STING pathway of cytosolic DNA sensing. Nat. Immunol. *17*, 1142–1149.

Civril, F., Deimling, T., de Oliveira Mann, C.C., Ablasser, A., Moldt, M., Witte, G., Hornung, V., and Hopfner, K.P. (2013). Structural mechanism of cytosolic DNA sensing by cGAS. Nature *498*, 332–337.

Collart, M.A., and Weiss, B. (2020). Ribosome pausing, a dangerous necessity for co-translational events. Nucleic Acids Res. *48*, 1043–1055.

Crow, Y.J., and Manel, N. (2015). Aicardi-Goutières syndrome and the type I interferonopathies. Nat. Rev. Immunol. *15*, 429–440.

Dango, S., Mosammaparast, N., Sowa, M.E., Xiong, L.J., Wu, F., Park, K., Rubin, M., Gygi, S., Harper, J.W., and Shi, Y. (2011). DNA unwinding by ASCC3 helicase is coupled to ALKBH3-dependent DNA alkylation repair and cancer cell proliferation. Mol. Cell *44*, 373–384.

DiGiuseppe, S., Rollins, M.G., Bartom, E.T., and Walsh, D. (2018). ZNF598 Plays Distinct Roles in Interferon-Stimulated Gene Expression and Poxvirus Protein Synthesis. Cell Rep. *23*, 1249–1258.

Du, M., and Chen, Z.J. (2018). DNA-induced liquid phase condensation of cGAS activates innate immune signaling. Science *361*, 704–709.

Ergun, S.L., and Li, L. (2020). Structural Insights into STING Signaling. Trends Cell Biol. *30*, 399–407.

Firth, A.E., and Brierley, I. (2012). Non-canonical translation in RNA viruses. J. Gen. Virol. *93*, 1385–1409.

Gao, P., Ascano, M., Wu, Y., Barchet, W., Gaffney, B.L., Zillinger, T., Serganov, A.A., Liu, Y., Jones, R.A., Hartmann, G., et al. (2013). Cyclic [G(2′,5′)pA(3′,5′)p] is the metazoan second messenger produced by DNA-activated cyclic GMP-AMP synthase. Cell *153*, 1094–1107.

Gekara, N.O., and Jiang, H. (2019). The innate immune DNA sensor cGAS: A membrane, cytosolic, or nuclear protein? Sci. Signal. *12*, eaax3521.

Gentili, M., Lahaye, X., Nadalin, F., Nader, G.P.F., Puig Lombardi, E., Herve, S., De Silva, N.S., Rookhuizen, D.C., Zueva, E., Goudot, C., et al. (2019). The N-Terminal Domain of cGAS Determines Preferential Association with Centromeric DNA and Innate Immune Activation in the Nucleus. Cell Rep. *26*, 2377–2393.e13.

Grandvaux, N., Servant, M.J., tenOever, B., Sen, G.C., Balachandran, S., Barber, G.N., Lin, R., and Hiscott, J. (2002). Transcriptional profiling of interferon regulatory factor 3 target genes: direct involvement in the regulation of interferon-stimulated genes. J. Virol. *76*, 5532–5539.

Gregersen, L.H., Mitter, R., and Svejstrup, J.Q. (2020). Using TT_{chem}-seq for profiling nascent transcription and measuring transcript elongation. Nat. Protoc. *15*, 604–627.

Guo, J., Peters, K.L., and Sen, G.C. (2000). Induction of the human protein P56 by interferon, double-stranded RNA, or virus infection. Virology *267*, 209–219.

Harding, S.M., Benci, J.L., Irianto, J., Discher, D.E., Minn, A.J., and Greenberg, R.A. (2017). Mitotic progression following DNA damage enables pattern recognition within micronuclei. Nature *548*, 466–470.

Hashimoto, S., Sugiyama, T., Yamazaki, R., Nobuta, R., and Inada, T. (2020). Identification of a novel trigger complex that facilitates ribosome-associated quality control in mammalian cells. Sci. Rep. *10*, 3422.

Hopfner, K.P., and Hornung, V. (2020). Molecular mechanisms and cellular functions of cGAS-STING signalling. Nat. Rev. Mol. Cell Biol. *21*, 501–521.

Hornung, V., Ellegast, J., Kim, S., Brzózka, K., Jung, A., Kato, H., Poeck, H., Akira, S., Conzelmann, K.K., Schlee, M., et al. (2006). 5′-Triphosphate RNA is the ligand for RIG-I. Science *314*, 994–997.

Ikeuchi, K., Tesina, P., Matsuo, Y., Sugiyama, T., Cheng, J., Saeki, Y., Tanaka, K., Becker, T., Beckmann, R., and Inada, T. (2019). Collided ribosomes form a

CellPress
OPEN ACCESS

unique structural interface to induce Hel2-driven quality control pathways. EMBO J *38*, e100276.

Inada, T. (2020). Quality controls induced by aberrant translation. Nucleic Acids Res. *48*, 1084–1096.

Ishimura, R., Nagy, G., Dotu, I., Zhou, H., Yang, X.L., Schimmel, P., Senju, S., Nishimura, Y., Chuang, J.H., and Ackerman, S.L. (2014). RNA function. Ribosome stalling induced by mutation of a CNS-specific tRNA causes neurodegeneration. Science *345*, 455–459.

Jaafar, Z.A., and Kieft, J.S. (2019). Viral RNA structure-based strategies to manipulate translation. Nat. Rev. Microbiol. *17*, 110–123.

Joazeiro, C.A.P. (2017). Ribosomal Stalling During Translation: Providing Substrates for Ribosome-Associated Protein Quality Control. Annu. Rev. Cell Dev. Biol. *33*, 343–368.

Joazeiro, C.A.P. (2019). Mechanisms and functions of ribosome-associated protein quality control. Nat. Rev. Mol. Cell Biol. *20*, 368–383.

Jung, D.J., Sung, H.S., Goo, Y.W., Lee, H.M., Park, O.K., Jung, S.Y., Lim, J., Kim, H.J., Lee, S.K., Kim, T.S., et al. (2002). Novel transcription coactivator complex containing activating signal cointegrator 1. Mol. Cell. Biol. *22*, 5203–5211.

Juszkiewicz, S., and Hegde, R.S. (2017). Initiation of Quality Control during Poly(A) Translation Requires Site-Specific Ribosome Ubiquitination. Mol. Cell *65*, 743–750.e4.

Juszkiewicz, S., Chandrasekaran, V., Lin, Z., Kraatz, S., Ramakrishnan, V., and Hegde, R.S. (2018). ZNF598 Is a Quality Control Sensor of Collided Ribosomes. Mol. Cell *72*, 469–481.e7.

Juszkiewicz, S., Speldewinde, S.H., Wan, L., Svejstrup, J.Q., and Hegde, R.S. (2020). The ASC-1 Complex Disassembles Collided Ribosomes. Mol. Cell *79*, 603–614.e8.

Kato, K., Omura, H., Ishitani, R., and Nureki, O. (2017). Cyclic GMP-AMP as an Endogenous Second Messenger in Innate Immune Signaling by Cytosolic DNA. Annu. Rev. Biochem. *86*, 541–566.

Kranzusch, P.J., Lee, A.S., Berger, J.M., and Doudna, J.A. (2013). Structure of human cGAS reveals a conserved family of second-messenger enzymes in innate immunity. Cell Rep. *3*, 1362–1368.

Kujirai, T., Zierhut, C., Takizawa, Y., Kim, R., Negishi, L., Uruma, N., Hirai, S., Funabiki, H., and Kurumizaka, H. (2020). Structural basis for the inhibition of cGAS by nucleosomes. Science *370*, 455–458.

Lawrence, M., Huber, W., Pagès, H., Aboyoun, P., Carlson, M., Gentleman, R., Morgan, M.T., and Carey, V.J. (2013). Software for computing and annotating genomic ranges. PLoS Comput. Biol. *9*, e1003118.

Letzring, D.P., Wolf, A.S., Brule, C.E., and Grayhack, E.J. (2013). Translation of CGA codon repeats in yeast involves quality control components and ribosomal protein L1. RNA *19*, 1208–1217.

Li, T., and Chen, Z.J. (2018). The cGAS-cGAMP-STING pathway connects DNA damage to inflammation, senescence, and cancer. J. Exp. Med. *215*, 1287–1299.

Li, J., Ding, S.C., Cho, H., Chung, B.C., Gale, M., Jr., Chanda, S.K., and Diamond, M.S. (2013). A short hairpin RNA screen of interferon-stimulated genes identifies a novel negative regulator of the cellular antiviral response. MBio *4*, e00385-13.

Liu, B., Han, Y., and Qian, S.B. (2013). Cotranslational response to proteotoxic stress by elongation pausing of ribosomes. Mol. Cell *49*, 453–463.

Love, M.I., Huber, W., and Anders, S. (2014). Moderated estimation of fold change and dispersion for RNA-seq data with DESeq2. Genome Biol. *15*, 550.

Lum, K.K., Song, B., Federspiel, J.D., Diner, B.A., Howard, T., and Cristea, I.M. (2018). Interactome and Proteome Dynamics Uncover Immune Modulatory Associations of the Pathogen Sensing Factor cGAS. Cell Syst. *7*, 627–642.e6.

Mackenzie, K.J., Carroll, P., Martin, C.A., Murina, O., Fluteau, A., Simpson, D.J., Olova, N., Sutcliffe, H., Rainger, J.K., Leitch, A., et al. (2017). cGAS surveillance of micronuclei links genome instability to innate immunity. Nature *548*, 461–465.

Matsuo, Y., Ikeuchi, K., Saeki, Y., Iwasaki, S., Schmidt, C., Udagawa, T., Sato, F., Tsuchiya, H., Becker, T., Tanaka, K., et al. (2017). Ubiquitination of stalled ribosome triggers ribosome-associated quality control. Nat. Commun. *8*, 159.

Matsuo, Y., Tesina, P., Nakajima, S., Mizuno, M., Endo, A., Buschauer, R., Cheng, J., Shounai, O., Ikeuchi, K., Saeki, Y., et al. (2020). RQT complex dissociates ribosomes collided on endogenous RQC substrate SDD1. Nat. Struct. Mol. Biol. *27*, 323–332.

McNab, F., Mayer-Barber, K., Sher, A., Wack, A., and O'Garra, A. (2015). Type I interferons in infectious disease. Nat. Rev. Immunol. *15*, 87–103.

Merret, R., Nagarajan, V.K., Carpentier, M.C., Park, S., Favory, J.J., Descombin, J., Picart, C., Charng, Y.Y., Green, P.J., Deragon, J.M., and Bousquet-Antonelli, C. (2015). Heat-induced ribosome pausing triggers mRNA co-translational decay in Arabidopsis thaliana. Nucleic Acids Res. *43*, 4121–4132.

Michalski, S., de Oliveira Mann, C.C., Stafford, C.A., Witte, G., Bartho, J., Lammens, K., Hornung, V., and Hopfner, K.P. (2020). Structural basis for sequestration and autoinhibition of cGAS by chromatin. Nature *587*, 678–682.

Nass, M.M. (1972). Differential effects of ethidium bromide on mitochondrial and nuclear DNA synthesis in vivo in cultured mammalian cells. Exp. Cell Res. *72*, 211–222.

Orzalli, M.H., Broekema, N.M., Diner, B.A., Hancks, D.C., Elde, N.C., Cristea, I.M., and Knipe, D.M. (2015). cGAS-mediated stabilization of IFI16 promotes innate signaling during herpes simplex virus infection. Proc. Natl. Acad. Sci. USA *112*, E1773–E1781.

Pathare, G.R., Decout, A., Glück, S., Cavadini, S., Makasheva, K., Hovius, R., Kempf, G., Weiss, J., Kozicka, Z., Guey, B., et al. (2020). Structural mechanism of cGAS inhibition by the nucleosome. Nature *587*, 668–672.

Pichlmair, A., Schulz, O., Tan, C.P., Näslund, T.I., Liljeström, P., Weber, F., and Reis e Sousa, C. (2006). RIG-I-mediated antiviral responses to single-stranded RNA bearing 5′-phosphates. Science *314*, 997–1001.

Ran, F.A., Hsu, P.D., Wright, J., Agarwala, V., Scott, D.A., and Zhang, F. (2013). Genome engineering using the CRISPR-Cas9 system. Nat. Protoc. *8*, 2281–2308.

Schindelin, J., Arganda-Carreras, I., Frise, E., Kaynig, V., Longair, M., Pietzsch, T., Preibisch, S., Rueden, C., Saalfeld, S., Schmid, B., et al. (2012). Fiji: an open-source platform for biological-image analysis. Nat. Methods *9*, 676–682.

Schoggins, J.W., Wilson, S.J., Panis, M., Murphy, M.Y., Jones, C.T., Bieniasz, P., and Rice, C.M. (2011). A diverse range of gene products are effectors of the type I interferon antiviral response. Nature *472*, 481–485.

Schoggins, J.W., MacDuff, D.A., Imanaka, N., Gainey, M.D., Shrestha, B., Eitson, J.L., Mar, K.B., Richardson, R.B., Ratushny, A.V., Litvak, V., et al. (2014). Pan-viral specificity of IFN-induced genes reveals new roles for cGAS in innate immunity. Nature *505*, 691–695.

Shalgi, R., Hurt, J.A., Krykbaeva, I., Taipale, M., Lindquist, S., and Burge, C.B. (2013). Widespread regulation of translation by elongation pausing in heat shock. Mol. Cell *49*, 439–452.

Sharma, A., Mariappan, M., Appathurai, S., and Hegde, R.S. (2010). In vitro dissection of protein translocation into the mammalian endoplasmic reticulum. Methods Mol. Biol. *619*, 339–363.

Shoemaker, C.J., and Green, R. (2012). Translation drives mRNA quality control. Nat. Struct. Mol. Biol. *19*, 594–601.

Simms, C.L., Yan, L.L., and Zaher, H.S. (2017). Ribosome Collision Is Critical for Quality Control during No-Go Decay. Mol. Cell *68*, 361–373.e5.

Subramanian, A., Tamayo, P., Mootha, V.K., Mukherjee, S., Ebert, B.L., Gillette, M.A., Paulovich, A., Pomeroy, S.L., Golub, T.R., Lander, E.S., and Mesirov, J.P. (2005). Gene set enrichment analysis: a knowledge-based approach for interpreting genome-wide expression profiles. Proc. Natl. Acad. Sci. USA *102*, 15545–15550.

Sun, L., Wu, J., Du, F., Chen, X., and Chen, Z.J. (2013). Cyclic GMP-AMP synthase is a cytosolic DNA sensor that activates the type I interferon pathway. Science *339*, 786–791.

Sundaramoorthy, E., Leonard, M., Mak, R., Liao, J., Fulzele, A., and Bennett, E.J. (2017). ZNF598 and RACK1 Regulate Mammalian Ribosome-

Associated Quality Control Function by Mediating Regulatory 40S Ribosomal Ubiquitylation. Mol. Cell *65*, 751–760.e4.

Takeuchi, O., and Akira, S. (2010). Pattern recognition receptors and inflammation. Cell *140*, 805–820.

Tan, X., Sun, L., Chen, J., and Chen, Z.J. (2018). Detection of Microbial Infections Through Innate Immune Sensing of Nucleic Acids. Annu. Rev. Microbiol. *72*, 447–478.

Tao, J., Zhang, X.W., Jin, J., Du, X.X., Lian, T., Yang, J., Zhou, X., Jiang, Z., and Su, X.D. (2017). Nonspecific DNA Binding of cGAS N Terminus Promotes cGAS Activation. J. Immunol. *198*, 3627–3636.

Thaiss, C.A., Levy, M., Itav, S., and Elinav, E. (2016). Integration of Innate Immune Signaling. Trends Immunol. *37*, 84–101.

Tyanova, S., Temu, T., Sinitcyn, P., Carlson, A., Hein, M.Y., Geiger, T., Mann, M., and Cox, J. (2016). The Perseus computational platform for comprehensive analysis of (prote)omics data. Nat. Methods *13*, 731–740.

Volkman, H.E., Cambier, S., Gray, E.E., and Stetson, D.B. (2019). Tight nuclear tethering of cGAS is essential for preventing autoreactivity. eLife *8*, e47491.

Walsh, D., and Mohr, I. (2011). Viral subversion of the host protein synthesis machinery. Nat. Rev. Microbiol. *9*, 860–875.

Wan, L., Lou, J., Xia, Y., Su, B., Liu, T., Cui, J., Sun, Y., Lou, H., and Huang, J. (2013). hPrimpol1/CCDC111 is a human DNA primase-polymerase required for the maintenance of genome integrity. EMBO Rep. *14*, 1104–1112.

Wang, G., Kouwaki, T., Okamoto, M., and Oshiumi, H. (2019). Attenuation of the Innate Immune Response against Viral Infection Due to ZNF598-Promoted Binding of FAT10 to RIG-I. Cell Rep. *28*, 1961–1970.e4.

West, A.P., Khoury-Hanold, W., Staron, M., Tal, M.C., Pineda, C.M., Lang, S.M., Bestwick, M., Duguay, B.A., Raimundo, N., MacDuff, D.A., et al. (2015). Mitochondrial DNA stress primes the antiviral innate immune response. Nature *520*, 553–557.

Williamson, L., Saponaro, M., Boeing, S., East, P., Mitter, R., Kantidakis, T., Kelly, G.P., Lobley, A., Walker, J., Spencer-Dene, B., et al. (2017). UV Irradiation Induces a Non-coding RNA that Functionally Opposes the Protein Encoded by the Same Gene. Cell *168*, 843–855.e13.

Xie, W., and Patel, D.J. (2020). Keeping innate immune response in check: when cGAS meets the nucleosome. Cell Res. *30*, 1055–1056.

Xie, T., Chen, T., Li, C., Wang, W., Cao, L., Rao, H., Yang, Q., Shu, H.B., and Xu, L.G. (2019). RACK1 attenuates RLR antiviral signaling by targeting VISA-TRAF complexes. Biochem. Biophys. Res. Commun. *508*, 667–674.

Yang, H., Wang, H., Ren, J., Chen, Q., and Chen, Z.J. (2017). cGAS is essential for cellular senescence. Proc. Natl. Acad. Sci. USA *114*, E4612–E4620.

Zhang, X., Shi, H., Wu, J., Zhang, X., Sun, L., Chen, C., and Chen, Z.J. (2013). Cyclic GMP-AMP containing mixed phosphodiester linkages is an endogenous high-affinity ligand for STING. Mol. Cell *51*, 226–235.

Zhang, X., Wu, J., Du, F., Xu, H., Sun, L., Chen, Z., Brautigam, C.A., Zhang, X., and Chen, Z.J. (2014). The cytosolic DNA sensor cGAS forms an oligomeric complex with DNA and undergoes switch-like conformational changes in the activation loop. Cell Rep. *6*, 421–430.

Zhang, X., Bai, X.C., and Chen, Z.J. (2020). Structures and Mechanisms in the cGAS-STING Innate Immunity Pathway. Immunity *53*, 43–53.

Zhao, B., Xu, P., Rowlett, C.M., Jing, T., Shinde, O., Lei, Y., West, A.P., Liu, W.R., and Li, P. (2020). The molecular basis of tight nuclear tethering and inactivation of cGAS. Nature *587*, 673–677.

Zhou, W., Whiteley, A.T., de Oliveira Mann, C.C., Morehouse, B.R., Nowak, R.P., Fischer, E.S., Gray, N.S., Mekalanos, J.J., and Kranzusch, P.J. (2018). Structure of the Human cGAS-DNA Complex Reveals Enhanced Control of Immune Surveillance. Cell *174*, 300–311.e11.

Zierhut, C., Yamaguchi, N., Paredes, M., Luo, J.D., Carroll, T., and Funabiki, H. (2019). The Cytoplasmic DNA Sensor cGAS Promotes Mitotic Cell Death. Cell *178*, 302–315.e23.

CellPress
OPEN ACCESS

STAR★METHODS

KEY RESOURCES TABLE

REAGENT or RESOURCE	SOURCE	IDENTIFIER
Antibodies		
Vinculin	Sigma	Cat# V9131; RRID: AB_477629
Histone H3	Abcam	Cat# ab18521; RRID: AB_732917
hcGAS	Cell signaling Technology	Cat# 15102; RRID: AB_2732795
STING	Cell signaling Technology	Cat# 13647; RRID: AB_2732796
IRF3	Cell signaling Technology	Cat# 4302; RRID: AB_1904036
P-IRF3	Cell signaling Technology	Cat# 4947; RRID: AB_823547
TBK1	Cell signaling Technology	Cat# 3504; RRID: AB_2255663
P-TBK1	Cell signaling Technology	Cat# 5483; RRID: AB_10693472
ASCC3	Dango et al., 2011	N/A
ASCC2	Abcam	Cat# Ab168811; RRID: AB_2832200
ASCC1	Proteintech	Cat# 12301-1-AP; RRID: AB_2059350
TRIP4	NOVUS	Cat# NB100-419; RRID: AB_10000684
uL2	Abcam	Cat# Ab169538; RRID: AB_2714187
eS24	Abcam	Cat# Ab196652; RRID: AB_2714188
uS3	Bethyl	Cat# A303-840A; RRID: AB_2620191
uS5	Bethyl	Cat# A303-794A; RRID: AB_11218192
eS10	Abcam	Cat# Ab151550; RRID: AB_2714147
eS8	Abcam	Cat# Ab201454 RRID: AB_2833046
GFP	Cell signaling Technology	Cat# 2956; RRID:AB_1196615
MAVS	Cell signaling Technology	Cat# 3993; RRID: AB_1196615
P-STAT1	Cell signaling Technology	Cat# 9167; RRID: AB_561284
STAT1	Cell signaling Technology	Cat# #9172; RRID:AB_2198300
RSAD2	Cell signaling Technology	Cat#13996; RRID:AB_2734772
IFIT2	Proteintech	Cat# 12604-1-AP; RRID: AB_2864734
Flag	Sigma	Cat# F1804; RRID: AB_262044
RACK1	Abcam	Cat# ab62735; RRID: AB_956255
ZNF598	GeneTex	Cat# GTX119245; RRID: AB_10619017
γH2AX	Sigma	Cat# 05-636; RRID: AB_309864
dsDNA	Abcam	Cat# ab27156; RRID: AB_470907
Goat anti-Rabbit Alexa Fluor 594	ThermoFisher Scientific	Cat# A-11012; RRID: AB_2534079
Goat anti-mouse Alexa Fluor 594	ThermoFisher Scientific	Cat# A-11005; RRID: AB_2534073
Bacterial and virus strains		
BL21 DE3	ThermoFisher Scientific	C600003
NEB 5-alpha Competent *E. coli*	New England Biolabs	C2987H
One Shot ccdB Survival 2 T1R Competent Cells	ThermoFisher Scientific	A10460
Chemicals, peptides, and recombinant proteins		
Doxycycline	Clontech	8634-1
3xFLAG peptide	Peptide Chemistry, The Francis Crick Institute	N/A
4-thiouridine	Glentham Life Sciences	GN6085
4-thiouracil	Sigma-Aldrich	440736

(*Continued on next page*)

Continued		
REAGENT or RESOURCE	SOURCE	IDENTIFIER
MTSEA biotin-XX linker ((MTSEA Biotincapcap; 2-((6-((6-((biotinoyl)amino) hexanoyl)amino)hexanoyl) amino) ethylmethanethiosulfonate))	Biotium	BT90066
Cycloheximide	Sigma-Aldrich	C4859-1ML
Emetine dihydrochloride	BioVision	1970-50
Anisomycin	APExBIO	B6674
PreScission Protease	GenScript	Z02799
cGAS recombinant protein	This paper	N/A
cGAS-8his recombinant protein	This paper	N/A
hPrimpol1-8his recombinant protein	This paper	N/A
eRF1-AAQ (human)	Juszkiewicz et al., 2018	N/A
Critical commercial assays		
Q5® Site-Directed Mutagenesis Kit	New England Biolabs	E0554S
RNeasy kit	QIAGEN	74104
miRNeasy kit	QIAGEN	217004
RNA minElute clean-up kit	QIAGEN	74204
PureLink RNA Mini kit	Thermo Fisher Scientific	12183020
2′,3′-Cyclic GAMP Direct EIA Kit	2Bscientific	K067-H1
mMACS Streptavidin Kit	Miltenyi	130-074-101
Taqman Reverse Transcriptase Reagents	Thermo Fisher Scientific	N8080234
SilverQuest Silver Staining Kit	Thermo Fisher Scientific	LC6070
Duolink *In Situ* Red Starter Kit Mouse/ Rabbit	Sigma	DUO92101
Experimental models: Cell lines		
HEK293	The Francis Crick Institute Cell Services	N/A
HEK293T	The Francis Crick Institute Cell Services	N/A
U2OS	The Francis Crick Institute Cell Services	N/A
MRC5VA	The Francis Crick Institute Cell Services	N/A
Flp-In T-REx HEK293	ThermoFisher Scientific	R78007
Flp-In T-REx U2OS	Arquint and Nigg, 2014	N/A
MRC5VA Parent	This paper	N/A
MRC5VA KO-7	This paper	N/A
MRC5VA KO-12	This paper	N/A
Flp-In T-REx U2OS Parent	This paper	N/A
Flp-In T-REx U2OS KO-16	This paper	N/A
Flp-In T-REx U2OS KO-23	This paper	N/A
Flp-In T-REx HEK293-K^{AAA}0	This paper	N/A
Flp-In T-REx HEK293-K^{AAA}20	This paper	N/A
Flp-In T-REx U2OS *CGAS*KO16-GFP-Vector	This paper	N/A
Flp-In T-REx U2OS *CGAS*KO16-GFP-cGAS-WT	This paper	N/A
Flp-In T-REx U2OS *CGAS*KO16-GFP-cGAS-C396A	This paper	N/A
Flp-In T-REx U2OS *CGAS*KO16-GFP-cGAS-ED > AA	This paper	N/A
Flp-In T-REx U2OS *CGAS*KO16-GFP-cGAS-K411A	This paper	N/A

(*Continued on next page*)

Continued		
REAGENT or RESOURCE	SOURCE	IDENTIFIER
Flp-In T-REx U2OS *CGAS*KO16-GFP-cGAS- ΔZR	This paper	N/A
Flp-In T-REx U2OS *CGAS*KO16-GFP-cGAS-RBM(K/R-A)	This paper	N/A
Flp-In T-REx U2OS *CGAS*KO16-GFP-cGAS-RBM(K/R-E)	This paper	N/A
Flp-In T-REx U2OS *CGAS*KO16-Flag-HA-cGAS-WT	This paper	N/A
Flp-In T-REx U2OS *CGAS*KO16-cGAS (untagged)	This paper	N/A
HEK293T-HA-Flag-STING	This paper	N/A
Flp-In T-REx HEK293 Flag-HA-ASCC1	This paper	N/A
Flp-In T-REx HEK293 Flag-HA-ASCC2	This paper	N/A
Flp-In T-REx HEK293 Flag-HA-ASCC3	This paper	N/A
Oligonucleotides		
All oligonucleotides used in this study are listed in Table S3	This paper	N/A
Recombinant DNA		
pDONR223	Kind gift from Simon Boulton	N/A
pFRT/TO/GFP DEST	Kind gift from Markus Landthaler	N/A
pFRT/TO/FLAGHA DEST	Kind gift from Markus Landthaler	N/A
pFRT/TO	Kind gift from Markus Landthaler	N/A
pOG44	Thermo Fisher Scientific	V600520
pSpCas9n(BB)-2A-Puro (PX462) V2.0	Ran et al., 2013	Addgene Plasmid #62987
pGEX 6p-1	Sigma-Aldrich	GE28-9546-48
cDNA cGAS	Horizon discovery	MHS6278-202759247
cDNA ASCC1	Horizon discovery	MHS6278-202756253
cDNA ASCC2	Horizon discovery	MHS6278-202830549
cDNA ASCC3	Williamson et al., 2017	N/A
STING	Kind gift from PingLong Xu	N/A
Software and algorithms		
Fiji	Schindelin et al., 2012	https://imagej.net/Fiji
GraphPad prism 7	GraphPad	https://www.graphpad.com/scientific-software/prism/
FlowJo	FlowJo	https://www.flowjo.com/
Illustrator CC	Adobe	https://www.adobe.com/
Photoshop 2020	Adobe	https://www.adobe.com/
Perseus version 1.4.0.11	Tyanova et al., 2016	https://maxquant.net/perseus/
Other		
VECTASHIELD Antifade Mounting Medium	Vector Laboratories	H-1700
Protease Inhibitor Cocktail	Sigma-Aldrich	5056489001
PhosSTOP	Sigma-Aldrich	4906837001
Tet-free FBS	Clontech	631106
High glucose DMEM	Thermo Fisher Scientific	11965118
4-15% TGX gels (18wells)	Bio-Rad	56711084
4-15% TGX gels (26wells)	Bio-Rad	56711085
Nitrocellulose membrane	GE Healthcare Life Sciences	10600002
SuperSignal West Pico PLUS ECl reagent	Thermo Fisher Scientific	34577
SuperSignal West Dura ECl reagent	Thermo Fisher Scientific	34075

(*Continued on next page*)

Continued		
REAGENT or RESOURCE	SOURCE	IDENTIFIER
Instant Blue	Expedeon	ISB1L
iTaqUniversal SYBR Green Supermix	BioRad	172-5124
Benzonase	MerckMillipore	70746-4
Gateway LR Clonase II Enzyme	Thermo Fisher Scientific	11791020
Gateway BP Clonase II Enzyme	Thermo Fisher Scientific	11789100
Alkaline phosphatase	New England Biolabs	M0290
Lipofectamine 2000 Transfection Reagent	Thermo Fisher Scientific	11668019
Lipofectamine RNAiMAX Transfection Reagent	Thermo Fisher Scientific	13778150
HisPurNi-NTA magnetic beads	Thermo Fisher Scientific	88832
Glutathione agarose	Thermo Fisher Scientific	16101
ANTI-FLAG M2 Affinity Gel	Sigma-Aldrich	A2220
Protein G Agarose	Thermo Fisher Scientific	20398
Heparin HiTrap column	GE Life Sciences	GE17-0407-01
GFP-Trap magnetic Agarose beads	chromotek	gtma-20
3.5 ml, Open-Top Thickwall Polycarbonate Tube	Beckman Coulter	349622
230 μl, Tube, Thickwall, Polycarbonate, 7 × 20 mm	Beckman Coulter	343775
Ponceau S	Sigma-Aldrich	P7170
Herring Testis DNA	Sigma-Aldrich	D6898-250MG
Bio-Rad protein assay reagent	Bio-Rad	#5000006
Micrococcal Nuclease	New England Biolabs	M0247S
RNase inhibitor	Thermo Fisher Scientific	N8080119
TLC PEI-Cellulose F plate	Merck Millipore	105579
TRIzol Reagent	Thermo Fisher Scientific	15596026
Deposited data		
Images	This study	https://dx.doi.org/10.17632/35336dkyhw.1
Sequencing data	This study	GEO: GSE151127
Mass spectrometry data	This study	ProteomeXchange:PXD019359

RESOURCE AVAILABILITY

Lead contact

Further information and requests for resources and reagents should be directed to and will be fulfilled by the Lead Contact, Jesper Svejstrup (jsvejstrup@sund.ku.dk).

Materials availability

Plasmids will be deposited with and distributed by the non-profit distributor Addgene.

Data and code availability

- The mass spectrometry data are available via ProteomeXchange with identifier PXD019359.
- The TT-Seq data used in this study are available at GEO under accession number GSE151127.
- The original images of the study are at Mendeley https://dx.doi.org/10.17632/35336dkyhw.1

EXPERIMENTAL MODEL AND SUBJECT DETAILS

Cell lines and culture conditions

MRC5VA, HEK293, HEK293T, and U2OS (Human Osteosarcoma) were cultured in high glucose DMEM supplemented with 10% v/v FBS, 100 U/ml penicillin, 100 mg/ml streptomycin at 37°C with 5% CO_2. 15 μg/ml blasticidin and 100 μg/ml hygromycin were used for culturing Flp-In T-REx HEK293 or Flp-In T-REx U2OS stably expressing genes of interest. All cell lines were confirmed to be mycoplasma-free by the Francis Crick Institute Cell Services.

METHOD DETAILS

Plasmid construction

cDNAs of ASCC1, ASCC2, ASCC3, and cGAS were bought from Horizon, were amplified with primers adding attB1 and attB2 sequences (Table S3) and were cloned into the pDONR223 vector using the gateway BP recombinase system (Thermo Fisher Scientific, 11789020). All cGAS mutants were generated using the Q5® Site-Directed Mutagenesis Kit (New England Biolabs, E0554S) with specific primers (Table S3) and verified by sequencing. pDONR223 constructs were recombined into the pFRT/ TO/FLAG/HA-DEST or pFRT/TO/GFP-DEST destination vector using Gateway LR Clonase II Enzyme mix according to the manufacturer's protocol (Thermo Fisher Scientific, 11791020). pGEX6p-1 cGAS WT was generated using In-Fusion® HD Cloning Kit (Takara Bio USA, 102518). pGEX6p-1 cGAS 8his (c-terminus) was generated using the Q5® Site-Directed Mutagenesis Kit (New England Biolabs, E0554S). pGEX6p-1 hPrimpol1 was generated using BamHI-HF (New England Biolabs, R3136S) and NotI-HF (New England Biolabs, R3189S) restriction enzymes

Generation of stable cell lines

To generate cell lines expressing Flag-HA-tagged ASCC1, ASCC2, or ASCC3, Flp-In T-REx HEK293 cell lines were co-transfected with a 9:1 ratio of pOG44 Flp-recombinase expression vector (Thermo Fisher Scientific, V600520) and pFRT/TO/Flag/HA- ASCC1, ASCC2, or ASCC3 constructs using Lipofectamine 2000 (Thermo Fisher Scientific, 11668019) according to the manufacturer's instructions. Cells were seeded as single cells, 24 hours after transfection. The cell culture media was supplemented with 100 μg/ml hygromycin and 15 μg/ml blasticidin on the following day. The cells were allowed to grow for ten days. Single colonies were recovered and verified by western blotting using the following antibodies: Flag, ASCC1, ASCC2, or ASCC3. To generate cGAS knockout cells, MRC5VA or U2OS cells were transfected with the two pSpCas9n (BB)-2A-Puro (PX462) plasmids containing nickase gRNA pairs A and B, using Lipofectamine 2000 (Ran et al., 2013). The transfected cells were selected by supplementing cell culture media with 2 μg/ml puromycin for two days, and then seeded as single cells. cGAS knockout clones were verified by both western blotting and sequencing the indels in the cGAS genomic locus. Flp-In T-Rex U2OS cGAS knockout cell lines expressing GFP-tagged vector, cGAS, or cGAS mutants were constructed in a similar way to Fip-In T-Rex HEK293 cell lines expressing Flag-HA-tagged ASCC1.

Co-immunoprecipitation

In Figure 2D, one 150 mm dish of U2OS cGAS KO cell lines expressing GFP-vector or GFP-tagged cGAS was used for each sample. Cells were processed as described for cGAS interactome quantitative proteomic analysis below. The amount of lysis buffer and GFP-Trap magnetic Agarose beads were scaled down appropriately. The samples were subjected to western blot analysis after being eluted with SDS sample buffer at 95°C for 10 minutes.

Western blotting

For whole cell extracts, cell pellets were resuspended with lysis buffer (20 mM Tris-HCl pH 8.0, 100 mM NaCl, 0.5% (v/v) NP-40, 1 mM dithiothreitol (DTT), 250U/ml Benzonase, PhosSTOP (Sigma-Aldrich, 04906837001) and Protease Inhibitor Cocktail (Sigma-Aldrich, 05056489001) and left on ice for 20 minutes. Protein concentration was measured using Bio-Rad protein assay reagent (Bio-Rad, #5000006) and normalized to the sample with the lowest concentration. The samples were homogenized in 4x SDS sample buffer containing DTT (50 mM final concentration). After heating at 95°C for 10 minutes, the samples were separated on a 4%–15% TGX gels (Bio-Rad, 56711084/5) and transferred to nitrocellulose membrane (GE Healthcare Life Sciences, 10600002). Membranes were stained with Ponceau S (Sigma-Aldrich, P7170) to test for equal loading, followed by 30 minutes blocking in blocking buffer containing 5% (w/v) skimmed milk in TBS-T (TBS, 0.1% (v/v) Tween20). Membranes were incubated with primary antibody (in 5% (w/v) BSA in TBS-T containing 0.02% (w/v) sodium azide) overnight at 4°C, or at room temperature for 2 hours. Primary antibodies are listed in Key resources table. Membranes were washed three times in TBST and incubated with HRP-conjugated secondary antibodies in TBS-T with 5% (w/v) BSA (Sigma) and visualized using SuperSignal West Pico PLUS or Dura Chemiluminescent Substrate ECL reagent (Thermo Fisher Scientific, 34577 or 34075).

SILAC-based method for quantitative proteomic analysis

For mapping the cGAS interactome, Flp-In T-Rex U2OS cGAS KO cell lines expressing GFP-vector or GFP-tagged cGAS were cultured in SILAC light media or heavy media for 2 weeks. 98% efficiency of isotope incorporation was confirmed by mass spectrometry. Six 150 mm dishes of cells grown in light or heavy media were used for each sample. Cell pellets were resuspended in 5 mL buffer A (20 mM Tris-HCl pH 8.0, 150 mM NaCl, 0.05% (v/v) NP-40, 250U/ml Benzonase (Merck Millipore, 70746-4), 1.5 mM $MgCl_2$, 10% glycerol, PhosSTOP (Sigma-Aldrich, 4906837001) and Protease Inhibitor Cocktail (Sigma-Aldrich, 5056489001)) and incubated in cold room for 0.5 hour, and then centrifuged at 20000 g for 8 minutes at 4°C. Soluble fractions were kept, and insoluble fractions were resuspended in 600 μl buffer B (20 mM Tris-HCl pH 8.0, 500 mM NaCl, 0.05% (v/v) NP-40, 1 mM DTT, 250 U/ml Benzonase, 10% glycerol, PhosSTOP and Protease Inhibitor Cocktail) and incubated on ice for 20 minutes. Before centrifugation at 20000 g for 10 minutes at 4°C, the salt concentration was diluted to 150 mM with 1.4 mL buffer C (20 mM Tris-HCl pH 8.0, 0.05% (v/v) NP-40, 1 mM DTT, 250 U/ml Benzonase, 10% glycerol, PhosSTOP and Protease Inhibitor Cocktail). Supernatant was collected as chromatin fraction after centrifugation and pooled with soluble fraction as whole cell extract, which was in total 7 mL for each sample.

Whole cell extract was incubated with 125 μl GFP-Trap magnetic Agarose beads (chromotek, gtma-20) for 3 hours at 4°C. Beads were washed with 3 mL buffer A twice and eluted with 30 μl SDS sample buffer twice at 95°C for 10 minutes. In Figure 2B and 5 ul of elution samples was subjected to SDS-PAGE and stained using the SilverQuest Silver Staining Kit (Thermo Fisher Scientific, LC6070). For mass spectrometry, light isotope-labeled samples and heavy isotope-labeled samples were mixed before loading on SDS-PAGE gel, for example, light labeled GFP-vector samples were mixed with heavy-labeled GFP-tagged cGAS samples; heavy-labeled GFP-vector samples was mixed with light -labeled GFP-tagged cGAS samples. The mixed samples were run around 10 mm into the fixed 10% NuPAGE Bis-Tris and stained with Instant Blue (Expedeon, ISB1L).

Sample preparation prior to mass spectrometry analysis

Gel bands were excised, de-stained and then reduced (10 mM dithiothreitol) and alkylated (55 mM iodoacetamide) prior to overnight trypsin digest (100 ng, Pierce Trypsin Protease, MS Grade). The following day, peptides were extracted using a solution of 50% acetonitrile, 1% formic acid. Peptide samples were dried by vacuum centrifugation then re-solubilised in 0.1% trifluoroacetic acid prior to MS analysis.

Mass spectrometry data acquisition

A Thermo Fisher Scientific UltiMate 3000 UHPLC instrument loaded peptide samples onto a trap column (Acclaim PepMap 100 C18, 75 μm ID, 2 cm length, 3 μm particle size) for desalting. Peptides were transferred to an EASY-Spray analytical column (PepMap C18, 50 μm ID, 15 cm length, 2 μm particle size, 100 Å pore size) and separated using a 100-minute gradient of increasing organic solvent (80% acetonitrile, 5% dimethyl sulfoxide) from 8 to 32%. An orbitrap Fusion Lumos Tribrid (Thermo Fisher Scientific) mass spectrometer was operated in positive ionisation mode to acquire data. Instrument settings were: MS1 data were acquired in the orbitrap at a resolution of 120k, 4E6 AGC target, 50 ms maximum injection time, dynamic exclusion of ± 10 ppm and 60 s, a mass range of 300-1500 m/z and profile mode data capture. MS2 data were acquired in the ion trap using a 1.2 m/z isolation window, 2E4 AGC target, 300 ms maximum injection time (inject ions for all available parallelisable time "Universal Method"), CID of 35% collision energy, 10 ms activation time and centroid mode data capture.

Mass spectrometry data analysis

Acquired raw files were analyzed in MaxQuant v1.6.0.13. SILAC quantification on light labels (K0 and R0) and heavy labels (K8 and R10) using multiplicity 2 setting was performed. The SwissProt *Homo sapiens* protein database (downloaded July 2017; 20,226 protein entries) was searched. Oxidation of methionine and acetylation of protein N-term were permitted as variable modifications and carbamidomethylation of cysteine was selected as a fixed modification. 1% false discovery rate at the protein and peptide level was selected. The proteinGroups text file was opened in Perseus v1.4.0.2 to permit further data analyses.

For the ASCC1, ASCC2, and ASCC3 interactomes (Figures S4A–S4D), HEK293 Fip-In T-Rex parental cells, or HEK293 Fip-In T-Rex HEK293 cell lines expressing Flag-HA-tagged ASCC1, ASCC2, or ASCC3 were cultured in SILAC light media or heavy media for 2 weeks. Typically, cells grown in eight 150mm dishes in light or heavy media were used for each sample. Cells were harvested and resuspended in 8 mL buffer A and incubated in cold room for 1 hour, followed by centrifugation at 20.000 g for 20 minutes at 4°C. Insoluble fractions were resuspended in 600 μl buffer B and incubated on ice for 20 minutes. The salt concentration was diluted to 150 mM with 1.4 mL buffer C and centrifuged at 20000 g for 10 minutes at 4°C. The supernatant was collected as chromatin fraction and pooled with soluble fraction as whole cell extract, which was in total 10 ml. Whole cell extract was divided into two equal portions and incubated with 100 μl Protein G Agarose (Thermo Fisher Scientific, 20398) or ANTI-FLAG M2 Affinity Gel (Sigma-Aldrich, A2220) at 4°C for 4 hours. Beads were washed with 3 mL buffer A at 4°C for 10 minutes three times, and then eluted with 100μl buffer A containing 1 μg/μl 3xFLAG peptides at 4°C for 1 hour. For mass spectrometry, light isotope labeled samples and heavy isotope labeled samples were mixed before being loaded for SDS-PAGE. For example, light-labeled ASCC1 samples purified by Protein G resins was mixed with heavy-labeled ASCC1 samples purified by ANTI-FLAG M2 Affinity Gel; heavy-labeled ASCC1 samples purified by Protein G resins was mixed with light-labeled ASCC1 samples purified by ANTI-FLAG M2 Affinity Gel. The mixed samples were run around 10 mm into a 10% NuPAGE Bis-Tris gel and stained with Instant Blue (Expedeon, ISB1L).

Sample preparation prior to mass spectrometry analysis, Mass spectrometry data acquisition, and Mass spectrometry data analysis was performed as described for cGAS interactomes, though data acquisition was performed with one amendment: peptides were separated using an 80-minute gradient using the same increasing organic solvent (8 to 32%). ASCC candidate interactors with log2 value more than 1.5 were considered reliable hits. In two parallel experiments, candidates with a log2 (M2 beads VS mock beads) - Log2 (mock beads VS M2 beads) value of more than 3 were thus considered reliable and are listed in Table S1.

Cytosol extraction for examination of cGAS-ribosome interaction

In Figures 3A and S5D, U2OS cells or HEK293T cells transfected with plasmids expressing GFP-tagged cGAS WT or mutants were washed with cold PBS twice, and then collected and spun at 500 g for 5 minutes at 4°C. Cell pellets were resuspended in cytosol buffer (50 mM HEPES, pH 7.4, 100 mM KOAc, 5 mM $Mg(OAc)_2$, 0.01% digitonin, 40 U/ml RNase inhibitor (Thermo Fisher Scientific, N8080119), 1 mM DTT, and protease inhibitor cocktail), and disrupted using 26G needle with 1 mL pre-chilled syringe. Cytosol was cleared by centrifugation at 15000 g for 10 minutes at 4°C, and then subjected to sucrose gradient fractionation and western blotting

CellPress
OPEN ACCESS

analysis (for cytosol extracted from HEK293T, supernatant after centrifugation was incubated for 20 minutes at 37°C prior to sucrose gradient fractionation).

Ribosome purification from HEK293

Generally, four 150 mm dishes of HEK293 cells at 80% confluency were used. After wash with cold PBS twice, cells were collected and resuspended in 1.5 mL cytosol buffer (50 mM HEPES, pH 7.4, 100 mM KOAc, 5 mM $Mg(OAc)_2$, 0.01% digitonin, 40 U/ml RNase inhibitor (Thermo Fisher Scientific, N8080119), 1 mM DTT, and protease inhibitor cocktail), and then disrupted mechanically by passage through a pre-chilled 26G needle using a 5 mL syringe. Cellular debris were cleared by centrifugation at 4°C for 15 min at 15000 g. For DNase-treated ribosomes, the supernatant was collected and subjected to DNase treatment (2.5U/mL Turbo DNase at 25°C for 13 mins). The supernatant was collected and the concentrations of KOAc and $MgAc_2$ in the supernatant were increased to 500 mM and 15 mM, respectively. NP-40 was also added to a final concentration of 0.2% to disrupt ribosome-associated proteins. 300ul sample was then layered over the 1 mL sucrose cushion (20 mM HEPES pH 7.4, 500 mM KOAc, 15 mM $MgAc_2$, 0.1 mM EDTA pH 7.4, 1 M sucrose) and centrifuged at 100,000 RMP for 60 minutes at 4°C in a TLA100.3 rotor (Beckman Coulter, 349490) with 3.5 mL polycarbonate tubes (Beckman Coulter, 349622). The supernatant was removed carefully by aspirator, and the pellets were washed with 100 μl RNC buffer (50 mM HEPES, pH 7.4, 100 mM KOAc, 5 mM $Mg(OAc)_2$) and resuspended with 30 μl RNC buffer. Before measuring the concentration of ribosomes by A260 with NanoDrop, ribosomes from different tubes were pooled. Ribosomes were aliquoted and flash-frozen in liquid nitrogen and stored at −80°C.

Recombinant cGAS purification

GST-tagged cGAS or GST/His-tagged cGAS/hPrimpol1 Proteins were overexpressed in BL21 (DE3) *E. coli* (New England Biolabs, C600003) by growing them at 16°C for 16-18 hours after induction with 0.25 mM IPTG. Cells were lysed by sonication in lysis buffer (20 mM HEPES pH 7.5, 400 mM NaCl, 10% glycerol, Protease Inhibitor cocktail, and 1 mM DTT), and then clarified by centrifugation at 20000 rpm for 30 minutes at 4°C. The supernatant was collected and bound to Glutathione agarose (Thermo Fisher Scientific, 16101) at 4°C for 4 hours, and then was washed with lysis buffer prior to digestion with 1.4 U/ml GST-tagged PreScission Protease (GenScript, Z02799) at 4°C overnight. The supernatant was collected and was separated on 1 mL Heparin HiTrap column (GE Life Sciences, GE17-0407-01) using a linear gradient of 400–1000 mM NaCl (for cGAS) or 100mM-1000mM (for hPrimpol1). Proteins were collected and dialyzed with dialysis buffer (20 mM HEPES-KOH PH 7.5, 250 mM KCl, 1 mM DTT) for three times for 2 hours. Protein was concentrated to 3-4 mg/ml and stored at −80°C for biochemical studies.

Sucrose gradient fractionation

200 μl 10%–50% sucrose gradients were prepared in 7 × 20 mm centrifuge tubes (Beckman Coulter, 343775) by layering 40 μl of 50%, 40%, 30%, 20%, and 10% sucrose (w/v) successively in RNC buffer, and then allowed to stand for 1 hour at 4°C. 20 μl of cytosol fractionation or *in vitro* cGAS-ribosomes binding reaction was layered on the top of 200 μl 10%–50% sucrose gradients, and then spun at 50000 rpm for 16 min at 4°C using a TLS-55 rotor (Beckman Coulter) with the slowest acceleration and deceleration settings in Beckman Optima Max Ultracentrifuge. Eleven 20 μl fractions were collected from the top and subjected to western blot analysis.

In vitro cGAS and ribosome binding assay

Typically, 20 nM cGAS was incubated with 20 nM ribosomes in the RNC buffer at 37°C (for ribosomes purified from HEK293 cells) or 32°C (for ribosomes purified from RRL) for 20 minutes, and then subjected to western blotting analysis in Figures 3C or 5B, respectively. In Figure 3B, 15 μl HisPur Ni-NTA magnetic beads (Thermo Fisher Scientific, 88832) were pre-immobilized with 1 μM 8his-tagged cGAS or hPrimpol1 in 100 μl RNC buffer containing 40 mM imidazole at 4°C for 1 hour, and then washed with 500 μl RNC buffer containing 40 mM imidazole to clean free cGAS or hPrimpol1. Nickel beads immobilized with cGAS or hPrimpoll, or nickel beads were incubated with 100 nM ribosomes (purified from HEK293 cells) in 50 μl RNC buffer containing 40 mM imidazole at 4°C for 1 hour, and then washed with 500 μl RNC buffer containing 40 mM imidazole twice. Beads were eluted with 30 μl elution buffer (20mM Tris-HCl 8.0, 500mM NaCl, 10 mM Magnesium acetate and 300 imidazole), which was subjected to western blotting analysis.

Cyclic dinucleotide synthesis assays

In Figure S6A, cGAS was incubated with different concentrations of HT-DNA (Herring Testis DNA) (Sigma-Aldrich, D6898-250MG) in 20 μl reaction buffer containing 50 mM Tris-HCl pH 7.5, 100 mM NaCl, 10 mM $MgCl_2$, 1 mM DTT, 25 μM ATP, 25 μM GTP, and [α-32^P] ATP (1 μCi) at 37°C for 1 hour. In Figure 4C, cGAS and ribosome (purified from HEK293), or cGAS alone (control) were incubated at 37°C for 20 minutes prior to adding into reaction buffer with HT-DNA. Reactions were terminated by heating at 95°C for 3 min, and subsequently incubated with 0.5 U/μl of alkaline phosphatase (New England Biolabs, M0290) at 37°C for 30 minutes to hydrolyse remained NTPs. 1.5-2 μl of each reaction was spotted on a TLC PEI-Cellulose F plate (Merck Millipore, 105579) and was separated with the use of 1 M $(NH4)_2SO_4$/1.5 M KH_2PO_4 pH 3.8. Radiolabelled products were detected by Typhoon FLA 7000 (GE Healthcare) and quantified with Fiji. The graphs (Figures 4D, 4F, and S6B) were created by GraphPad prism 7.

RNA interference

Generally, cells were successively transfected with siRNAs twice to increase knockdown efficiency and harvested for different analyses 72 hours after the second transfection. 20 μM siRNA was mixed with Lipofectamine RNAiMAX (Thermo Fisher Scientific, 13778150) at a 1:2 (v/v) ratio according to the manufacturer's protocol, and the final concentration of siRNAs in antibiotic-free medium was 20 nM.

TT_{chem}-seq

TT_{chem}-seq were carried out as described previously (Gregersen et al., 2020). Briefly, biological duplicates were generated for each condition. MRC5VA cells at one 15-cm dish were used for each sample and transfected with non-targeted or ASCC3#1 siRNA (Table S3) prior to *in vivo* labeling of nascent RNA by a final concentration of 1 mM 4SU (Glentham Life Sciences, GN6085) pulse for 15 min. Labeling was stopped by TRIzol (Thermo Fisher Scientific, 15596026) and RNA extracted accordingly to the manufacturer's instructions. As a control for equal sample preparation, we spiked- in *S. cerevisiae* (strain BY4741, *MATa, his3D1, leu2D0, met15D0, ura3D0*) 4-thiouracil (4TU)-labeled RNA. *S. cerevisiae* were grown in YPD medium overnight, diluted to an OD600 of 0.1, and grown to mid-log phase (OD600 of 0.8) and labeled with 5 mM 4TU (Sigma- Aldrich, 440736) for 6 min. Total RNA was extracted using the PureLink RNA Mini kit (Thermo Fisher Scientific, 12183020) following the enzymatic protocol. For purification of 4SU labeled RNA, 100 mg mammalian 4SU labeled RNA was spiked-in with 1/100 of 4TU-labeled *S. cerevisiae* RNA. The 101 mg RNA (in a total volume of 100 μl) was fragmented by addition of 20 mL 1 M NaOH and left on ice for 20 min to obtain RNA fragments between 200-500 nt. Fragmentation was stopped by addition of 80 mL 1 M Tris pH 6.8 and cleaned up twice with Micro Bio-Spin P-30 Gel Columns (BioRad, 7326223) according to the manufacturer's instructions. Biotinylation of 4SU-residues was carried out in a total volume of 250 ml, containing 10 mM Tris-HCl pH 7.4, 1 mM EDTA and 5 mg MTSEA biotin-XX linker (Biotium, BT90066) for 30 min at room temperature in the dark. RNA was then purified by phenol:chloroform extraction, denatured by 10 min incubation at 65°C and added to 200 mL mMACS Streptavidin MicroBeads (Miltenyi, 130-074- 101). RNA was incubated with beads for 15 min at room temperature and beads applied to a mColumn in the magnetic field of a mMACS magnetic separator. Beads were washed twice with pull-out wash buffer (100 mM Tris-HCl, pH 7.4, 10 mM EDTA, 1 M NaCl and 0.1% Tween20). 4SU-RNA was eluted twice by addition of 100 mL 100 mM DTT and RNA cleaned up using the RNeasy MinElute kit (QIAGEN, 74204) using 1050 mL 100% ethanol per 200 mL reaction after addition of 750 mL RLT buffer to precipitate RNA < 200 nt. The amount of 4SU-labbled RNA and the size of fragments were confirmed by bioanalyzer prior to library preparation. Libraries for RNA sequencing were prepared using the strand-specific TruSeq total RNA kit (Illumina) using 5 min 65°C fragmentation incubation to anneal primers but to prevent further fragmentation of the samples. The libraries were then sequenced (76bp single-end) on an Illumina HiSeq 4000.

TT-seq data were processed using previously a published protocol (Gregersen et al., 2020). Genes were counted against human GRCh38 Ensembl release-89 using the "summariseOverlaps" function from the Bioconductor package GenomicAlignments (Lawrence et al., 2013). DESeq2 (Love et al., 2014) was used for statistical testing of differential expression between replicate groups, replacing the default size factors with those obtained from a similarly processed yeast counts matrix (sacCer3 Ensembl 89). Gene Set Enrichment Analysis against the Reactome database was conducted using the Bioconductor package FGSEA (Subramanian et al., 2005).

Reverse transcription quantitative PCR (qRT-PCR)

Total RNA from MRC5VA, U2OS cells transfected with indicated siRNA was extracted using the RNeasy kit (QIAGEN, 74104). HEK293T cells do not express STING, so in Figures S7A and S7E, HEK293T cells stably expressing HA-Flag-tagged STING were harvested for RNA extraction 16 hours after transfection with plasmids containing GFP-tagged vector, cGAS WT, or cGAS mutants using Lipofectamine 2000 Transfection Reagent (Thermo Fisher Scientific, N8080234, 11668019) according to the manufacturer's instructions. In Figures S7C and S7G, GFP-tagged vector, cGAS WT, or cGAS mutants was induced to express by adding doxycycline (Clontech, 8634-1) with a final doxycycline concentration of 100 ng/ml in Flp-In T-REx U2OS *CGAS* KO-16 cells for three days before harvesting. In Figure 6F, Flp-In T-REx U2OS parental cells or *CGAS* KO-16 cells were treated at 43°C for 45 minutes and then allowed to recover for 18 hours at 37°C prior to harvesting. In Figure 6G, cells were also treated with 10 μg/ml cycloheximide (CHX) or DMSO for 8 hours before harvesting. In Figure S7J, MRC5VA cells were treated with ethidium bromide at the final concentration of 100 ng/ml for 96 hours before harvesting. Extracted RNA was DNase (QIAGEN, 79254) treated. The RNA concentration was measured using NanoDrop ND-1000 (Thermo Fisher Scientific). ~1μg RNA in 20 μl reaction was used for reverse transcription reaction using TaqMan Reverse Transcription Reagents (Thermo Fisher Scientific, N8080234). Random hexamers were used for the reverse transcription reaction. cDNA was amplified in CFX384 Touch Real-Time PCR Detector (Bio-Rad, 1855485) using iTaq Universal SYBR Green Supermix (Bio Rad, 172-5124) with the following conditions: 39 cycles of 10 s denaturation at 95°C, and 30 s annealing at 60°C. Primers amplifying mature RNA of GAPDH were used as internal control. All primer sequences are listed in Table S3. The data was analyzed using GraphPad Prism 7.

Generation of collided ribosome and purification from rabbit reticulocyte lysate (RRL)

In Figure 5B, *In vitro* translation in RRL and purification of recombinant $eRF1^{AAQ}$ were performed as previously described (Sharma et al., 2010; Juszkiewicz et al., 2018). 100 μl of non-nucleased RRL with a final concentration of 1mM $eRF1^{AAQ}$ (for collided ribosomes generation) or without $eRF1^{AAQ}$ (control) was used to set up 200 μl *in vitro* translation reaction. The reactions proceeded for 45 min at

32°C, and then were transferred to 1 mL sucrose cushion (20 mM HEPES pH 7.5, 500 mM KOAc, 15 mM $MgAc_2$, 0.1 mM EDTA pH 7.4, and 1 M sucrose) in 13×51mm polycarbonate centrifuge tube (Beckman Coulter, 349622) and spun for 1 hour at 100000 rpm in TLA100.3 rotor at 4°C. The supernatant was removed carefully by aspirator and the pellets were washed with 100 μl RNC buffer (50 mM HEPES, pH 7.4, 100 mM KOAc, 5 mM $Mg(OAc)_2$) and resuspended with 15 μl RNC buffer. Ribosomes from different tubes were pooled and concentration was measured at absorbance 260 nm (A260). Purified ribosomes were aliquoted and flash-frozen in liquid nitrogen and stored at −80°C.

Digestion of polysomes with nuclease

In Figure 5C, one 150mm dish of MRC5VA cells 80% confluency was collected and washed with cold PBS twice, and resuspended with 100 μl lysis buffer (50 mM HEPES, pH 7.4, 100 mM KOAc, 5 mM $Mg(OAc)_2$, 0.5% Triton, 1 mM DTT, and protease inhibitor cocktail) and incubated in ice for 15 minutes. Cell debris were removed by centrifugation at 15000 g for 10 minutes at 4°C. The supernatant was collected, and the concentration of RNA was measured by Qubit (Thermo Fisher Scientific). 40 μg of RNA in a total volume of 60 μl was digested with 20 U Micrococcal Nuclease (New England Biolabs, M0247S) with 1 mM final concentration of $CaCl_2$ at 25°C for 45 minutes and terminated by adding 0.3 μL of 500 mM EGTA, and then subjected to sucrose gradient fractionation and western blotting.

Flow cytometry analysis

Cells were dissociated from vessel by trypsinization and collected by centrifugation at 500 g for 5 minutes. Cells were resuspended in PBS with 2% FBS and 200 ng/ml DAPI. Cell suspensions were diluted to $1x10^6$ cells/ml and filtered through a 35 μm nylon strainer in order to remove clumps. 10,000 events per condition were recorded on a BD LSR II and analyzed in FlowJo.

Immunofluorescence staining

U2OS cells expressing functional GFP-tagged cGAS (Figures S8E and S8F) in place of endogenous cGAS (Flp-In T-REx U2OS *CGAS* KO16-GFP-cGAS-WT) were used for assessing cGAS localization. In Figure 6A, U2OS KO-12-GFP-cGAS-WT cells were transfected with the indicated siRNAs according to siRNA interference protocol and then seeded on coverslips in a 6-well plate two days before harvesting. In Figure 6B, cells were treated with high concentration anisomycin (400 ng/ml), low concentration anisomycin (20 ng/ml), low concentration emetine (20 ng/ml), or high concentration emetine (500 ng/ml) for 8 hours before harvesting. In Figure 6C, cells were treated at 43°C for 45 minutes and then were recovered for 6-7 hours at 37°C prior to harvesting. In Figure S3C, MRC5VA or U2OS cells were irradiated by UV-C (20J/m^2, recovery for 3 hours). In Figure S8D, cells were treated with 50 μg/ml cycloheximide 30 minutes prior to heat shock treatment (43°C for 45 mins).

All cells were harvested and fixed with 3% paraformaldehyde in PBS for 10 minutes at room temperature. This was followed by permeabilization with 100% methanol at −20°C for 5 minutes (Figures 6A–6C and S8D) or otherwise with 0.5% Triton X-100 (20mM HEPES PH7.4, 50mM NaCl, 3mM MgCl2, 300 mM Sucrose, and 0.5% Triton X-100). Cells were washed with 1 mL PBS for twice and blocked with PBS containing 5% goat serum for 30 minutes at room temperature. Primary antibodies (eS8, 1:2000, Ab201454; γH2AX, 1:1000, Sigma-Aldrich; dsDNA, 1:1000, ab27156) were diluted in blocking buffer (PBS containing 5% goat serum) and incubated in cold room overnight. Cells were washed with 1 mL PBS and incubated with secondary antibody in blocking buffer (Goat anti-Rabbit Alexa Fluor 594, Cat # A-11012 or Goat anti-Mouse Alexa Fluor 594 Cat # A-11005, 1:500. Thermo Fisher Scientific) for 30 minutes at room temperature. After the final wash cells were stained with DAPI and mounted on glass slides with antifade mounting medium (Vector Laboratories, H-1700). Images were captured with Olympus FV3000-Invert Scanning Laser Microscope, using a PlAPON 20× dry, 40× dry, or 60× Oil objective with excitation at 405 (for DAPI), 488 (for GFP), and 594 nm (for Alexa Fluor 594), and then pseudo-colored using Fiji open source software.

Proximity ligation assay (*in situ* PLA)

Proximity ligation assay was carried out following the instructions of Duolink *In Situ* Red Starter Kit Mouse/Rabbit (Sigma-Aldrich, DUO92101). Briefly, U2OS *CGAS*KO stably expressing Flag-HA-tagged cGAS cells were seeded 8-well Glass slides (PEZGS0816, Millipore, 30000 cells per well) the day before heat shock treatment. Cells were treated 43°C for 45 minutes. Cells were fixed with 3% paraformaldehyde for 10 minutes at room temperature after 7 hours recovery at 37°C, then permeabilized for 5 minutes with 0.5% Triton X-100 (20mM HEPES PH7.4, 50mM NaCl, 3mM MgCl2, 300 mM Sucrose, and 0.5% Triton X-100). The following steps were performed according to instructions of Duolink® Proximity Ligation Assay Kit. Cells were incubated with primary antibodies (Flag, 1:1000, Sigma-Aldrich, F1804; es8, 1000, Ab201454) at toom temperature for 1 hour. Images were captured with Olympus FV3000-Invert Scanning Laser Microscope, using a PlAPON 40× objective with excitation at 405 (for DAPI) and 594 nm (for Texas Red), and then pseudo-colored and analyzed using Fiji opensource software.

cGAMP measurement *in vivo*

0.5 million *CGAS*-KO7 or parental MRC5VA cells were seeded in 6 cm dishes and transfected with the relevant siRNAs. For the DNA-transfected positive control, one 10cm dish of 60% confluent MRC5VA cells was transfected with 7 μg Herring Testes DNA (Sigma-Aldrich, D6898-250MG) with lipofectamine 2000, or lipofectamine alone as control. Cells were washed twice in ice cold PBS, centrifuged for 3 minutes at 500 g, snap frozen in liquid nitrogen, and stored at −80. Cell pellets were resuspended in appropriate amounts of lysis

buffer (20 mM TRIS-HCl, pH 7.5, 150mM NaCl, 2.5mM MgCl2, 1% NP40, protease inhibitors, and phosphatase inhibitors). Lysates were centrifuged 15 minutes at max speed in an Eppendorf centrifuge, and the supernatant was transferred to a new 1.5 mL tube. Protein concentration was determined using Bradford assay and samples were diluted in lysis buffer in order to obtain equal protein concentrations. The remaining steps were performed according to the manufacturer's instructions in the 2′,3′-Cyclic GAMP Direct EIA Kit (2Bscientific, K067-H1).

Micronucleus measurement

In Figure S3E, U2OS cells were transfected with indicated siRNAs according to siRNA interference protocol and then seeded on coverslips in a 6-well plate two days before harvesting. Cells were harvested and fixed with 3% paraformaldehyde in PBS for 10 minutes at room temperature, and then washed with PBS and stained with DAPI. The percentage of cells with micronuclei was counted manually under blinded conditions. Images were visualized with Olympus FV3000-Invert Scanning Laser Microscope, using a PlAPON 40× dry objective with excitation at 405 (for DAPI).

QUANTIFICATION AND STATISTICAL ANALYSIS

DESeq2 (Love et al., 2014) was used for statistical testing of differential expression (TT-Seq). In Figures 2C and S4B, mass spectrometry data were analyzed by Perseus version 1.4.0.11. In Figure S3A, cGAS-KO genotype was analyzed by TIDE. In Figures S4G and S4F, 10000 events per condition were recorded and analyzed in FlowJo. The experiments were repeated twice, and a representative result is shown. The rest of data analysis was performed using GraphPad Prism 7. Error bars of RT-qPCR experiments represent standard deviation (SD) of three technical replicates and were calculated from three technical replicates of each biological sample. All RT-qPCR experiments were repeated at least twice, and a representative result is shown. In Figures 4A, 4C, 4E, and S6A the radiolabelled signal was exposed to phosphor imager and scanned using Typhoon FLA 7000. Densitometry was quantified by Fiji. Error bars represent SD of two biological replicates. In Figure S1H, error bars represent standard error of the mean (SEM) of three technical replicates. In Figure S3D, 200 cells were analyzed per condition per experiment, and error bars represent SEM of three biological replicates. In Figure S3E, 400 cells were analyzed per condition per experiment, and error bars represent SEM of six biological replicates. In Figures S8A–S8C, 200 cells were analyzed for each sample at one experiment. Error bars represent SD of three biological replicates. In Figure 6E, error bars represent SD of puncta per cell from 80 cells per condition. Statistical significance was determined using two-tailed t test; *$p < 0.05$; **$p < 0.01$; ***$p < 0.001$; ****$p < 0.0001$; NS, not significant ($p > 0.05$).

Molecular Cell, Volume *81*

Supplemental information

Translation stress and collided ribosomes

are co-activators of cGAS

Li Wan, Szymon Juszkiewicz, Daniel Blears, Prashanth Kumar Bajpe, Zhong Han, Peter Faull, Richard Mitter, Aengus Stewart, Ambrosius P. Snijders, Ramanujan S. Hegde, and Jesper Q. Svejstrup

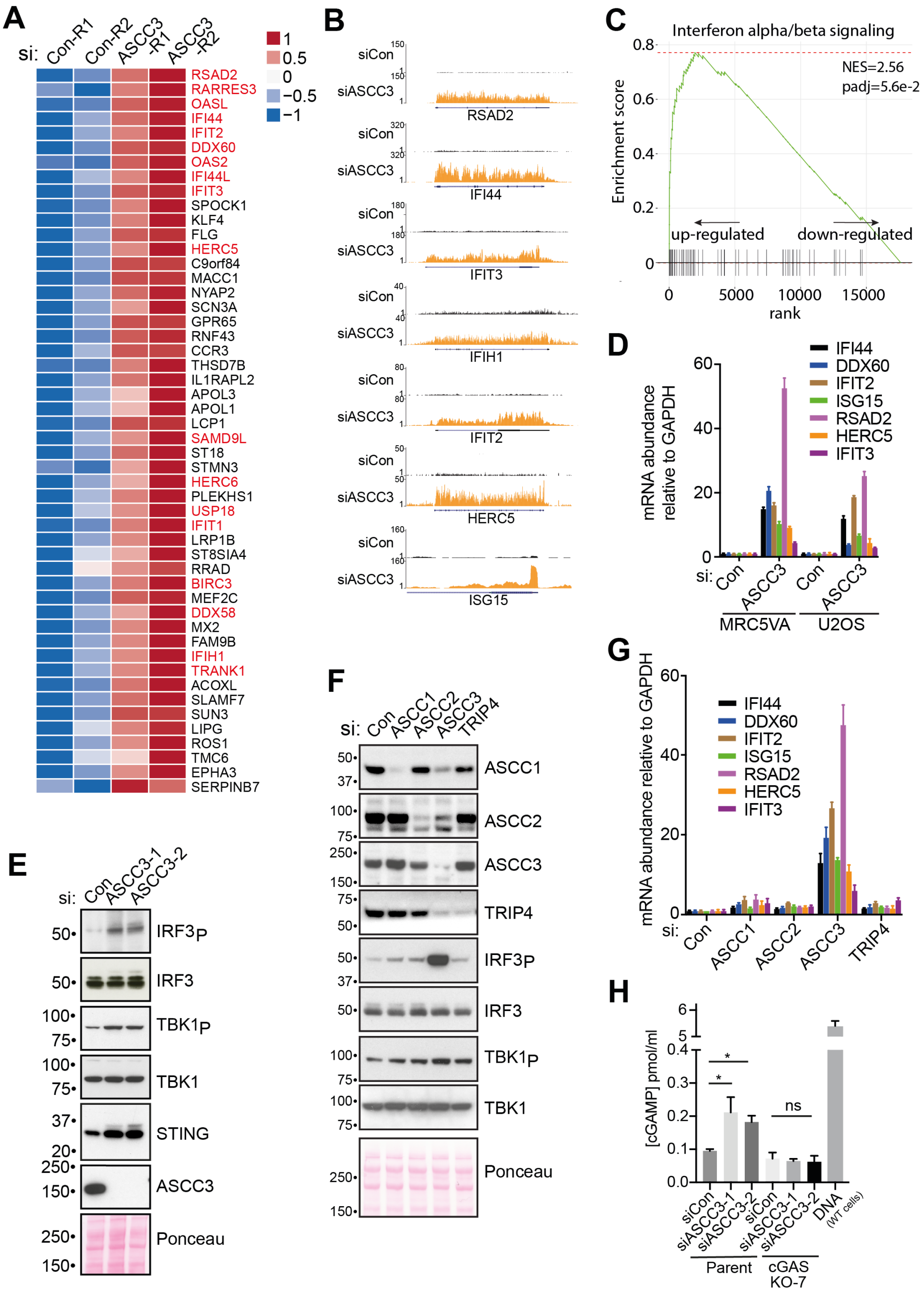

Figure S1. Knockdown of ASCC3 induces the expression of ISGs. Related to Figure 1. A. Heat map of the 50 most up-regulated genes at the level of nascent transcription upon ASCC3 siRNA knockdown (ASCC3kd) in MRC5VA cells. R1, Replicate 1; R2, Replicate 2. The heatmap represents a z-score of normalised expression. ISGs are marked in red. B. UCSC genome browser views of nascent transcription at individual ISGs. Read depth coverage was combined across two replicates, normalised to yeast spike-in. C. GSEA category enrichment plot of the Reactome category 'Interferon alpha/beta signalling'. NES, normalised enrichment score; padj, adjusted p-value. D. qRT-PCR analysis of ISG mRNA expression in MRC5VA or U2OS cells after ASCC3kd. Error bars represent standard deviation (SD) of three technical replicates and are representative of three biological repeats. E. Western blot analysis of IRF3p (ser396), IRF3, TBK1p (ser172), STING ASCC3 protein level in MRC5VA cells transfected with ASCC3 siRNAs. F. Western blot analysis of MRC5VA cells after siRNA knockdown of ASCC subunits. G qRT-PCR analysis of relative ISG expression from the same cells as in F. Error bars represent SD of three technical replicates and are representative of three biological repeats. H. ELISA analysis of cGAMP concentration in MRC5VA parental or cGAS KO cells transfected with the indicated siRNAs. Two-tailed t-test. *p < 0.05; ns: not significant. Error bars represent SEM (three replicates). MRC5VA parental cells transfected with HT-DNA served as a positive control.

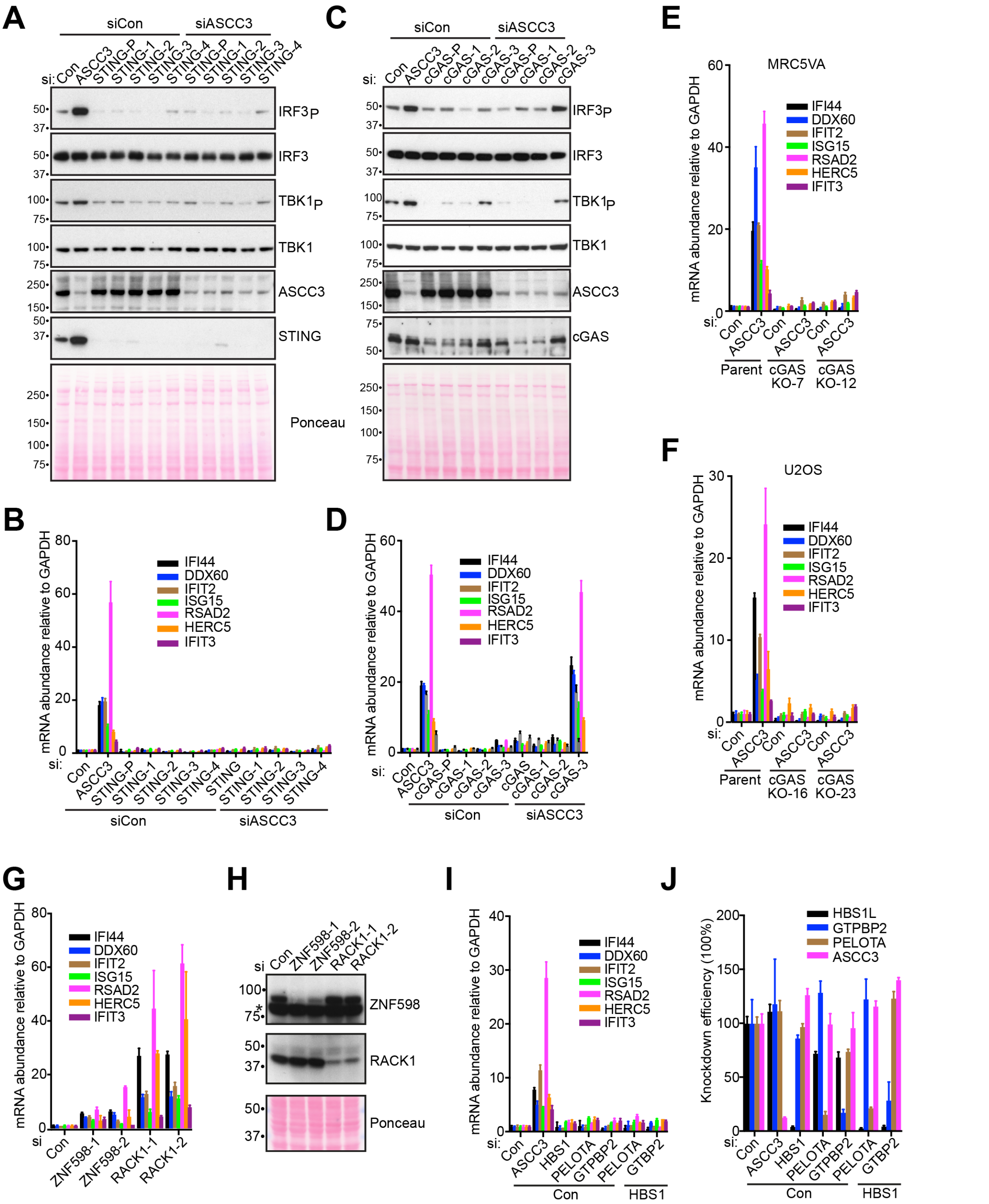

Figure S2. cGAS and STING are required for activation of ISGs. Related to Figure 1. A. Western blot analysis of MRC5VA cells after siRNA knockdown. STING-P, STING siRNA pool. **B.** qRT-PCR analysis of ISG expression in the same cells as in A. Error bars represent SD of three technical replicates and are representative of three biological repeats. **C.** As in A, but transfection with different siRNAs. cGAS-P, cGAS siRNA pool. **D.** As in B, but transfection with different siRNAs. **E.** qRT-PCR analysis of ISG expression in parental and *CGAS* knockouts (KO-7 and -12) MRC5VA cells, after ASCC3 knockdown. Error bars as in B. **F.** As in E, but in U2OS cells (parental versus *CGAS* KO-16 and -23). **G.** qRT-PCR analysis of ISG expression in U2OS cells transfected with two different ZNF598 or RACK1 siRNAs. Error bars as in B. **H.** Western blot analysis of ZNF598 and RACK1 protein levels in the same cells as in G. Asterisk denotes a non-specific band. **I.** qRT-PCR analysis of ISG expression in U2OS cells transfected with siRNAs. Error bars as in B. **J.** qRT-PCR analysis of knockdown efficiency for the indicated genes in the same cells as in I. Normalized to GAPDH mRNA as internal control.

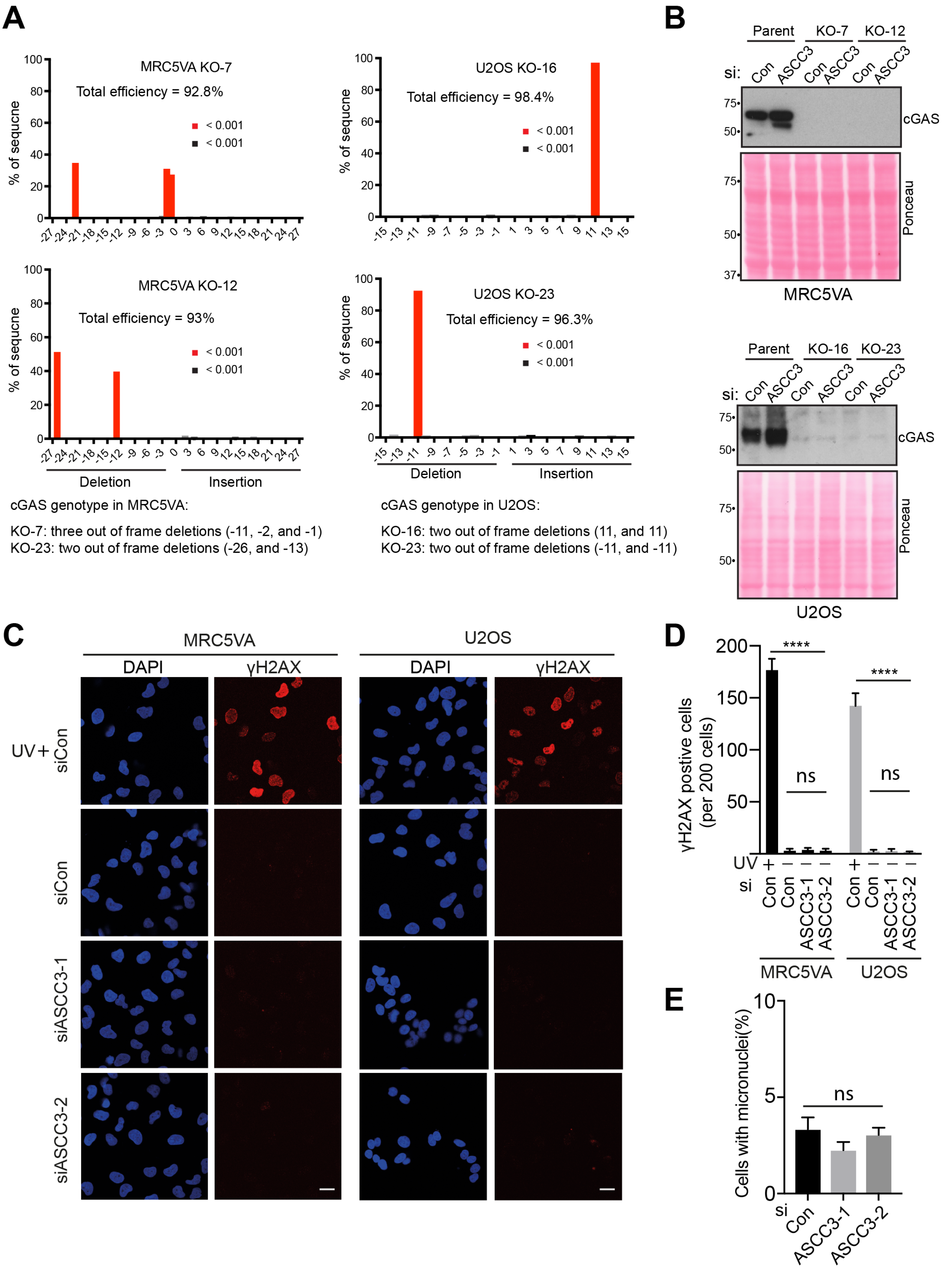

Figure S3. cGAS KO and lack of effect of ASCC3 on DNA damage markers. Related to Figure 1. **A.** Tracking of Indels by TIDE analysis of cGAS knockouts in MRC5VA or U2OS cells. **B.** Western blot analysis of the cGAS knockouts. **C.** MRC5VA or U2OS cells were transfected with ASCC3 siRNAs. Cells were fixed, and stained with anti-γH2AX antibody and with DAPI, and imaged by confocal fluorescence microscopy. Scale bar: 10 μm. **D.** Quantitative analysis of gH2AX-positive cells from C. Mean with SEM, 200 cells analysed per condition per experiment, n=3 independent experiments. Two-tailed t test, ns, no significant; ****, $P < 0.0001$. **E.** The Percentage of U2OS cells containing micronuclei. Mean with SEM, 400 cells analysed per condition per experiment, n=6 independent experiments. Two-tailed t test. ns, not significant.

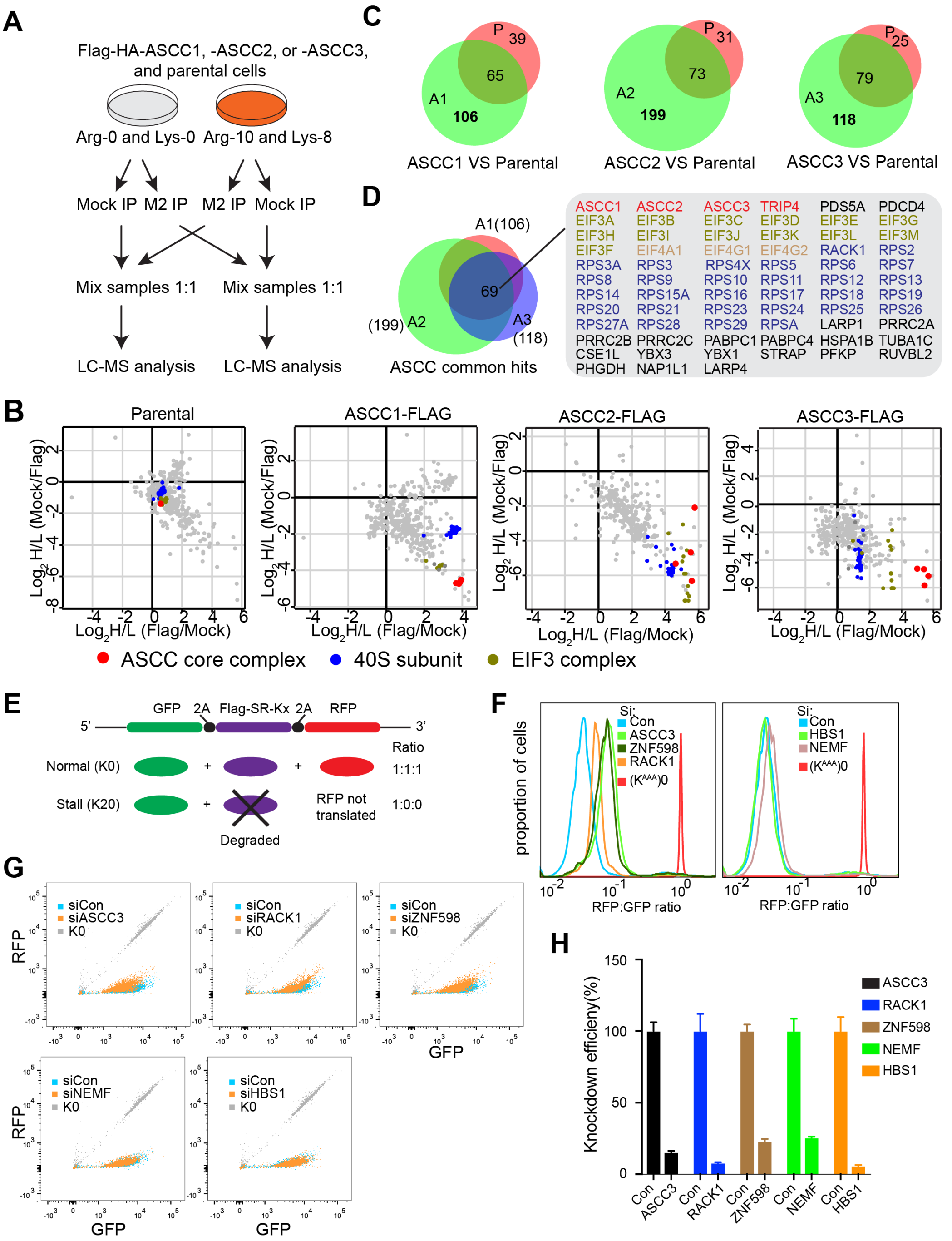

Figure S4. ASCC interactome, and effect of RQC factors on translation. Related to Figure 1. A. Strategy of SILAC-based quantitative mass spectrometry. **B.** Scatter plots showing interactomes in control (parental cells), Flag-HA-ASCC1, -ASCC2, and -ASCC3 HEK293 cells. Perseus was used for all proteomic data analysis. **C.** Overlap of interactors between control and experimental cells; hits from the ASCC1, ASCC2, or ASCC3 interactomes that were not found in the control were used for further analysis in D. See STAR Methods for details and lists of hits in Table S1. **D. Left,** Venn diagram and **Right,** the 69 overlapping hits from the ASCC1, ASCC2, and ASCC3 interactomes. ASCC subunits, small ribosomal proteins, and EIF3 complex subunits are indicated. **E.** Reporter construct and expected protein products with or without stalling signal. Flag-SR-Kx is a stalling sequence in which x represents either twenty or zero lysines. **F.** Histograms of the RFP:GFP ratios quantified by flow cytometry in the $(K^{AAA})_{20}$ HEK293 cells transfected with indicated siRNA. $(K^{AAA})_{0}$ cells were used as a reference. For simplicity, cells transfected with siRNAs for control, ASCC3, ZNF598, or RACK1 are shown in the left panel, while those for HBS1, or NEMF are shown on the right. **G.** Scatterplot showing individual $(K^{AAA})_{20}$ HEK293 cells transfected with indicated siRNAs. $(K^{AAA})_{0}$ cells were used as a reference. **H.** RT-qPCR analysis of knockdown efficiency for experiments in F and G. Error bars represent SD of three technical replicates and are representative of two biological replicates.

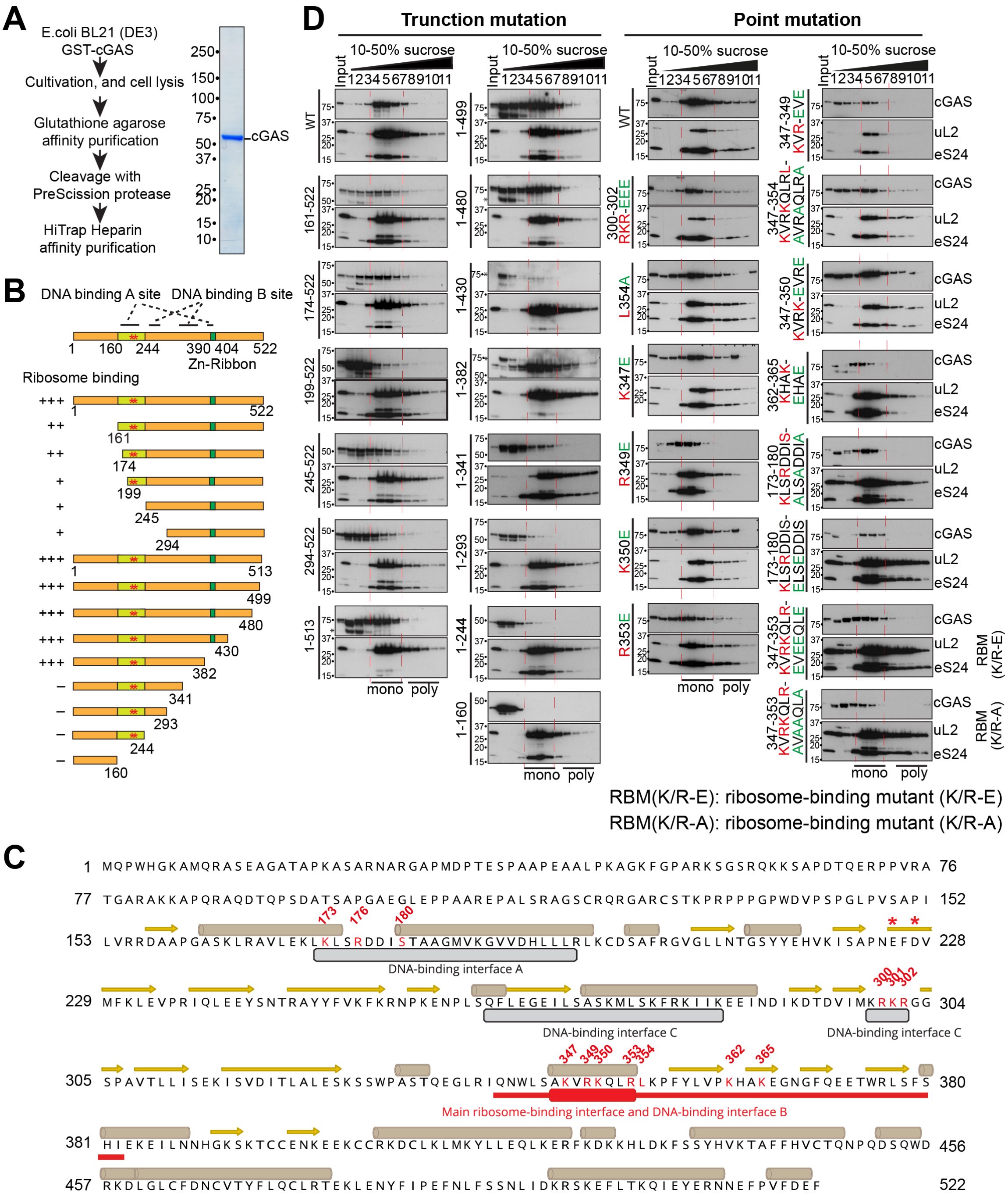

Figure S5. Purification of cGAS and characterization of its ribosome binding domains. Related to Figure 3. A. Diagram for purification; and Coomassie staining of purified cGAS. **B.** Schematic of cGAS deletion mutants for testing the interaction with ribosomes. Red asterisks indicate the active site. +++ , strong binding; ++, medium binding; +, weak binding; -, little or no binding. **C.** Sequence of cGAS and point mutants for testing its interaction with ribosomes. Charged amino acids marked in red were mutated to either alanine (A) or glutamic acid (E). The region most important for cGAS-ribosome interaction is indicated by a red line, and a key area containing several important K and R residues is shown as a red box. Red asterisks (E_{225} and D_{227}) indicate the active site, while the light brown tubes indicate alpha-helices and the yellow arrows beta-sheets. **D.** Western blot analysis of sucrose gradient sedimentation of cGAS-ribosome interactions. Fractions were immunoblotted for GFP-cGAS and for representative ribosome subunits (ul2 and eS24). Asterisks denote non-specific bands. Note that while K/R point mutations in regions 173-180 and 300-302 have little or no effect, mutation of residues in 362-365 and especially 347-353 results in reduced binding. Mutation of positively charged residues in 347-353 to alanine in the context of full length cGAS results in marked decrease in binding so that the binding profile is similar to cGAS 1-341. These mutations also abolish ISG activation upon ASCC3 knockdown.

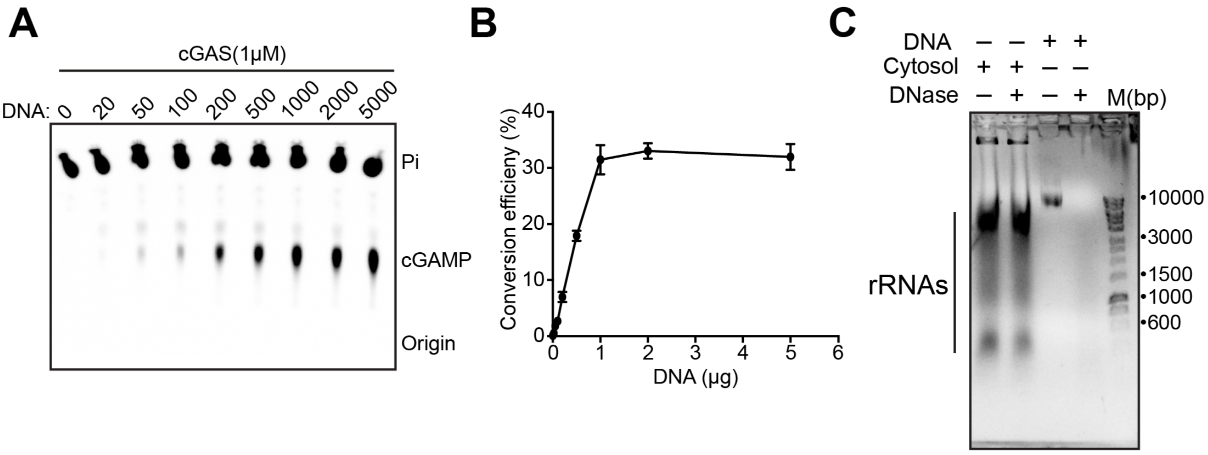

Figure S6. cGAS assays and controls. Related to Figure 4. A. Autoradiograph of cGAS-mediated cGAMP synthesis in the presence of different concentrations of herring testis DNA. **B.** Quantification of data in A by Fiji. Error bars indicate standard deviation (SD) of duplicate replicates. cGAMP signal was normalized to pi signal. **C.** Gel electrophoresis of HEK293 cytosol or 200 ng of HT-DNA digested with 0.05 U TURBO DNase under the conditions used for purifying DNase-treated ribosomes. Note that there is, not surprisingly, no DNA is detectable by this approach in the cytosol, so that no difference between treated or untreated cytosol can be observed.

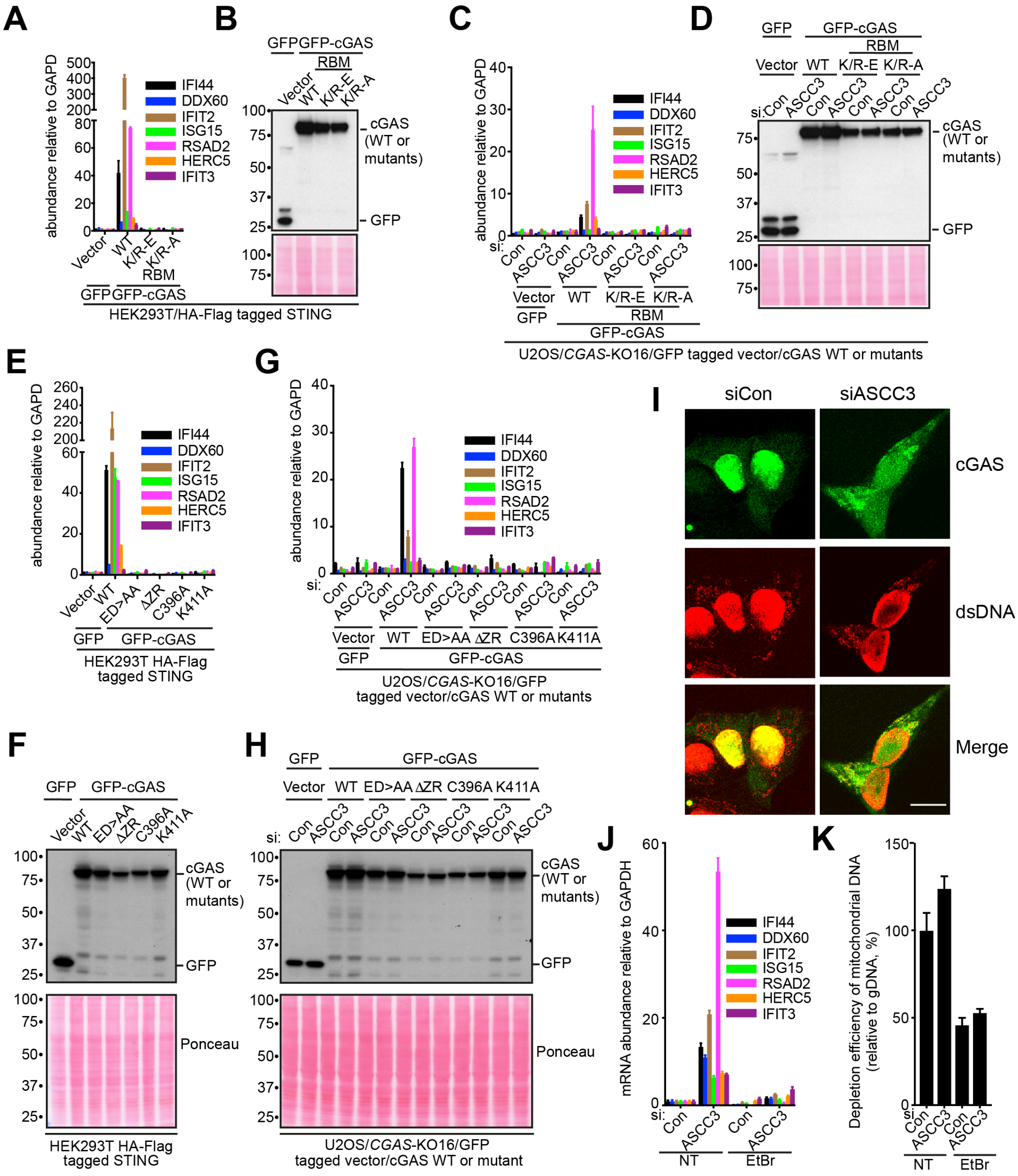

Figure S7. The ribosome-binding interface, zinc-ribbon domain and DNA binding domain of cGAS remain critical for cGAS activation in ASCC3-deficienct cells. Related to Figure 4. A. ISG expression analysis by qRT-PCR of HEK293T cells stably expressing HA-Flag-tagged STING, after transfection of plasmids encoding GFP-vector control, GFP-tagged wild type cGAS (WT), or cGAS ribosome-binding mutants: RBM (K-E) or RBM (K-A) referring to Figure S5D. See STAR Methods for details. **B.** Western blot analysis of whole cell lysate from A to check protein expression levels using anti-GFP antibody. **C.** qRT-PCR analysis of ISG expression in U2OS *CGAS* KO-16 cells and the effect of expression of different cGAS versions, with or without ASCC3 knockdown as indicated. **D.** Western blot analysis of whole cell lysate from C to check protein expression levels using anti-GFP antibody. **E.** As in A, but transfection of plasmids encoding GFP-vector control, GFP-tagged wild type cGAS (WT), or the indicated cGAS: ED>AA (E225A and D227A) is an active site mutant; ΔZR (Δ390-404) deletes the zinc ribbon domain, and C396A is a zinc-ribbon domain point mutant, K411A is a DNA binding site mutant. **F.** Western blot analysis of whole cell lysate from E to check protein expression levels using anti-GFP antibody. **G.** As in C, but stable expression of different cGAS mutants, with or without ASCC3 knockdown as indicated. **H.** Western blot analysis of cell lysates from G, as in F. **I.** U2OS cells stably expressing GFP-cGAS were transfected with ASCC3 siRNA. Cells were fixed, stained with antibody detecting double-stranded DNA, and imaged by confocal fluorescence microscopy. Scale bar: 10 µm. Note the staining in the cytoplasm. **J.** qRT-PCR analysis of ISG expression in MRC5VA cells treated with ethidium bromide (EtBr, 100 ng/ml for 96 hours), after ASCC3 knockdown. **K.** qRT-PCR analysis of depletion efficiency of mitochondrial DNA in the same cells as in J. gDNA, genomic DNA.

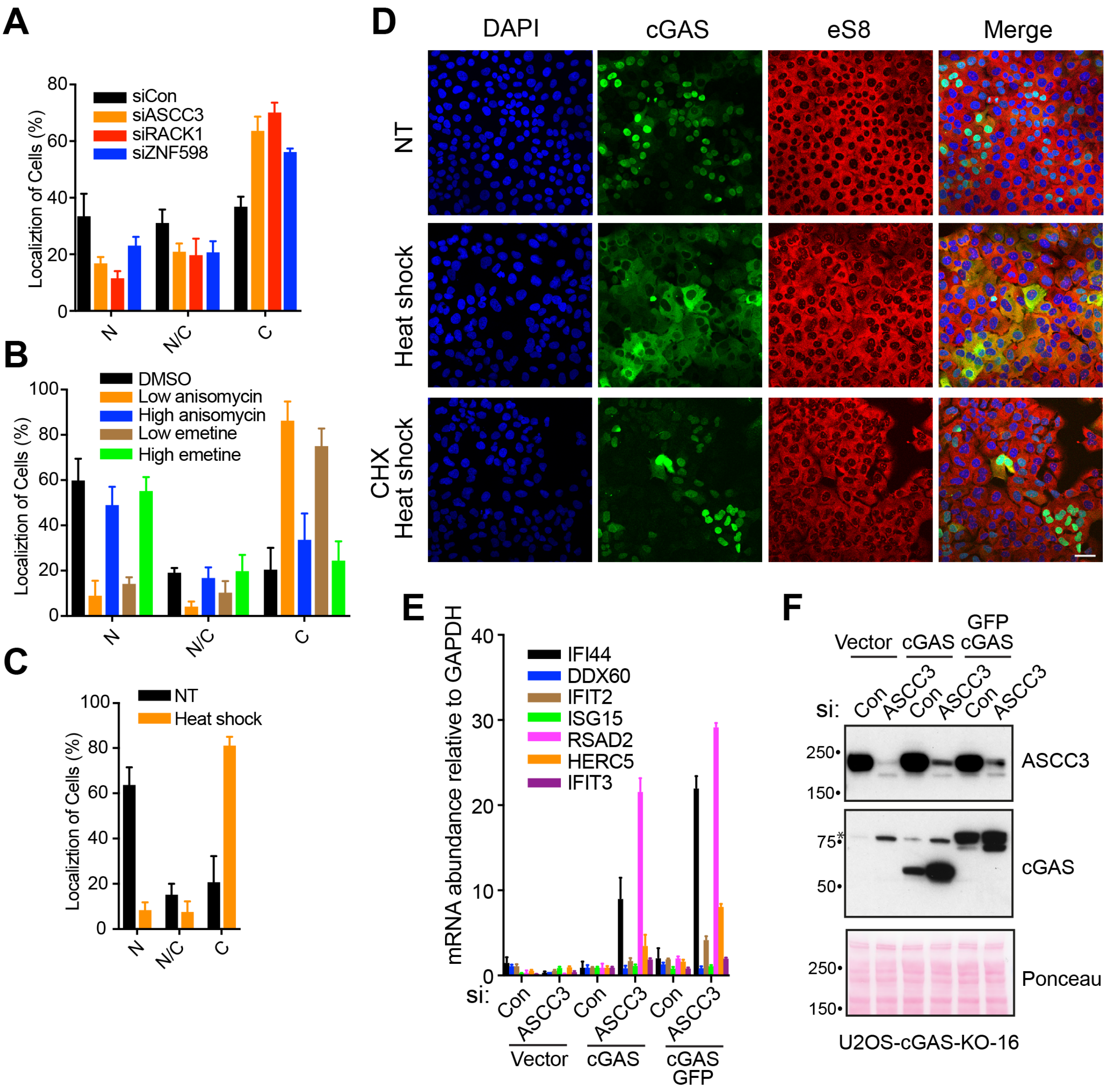

Figure S8. cGAS preferentially binds to collided ribosomes and becomes cytosolic upon ribosome collision. Related to Figure 6. A. Quantitative analysis of the subcellular localization of GFP-cGAS from Figure 6A. 200 cells were analyzed for each sample, error bars represent SD of three biological replicates. N, nucleus; N/C, nucleus and cytosol; C, cytosol. **B.** As in A., but analyzing the data in Figure 6B. **C.** As in A., but analyzing the data in Figure 6C. **D.** U2OS *cGAS* KO cells stably expressing GFP-tagged cGAS were treated with heat shock in the absence or presence of cycloheximide (CHX). Cells were fixed, and stained with eS8 antibody and with DAPI, and imaged by confocal fluorescence microscopy. Scale bar: 50 µm. **E.** qRT-PCR analysis of relative ISG expression in U2OS *CGAS* KO stably expressing untagged or GFP-tagged cGAS cells transfected with ASCC3 siRNA. Error bars represent SD of three technical replicates and are representative of three biological replicates. **F.** Western analysis of ASCC3 and cGAS in the same cells as in E. Asterisk denotes a non-specific band.
